# An SNP-based saturated genetic map and QTL analysis of fruit-related traits in Zucchini using Genotyping-by-sequencing

**DOI:** 10.1186/s12864-016-3439-y

**Published:** 2017-01-18

**Authors:** Javier Montero-Pau, José Blanca, Cristina Esteras, Eva Ma. Martínez-Pérez, Pedro Gómez, Antonio J. Monforte, Joaquín Cañizares, Belén Picó

**Affiliations:** 1 Institute for the Conservation and Breeding of Agricultural Biodiversity (COMAV-UPV), Universitat Politècnica de València, Camino de Vera s/n, 46022 Valencia, Spain; 2 Instituto de Investigación y Formación Agraria y Pesquera (IFAPA). Área de Mejora y Biotecnología de cultivos. Camino San Nicolás 1, 04745 La Mojonera, Almería Spain; 3 Instituto de Biología Molecular y Celular de Plantas (IBMCP), Universitat Politècnica de València (UPV)-Consejo Superior de Investigaciones Científicas (CSIC), Ciudad Politécnica de la Innovación (CPI), Ed. 8E, C/Ingeniero Fausto Elio s/n, 46022 Valencia, Spain

**Keywords:** *Cucurbita pepo*, RIL, GBS, Cartography, Phenotyping, Candidate genes

## Abstract

**Background:**

*Cucurbita pepo* is a cucurbit with growing economic importance worldwide. Zucchini morphotype is the most important within this highly variable species. Recently, transcriptome and Simple Sequence Repeat (SSR)- and Single Nucleotide Polymorphism (SNP)-based medium density maps have been reported, however further genomic tools are needed for efficient molecular breeding in the species. Our objective is to combine currently available complete transcriptomes and the Zucchini genome sequence with high throughput genotyping methods, mapping population development and extensive phenotyping to facilitate the advance of genomic research in this species.

**Results:**

We report the Genotyping-by-sequencing analysis of a RIL population developed from the inter subspecific cross Zucchini x Scallop (ssp. *pepo* x ssp. *ovifera*). Several thousands of SNP markers were identified and genotyped, followed by the construction of a high-density linkage map based on 7,718 SNPs (average of 386 markers/linkage group) covering 2,817.6 cM of the whole genome, which is a great improvement with respect to previous maps. A QTL analysis was performed using phenotypic data obtained from the RIL population from three environments. In total, 48 consistent QTLs for vine, flowering and fruit quality traits were detected on the basis of a multiple-environment analysis, distributed in 33 independent positions in 15 LGs, and each QTL explained 1.5–62.9% of the phenotypic variance. Eight major QTLs, which could explain greater than 20% of the phenotypic variation were detected and the underlying candidate genes identified.

**Conclusions:**

Here we report the first SNP saturated map in the species, anchored to the physical map. Additionally, several consistent QTLs related to early flowering, fruit shape and length, and rind and flesh color are reported as well as candidate genes for them. This information will enhance molecular breeding in *C. pepo* and will assist the gene cloning underlying the studied QTLs, helping to reveal the genetic basis of the studied processes in squash.

**Electronic supplementary material:**

The online version of this article (doi:10.1186/s12864-016-3439-y) contains supplementary material, which is available to authorized users.

## Background

*Cucurbita pepo* L. is an economically important species of the Cucurbitaceae family cultivated worldwide, with more than 24 million tons produced in 2013 and nearly 1.8 million ha cultivated [1]. It is particularly important in Asian, American and Mediterranean countries, being Mexico and Spain the main worldwide exporters. Like other cucurbits, *C. pepo* has become a model to study sex expression, fruit set and development, and parthenocarphy [2, 3].

Taxonomically, the species is divided into three subspecies, *C. pepo* ssp. *pepo* L., *C. pepo* ssp. *ovifera* (L.) Decker (also known as ssp. *texana* (Scheele) Filov), both of them including cultivated varieties, and *C. pepo* ssp. *fraterna* (L.H. Bailey) Lira, Andres & Nee that is considered as a true wild subspecies [4, 5]. The domestication occurred at least twice in Southern USA and Northern Mexico, where the cultivar diversification was initiated. An additional diversification process occurred after the European contact with the New World and the subsequent migration mainly to Mediterranean countries. Nowadays, this species displays a high variability for many agronomic traits, such as fruit shape and color, flowering habit, leaf morphology, etc. [5, 6].

Cultivars are classified in eight horticultural groups (ssp. *pepo*: Pumpkin, Vegetable Marrow, Cocozelle and Zucchini, and ssp. *ovifera*: Scallop, Acorn, Crookneck, and Straightneck). The “Zucchini” group rank among the highest-valued vegetables worldwide [7]. Cultivars from Zucchini, Vegetable Marrow and Cocozelle groups, all of them producing elongated fruits, are considered as modern cultivars being newly developed in Europe, whereas the round and flattened Pumpkins and Scallops are ancient groups developed after domestication in North America [8].

Despite the agricultural and biological interest of this species, knowledge of its genetics and genome has been very limited in comparison with other cucurbits [9–11]. Recently, we produced the first transcriptome of the species, from root, leaves, and flower tissues, by using the 454 sequencing technology [12] that was later significantly improved with Illumina technology by sequencing cDNA from additional tissues (shoot apices, floral buds, and pre-harvest and postharvest fruit subjected to ethylene, methylcyclopropene and cold treatments). The *C. pepo* transcriptome v3 is available at the Cucurbigene website [13]. Genes were annotated and classified in different biological functions. Some of them have been recently used to study biological processes in *Cucurbita* [2, 14].

The transcriptome sequencing was focused on two varieties with contrasting phenotypes, representing the two *C. pepo* main subspecies, the inbreeding line ‘MU-CU-16’, belonging to the Zucchini morphotype (*C. pepo* ssp. *pepo*), the main summer squash sold in European markets, and the inbreeding line ‘UPV-196’ of the Scallop morphotype (ssp. *ovifera*). These genotypes also represent different domestication and diversification steps, Scallop types were already selected by Native Americans, whereas elongated Zucchini were selected in Italy after the introduction of this species in Europe [15]. Recently, other transcriptomic studies have been carried out with specific purposes, for instance, to identify genes involved in aphid infestation [16], or to develop markers in Pumpkin types [17].

Dense genetic maps are necessary tools for efficient molecular breeding. In the past decades, the linkage maps of the *Cucurbita* genus were constructed using populations derived from both inter (*C. pepo* x *C. moschata*) and intra-specific crosses (*C. pepo* ssp. *pepo* x ssp. *pepo*, and *C. pepo* ssp. *pepo* x ssp. *ovifera*). These maps were first composed of dominant markers (Random Amplified Polymorphic DNA (RAPD) and Amplified Fragment Length Polymorphisms (AFLP)) [18–20], and later completed with SSRs [21, 22]. The two genotypes, Zucchini and Scallop, used to generate the *C. pepo* transcriptome were also selected previously as parents of an F_2_ mapping population that was employed to construct the first SNP-based genetic map of the species and to map several QTLs (Quantitative Trait Loci) involved in plant, flowering and fruit traits [23].

The SNPs located in the map by Esteras et al. [23] were selected among those identified *in silico* by mining the transcriptomic sequences of the F_2_ parents (‘MU-CU-16’ and ‘UPV-196’), and were validated using a Golden-Gate genotyping platform. Despite this map produced valuable information, its marker density was only moderate (6.02 cM/marker). New methods integrating simultaneously SNP discovery and genotyping such as Genotyping-by-sequencing (GBS) [24, 25] can be applied to rapidly develop high-density genetic maps.

The aim of this work is to generate a high-density genetic map using several thousands of SNPs obtained by the GBS analysis of a new Recombinant Inbred Line population (RIL), developed through single seed descent from the previous Zucchini x Scallop F_2_ population [23]. RIL populations are more adequate for genetic mapping as a higher number of recombinations are produced, improving the mapping resolution, and they can be replicated by seed, making easy replicated trials that facilitates a better estimation of QTL effects [26]. Consequently, we also investigated the genetic control of economically important traits as vine, flowering and fruit quality traits, by QTL analysis, taking advantage of the new high-density map and of the first draft of the *C. pepo* genome available at the Cucurbigene website [13]. The results presented herein will help to establish a molecular breeding system in this species.

## Methods

### Generation of the RIL population

Two genotypes of *C. pepo* were used as parentals to produce the intra-specific RIL mapping population used in this study, belonging to different subspecies, ‘MU-CU-16’ is a Zucchini type of the subspecies *pepo* and ‘UPV-196’ is a Scallop type of the subspecies *ovifera*. The two parents present contrasting phenotypes for vine, flowering and fruit traits detailed in [23]. F_1_ plants produced from the Zucchini x Scallop cross were selfed to generate the F_2_ population used to construct the first SNP-based map in *C. pepo* [23]. F_2_ individual plants were selfed until the F_8_ generation by single seed descent. A final set of 120 F_8_ RILs was obtained.

### DNA isolation

DNA was isolated from young leaves of plants of each of the 120 RILs. Additionally, three replicated samples of parentals and the F_1_ generation and two independent DNA extractions of two randomly selected RIL families were included as controls. DNA extraction was carried out using DNeasy Plant Mini Kit (Qiagen, Hilden, Germany). DNA concentrations were measured with Nanodrop ND-1000 Spectrophotometer v.3.5 to select samples with at least 50 ng/μl. Finally, 131 samples with high-quality DNA were selected for sequencing. Samples were sent for genotyping to the Genomic Diversity Facility at Cornell University (Ithaca, New York, USA). A minimum of 500 ng of DNA were used for SNP genotyping.

### Genotyping-by-sequencing

A Genotyping-by-sequencing approach was used for SNP discovery between the parents and among the RILs as described by Elshire et al. [24]. The GBS libraries from all samples were prepared using *ApeKI* endonuclease (recognition site: G/CWGC) and sequenced using the Illumina HiSeq 2000 platform (Illumina Inc, San Diego, CA, USA). GBS libraries were constructed including the parents and F_1_ (three replicates each) and 122 RIL samples. GBS sequencing reads were de-multiplexed according to the sample barcodes and adapter sequences were removed using GBS barcode splitter [27]. Reads were trimmed by base Phred quality (Q score < 25) and reads shorter than 30 base pairs were discarded before mapping and SNP calling.

The filtered, high-quality sequences from each sample were aligned to the last version of the *C. pepo* genome v.3.2 [13] using Bowtie 2 with default parameters [28]. SNP calling was performed using Freebayes [29]. A minimum mapping quality of 30, minimum base quality of 20 and minimum coverage of 5 was required. Resulting SNPs were additionally filtered discarding those with more than 70% of missing data, no biallelic, with a Minimum Allele Frequency < 10%, or with heterozygosity >70%. Genotypes with a quality lower than 10 were also discarded.

### Phenotyping of the RIL population

The seeds of 120 F_8_ RILs were germinated in Petri dishes and transplanted to pots (in glasshouses) and to soil (under tunnel) at 2 weeks after germination in three independent assays. The first assay was performed from February to July 2014 (assay Paip2014) at the tunnel facilities of CAJAMAR (Paiporta, Valencia, Spain). Three plants per RIL were phenotyped. DNA for GBS analysis was obtained from this trial. The other two assays were conducted in 2015, at the same tunnel facilities (Paip2015, from February to July) and in the glasshouse facilities at the Polytechnic University of Valencia (UPV) (Valencia, Spain, from May to October, UPV2015). In these two assays, also three sister plants were phenotyped per RIL with a fully randomized experimental design.

In the three assays we measured traits related to vine growth, plant morphology, flowering and fruit traits. Forty-three traits were measured for each single plant (Additional file 1). Vine traits were related to plant length (growth habit, plant length, and number of nodes of the plants at the end of the assay), branching intensity, and leaf traits (spines in the leaf petiole, leaf incision, and occurrence of silver leaf); and flowering traits were related to the flowering time (first node with male/female flower, and days from transplanting to the development of the first male/female flower).

Each plant was selfed and two fruits per plant were analyzed. One of them at immature stage, 7 days after pollination, which corresponds to the commercial stage of summer squashes. The second fruit was analyzed at physiological maturity (ranging from 30 to 60 days after pollination). Traits measured at both physiological states were immature/mature peduncle length, fruit size and shape (fruit length, width, shape, weight, number of locules and ribbing), flesh firmness, rind and flesh color, and sugar content and acidity. More details about all measured traits are included in Additional file 1.

Data from the assays performed in the three locations were used to calculate pairwise genetic correlations between locations as r x,y = covariance x,y/√(variance x * variance y), where x and y represented two different locations [30].

### Genetic map construction and QTL detection

A genetic map was constructed using R packages R/qtl [31] and ASMap [32]. ASMap package implements the MSTmap algorithm [33], which is an extremely fast algorithm for linkage map clustering and ordering of thousands of markers. SNPs were coded as homozygotes similar to one parent (A), to the other (B) or heterozygotes (H) using custom scripts. Polymorphic *loci* that were heterozygous in any of the parents, markers with more than 10% of missing data or duplicated markers (markers with the same genotype for all individuals) were discarded. To remove possible genotyping errors, we used our own implementation of the SMOOTH algorithm [34]. This method corrects the genotype of an individual based on neighboring markers. *mstmap* function from ASMap package was used to cluster and order markers and *quickEst* to estimate genetic map distances using Kosambi map function [35]. To detect segregation distortion, Chi-square (χ2) tests were computed for each SNP using R/qtl function *geno.table* and *p*-values were corrected for multiple testing using Benjamini and Yekutieli correction [36]. Highly distorted and unlinked markers were excluded from analysis. Mapchart 2.2 [37] was used to visualize a constructed map for each linkage group (LG). The new map was compared with the previous *C. pepo* 384-SNP map developed with the F_2_ population derived from the same Zucchini x Scallop cross [23]. This set of SNPs were located in the *C. pepo* genome v.3.2 using BLAST with their flanking sequences in order to obtain an equivalence between Esteras’ genetic map and current genome version. Linkage groups were named according to the RIL map generated in the current study.

Once the genetic map was established, SNPs showing segregation distortion were located in the physical map together with the mapped SNPs to identify genomic regions with genetic distortion.

### QTL mapping

QTL analysis for each trait was performed by Composite Interval Mapping (CIM) using the R/qtl function *cim* with a scan window size of 20 cM and 20 background marker *loci* as QTL cofactors. A multi environment search of QTLs was performed using the data from the three assays. Environmental effects and Genotype x Environment (G x E) interactions were estimated for each trait using a two factor Analysis of Variance (ANOVA), with all the phenotypic data. Also an additional QTL analysis was performed per environment, using data of each assay separately. Logarithm of odds (LOD) threshold for a Type I error *P* < 0.05 and *P* < 0.01 value was obtained based on a permutation test (1000 permutations were run per trait). LOD support interval was calculated using R/qtl *lodint* function using a 1.5 LOD units drop. The additive QTL effect (a) and the proportion of phenotypic variance explained by QTL (R^2^) were estimated at the highest QTL peaks using R/qtl function *fitqtl*. QTLs exceeding the threshold value (*p* < 0.01) in this analysis were considered significant.

## Results and discussion

### Sequence data and SNP discovery

A total of 242.4 million cleaned reads with a total of 21.7 Gb were generated for the parents, F_1_ and the 122 RIL samples. The number of reads obtained varied from 0.9 million to 4.6 million with an average of 1.85 million reads per line. The genome of *C. pepo* v3.2 has a total assembled size of 263,500,453 bp (with 909 large scaffolds > = 10 Kb). All the cleaned reads represent an average percentage of covered genome with a read depth > 1 and > 20 of 3.0% and 0.5%, respectively (Additional file 2). Sequences have been submitted to the Sequence Read Archive (SRA) of the NCBI (SRR4299463-SRR4299615, BioPoject PRJNA344022).

The sequences obtained were filtered and used for SNP identification. A 93.9% of the cleaned reads were mapped to the *C. pepo* genome and 62,617 SNPs were identified after the SNP calling with Freebayes. After excluding SNPs that were monomorphic in the RIL population, those non biallelic, with more than 70% of missing data, with heterozygosity >70% or with Minimum Allele Frequency (MAF) < 10%, 26,430 SNPs remained. The SNPs identified were again filtered to remove heterozygous SNPs in parents.

The number of SNPs identified in this population is higher than the number identified in a recent GBS analysis performed using an F_2_ population derived from the cross between two accessions of the closely related species *Cucurbita maxima* [38]. Using the same filters employed in the study by Zhang et al. [38], that is, selecting SNPs with less than 20% missing data and MAF ≥ 0.2, we identified about eight times more SNPs in our RIL population, 16,222 SNPs in *C. pepo* RIL population *versus* 1,881 in *C. maxima* population. Differences can be explained by the higher variability of the *C. pepo* species and by the fact that we have crossed two accessions belonging to two different subspecies generated in two independent domestication events [5].

### Construction of genetic map

A genetic map was constructed using genotypic data of the RIL population. After discarding those SNPs with more than 10% of missing data, and those showing a statistically significant deviation from Mendelian segregation, 10,166 SNPs distributed in 178 scaffolds (representing 212,381,440 bp, 80.6% of the genome) remained. We found 3,676 SNPs forming groups of SNPs with the same genotype for all samples. Only one SNP per group was retained. We also removed unlinked markers and SNPs that had different genotypes in the two DNA replicates used as controls. The average degree of heterozygosity existing in this F_8_ RIL population was 1.47% (ranging from 0.013 to 3.72%).

**Fig. 1:**
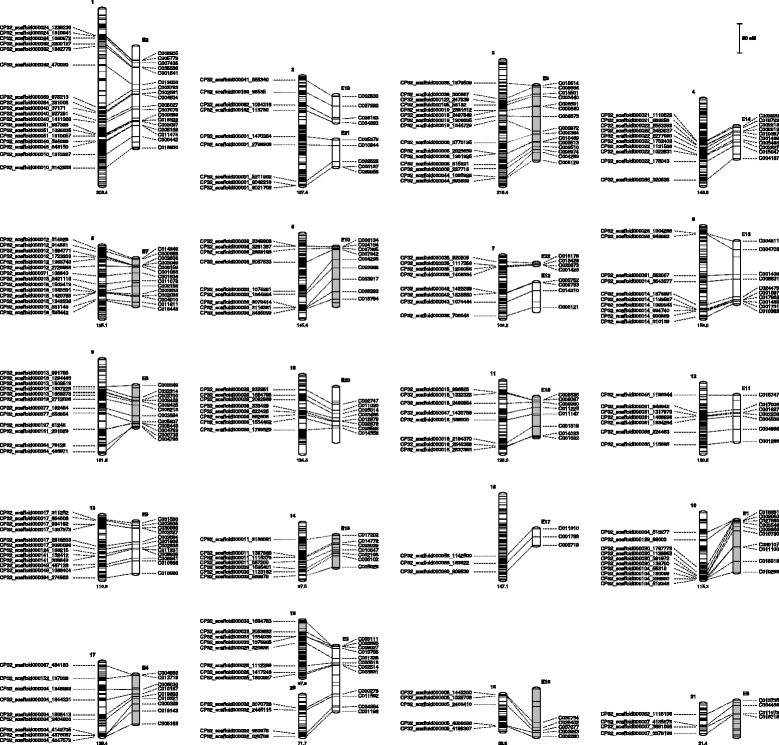
Correspondence between RIL and F_*2*_ maps. Correspondence between the 21 linkage groups of the new map developed with GBS using the RIL population (left bars numbered 1 to 21) and the 22 linkage groups of the previous map constructed with the F_2_ population derived from the same cross (right bars numbered E1 to E22). SNP markers common to both maps are indicated (the name of the SNP markers used in Esteras et al. [23] has been maintained in the LG of this map represented in the figure)

The map consisted of 7,718 SNPs distributed across 21 linkage groups (Table 1, Fig. 1). The individual LGs had between 770 and 101 markers each, with a mean of 367.5 markers per LG. The LG size ranged from 51.4 cM (LG 21) to 303.4 cM (LG 1), giving a total genetic length of 2,817.6 cM. Average genetic distance between successive markers was 0.4 cM, and maximum spacing between markers ranged from 16.8 cM in LG 1 to 4.6 cM in LG 4. A total of 145 scaffolds (from 1 to 16 scaffolds per LG) of the current version of the *C. pepo* genome (version 3.2) could be anchored to the genetic map (Table 1). Additional file 3 (a) includes detailed information about the genetic map with the genetic and the physical position of each SNP marker in the C. *pepo* genome (version 3.2), along with the flanking sequences of all SNPs. All SNPs have unique physical locations in the *C. pepo* genome, and are potentially transferable among species allowing comparative studies within this genus.

**Table 1:** Genetic map of a RIL population of *C. pepo*

LG	N° markers	Length (cM)	Average spacing (cM)	Maximum spacing (cM)	N° of nucleotides (% of total genome)	N° of anchored scaffolds	*C. pepo* v3.2. scaffolds
1	770	303.4	0.4	16.8	21,302,769 (7.95)	16	10, 24, 32, 40, 46, 51, 59, 63, 78, 79, 84, 91, 105, 111, 144, 175
2	575	187.4	0.3	8.8	14,361,414 (5.36)	12	1, 41, 55, 62, 76, 99, 128, 140, 162, 168, 169, 208
3	527	215.4	0.4	10.0	13,761,414 (5.14)	11	6, 19, 38, 44, 117, 118, 122, 163, 187, 195, 210
4	501	148.8	0.3	4.6	10,858,678 (4.05)	8	20, 21, 22, 66, 86, 143, 159, 180
5	448	125.1	0.3	7.1	10,667,745 (3.98)	5	12, 18, 48, 71, 121
6	415	145.4	0.4	9.2	10,134,556 (3.78)	4	3, 8, 89, 101
7	412	108.2	0.3	5.8	10,056,303 (3.75)	8	35, 36, 42, 43, 82, 83, 106, 13127
8	383	158.2	0.4	7.2	9,911,322 (3.70)	6	14, 26, 61, 72, 75, 132
9	371	161.8	0.4	11.4	9,828,092 (3.67)	8	13, 54, 64, 77, 130, 146, 161, 197
10	357	134.5	0.4	15.5	9,823,969 (3.67)	3	9, 29, 68
11	335	125.0	0.4	6.9	9,820,194 (3.67)	4	15, 16, 47, 131
12	332	120.6	0.4	7.8	9,347,089 (3.49)	10	30, 31, 45, 58, 65, 74, 96, 103, 120, 186
13	326	110.8	0.3	7.7	8,951,933 (3.34)	11	17, 49, 53, 88, 94, 107, 108, 113, 124, 141, 153
14	325	97.5	0.3	6.4	8,813,444 (3.29)	2	11, 39
15	317	147.1	0.5	8.3	8,682,934 (3.24)	11	23, 56, 60, 69, 92, 93, 97, 98, 119, 135, 172
16	297	115.3	0.4	11.7	8,672,504 (3.24)	7	28, 30, 34, 80, 85, 104, 129
17	290	128.4	0.4	12.6	8,327,454 (3.11)	8	4, 67, 70, 78, 100, 152, 200, 206
18	289	97.9	0.3	9.6	8,239,682 (3.08)	2	25, 33
19	185	63.8	0.3	7.3	8,114,804 (3.03)	1	5
20	162	71.7	0.4	6.9	7,958,368 (2.97)	2	2, 27
21	101	51.4	0.5	7.0	4,746,772 (1.77)	6	7, 52, 81, 110, 115, 148
Total	7718	2817.6	0.4	16.8	212,381,440 (79.27)	145	

For each linkage group, number of markers, total genetic distance, average and maximum spacing between adjacent markers is shown. Number of scaffolds of the *C. pepo* genome v.3.2 included in each linkage group is also shown

This new map significantly improves the previously reported *C. pepo* map constructed with the F_2_ population in the earlier study by Esteras et al. [23], which included 315 markers covering 1,740.8 cM, with an average distance between markers of 6.02 cM and a maximum gap of 30.3 cM. The sequences of these markers were mapped to the *C. pepo* genome to compare both maps. It was possible to associate most of the linkage groups established in the F_2_ map, with unique LG of the new high density genetic map (Fig. 1). The RIL map enabled merging former LGs 19 and 21, and 22 and 12 (corresponding to new LG 2 and 7 respectively). Also current LG 18 and 20 corresponded with the former LG 3. Therefore, the comparison of both maps revealed 20 linkage groups that corresponded to the 20 chromosomes of *C. pepo*.

The map length is similar to that reported for *C. maxima*, 2,566.8 cM [38], with a much higher marker density, 385.9 *versus* 22.9 marker per LG, an average of 0.4 *versus* 5.6 cM between successive markers, and fewer genetic gaps, making the current map the most saturated genetic map of the *Cucurbita* genus to date.

SNPs with statistically significant distorted segregation that were discarded for map construction, were located in the genome (Additional file 3b). In some regions, distorted SNPs were interspersed with non-distorted SNPs, so the observed distortion was not likely due to genetic reasons, but to artefacts of the GBS analysis or complex genomic causes (i.e., gene duplication or deletion). However, in other regions, blocks of continuous distorted SNPs were observed. In such cases, a real genetic distortion was assumed (Additional file 3c). These regions were mainly located in the distal region of LG 19 (CP32_scaffold000005_127543-2532610 Scallop alleles overrepresented) and of LG 21 and LG 20 (CP32_scaffold000007_32640-4220403 and CP32_scaffold000027 11033-2401349, Zucchini alleles overrepresented). This distortion in the distal region of LG 20 could explain why this LG could not be merged with LG 18 in the RILs map (Fig. 1). Other regions with distorted segregation were for example in LG 16 (28.2–51.4 cM; CP32_scaffold000028_577-1064181), LG 12 (91.9–111.1 cM, scaffold000045_87006-1620317), LG 1 (127.3–129.5 cM, scaffold000084_267107-735527) and LG 4 (24.5 cM, scaffold000086_313524-580307), with Scallop alleles overrepresented, and in LG 15 (9.2 cM, scaffold000060_271409-477442), with Zucchini alleles overrepresented. Also some scaffolds that could not be anchored to the genetic map showed distorted segregation skewed towards Zucchini and towards Scallop alleles, respectively. Some of these regions were also observed in the former F_2_ map, mainly in the previous LG 2 and LG 5 (current LG 1 and 21).

Different functions were associated to the genes located in the distorted regions. Some were directly related to the flowering process (Protein UNUSUAL FLORAL ORGANS: CP32_scaffold000007_1554474, squamosa promoter binding protein-like: CP32_scaffold000045_1030767 and CP32_scaffold000084_557489, Flowering time control protein FPA: CP32_scaffold000045_1196335) [39–41]. Others are transcription factors involved in plant growth and development (Scarecrow-like transcription factor PAT1: CP32_scaffold000050_1278397, TCP family transcription factor: CP32_scaffold000084_653160), in the embryogenesis process (embryo defective 2170: CP32_scaffold000028_353221, Homeobox protein knotted-1-like 7: CP32_scaffold000086_568011) [42, 43] or genes related to hormone metabolism (ethylene or auxin related) (Additional file 3c). Scallop and Zucchini alleles were overrepresented in different regions, suggesting that the alleles in this region may be subjected to gametic or zygotic selection and/or related to preferential germination or better seedling viability. Some of these unigenes may be the cause of the segregation distortion, but it could also be the result of linkage to other genes.

### QTL analysis

Given the low level of heterozygosity observed in the RILs grown in Paip2014 (see above), the GBS genotype for the RILs is adequate for QTL analysis in the three environments after removing the heterozygous *loci*. We performed composite interval mapping with window size of 20 cM. QTL analysis based on genotypic data and phenotypic data for 43 traits, identified a total of 48 QTLs (Table 2) for vine, flowering and fruit traits that were detected with the full set of data and with the data from at least two environments separately. These QTLs were distributed in 33 independent positions in 15 linkage groups. The proportion of the phenotypic variance explained by a single QTL (R^2^) varied from 1.5 to 62.9%. Detailed information about all these QTLs (explained variance, LOD peaks, flanking markers, additive effects) is shown in Table 2. Additional files 4 and 5 include additional data of the analyzed traits and QTLs. Identified QTLs are discussed below grouped by traits.

**Table 2:** QTLs detected for vine, flowering, plant and fruit traits

QTL	Trait	LG	Genetic position (cM)	LOD interval	Flanking markers	Additive_effect	Model.pvalues	R^2^	E.pvalue*	GxE.pvalue**
*Li_10*	*Leaf incision*	10	33.9	61.8–66.9	CP32_scaffold000009_2374010-CP32_scaffold000009_2556718	0.57	4.48E-10	50.05		
*Sl_12*	*Silver leaf*	12	59.3	35.1–39.8	CP32_scaffold000031_1726079-CP32_scaffold000031_2012785	0.19	3.87E-12	23.32		
*Sl_1*	*Silver leaf*	1	132.5	1.9–10.1	CP32_scaffold000084_728121-CP32_scaffold000059_548298	0.06	5.09E-03	3.83	5.77E-03	4.59E-02
*Sl_16*	*Silver leaf*	16	66.1	5.7–10.8	CP32_scaffold000034_1033951-CP32_scaffold000028_1777958	0.11	1.22E-04	8.01	1.67E-02	4.04E-02
*DFeF_12*	*Days to female flowering*	12	33.1	2.6–4.8	CP32_scaffold000074_621895-CP32_scaffold000096_181501	2.71	8.75E-08	7.87	3.00E-09	
*DFeF_9*	*Days to female flowering*	9	116.0	5.2–8.4	CP32_scaffold000013_960688-CP32_scaffold000013_1308585	1.21	1.22E-02	2.61	4.07E-09	3.14E-02
*IFSh_3*	*Immature Fruit shape*	3	173.2	17.7–26.5	CP32_scaffold000038_1385297-CP32_scaffold000038_1853138	0.40	1.57E-14	17.83		
*IFSh_12*	*Immature fruit shape*	12	25.0	21.8–25.4	CP32_scaffold000065_760668-CP32_scaffold000074_729362	0.25	4.77E-06	6.73		
*IFLe_3*	*Immature Fruit length*	3	173.2	30.7–40.2	CP32_scaffold000038_1385297-CP32_scaffold000038_1853138	2.11	2.27E-15	31.79	1.67E-02	
*IFLe_15*	*Immatute fruit length*	15	34.7	7.5–10.1	CP32_scaffold000056_543412-CP32_scaffold000056_920404	0.96	4.79E-06	6.72	4.18E-02	4.40E-02
*IFLe_12*	*Immature fruit length*	12	24.2	8.9–11.7	CP32_scaffold000065_589737-CP32_scaffold000074_729362	1.04	1.07E-06	7.61		
*IFWi_3*	*Immature Fruit width*	3	171.0	10.5–13.9	CP32_scaffold000038_1385297-CP32_scaffold000038_1853138	0.68	3.22E-15	18.68	1.93E-02	
*MFSh_3*	*Mature Fruit shape*	3	171.0	5.5–8.4	CP32_scaffold000038_1385297-CP32_scaffold000038_1853138	0.26	1.72E-09	10.99		
*MFSh_4*	*Mature fruit shape*	4	39.4	5.3–9.3	CP32_scaffold000159_92504-CP32_scaffold000022_375710	0.10	3.43E-02	3.43		
*MFSh_5*	*Mature fruit shape*	5	27.9	9.4–11.3	CP32_scaffold000018_362002-CP32_scaffold000018_549964	0.14	1.98E-03	3.02		9.85E-03
*MFLe_3*	*Mature Fruit length*	3	169.1	46.7–67.4	CP32_scaffold000038_1385297-CP32_scaffold000038_1853138	4.51	2.37E-15	38.71	1.98E-02	
*MFLe_12*	*Mature fruit length*	12	19.3	17.8–20.2	CP32_scaffold000065_389234-CP32_scaffold000065_538115	1.88	3.72E-06	6.64		
*MFLe_6*	*Mature fruit length*	6	19.7	25.6–28.7	CP32_scaffold000003_3022364-CP32_scaffold000003_3087204	1.35	7.97E-04	3.55		
*MFLe_9*	*Mature fruit length*	9	119.3	13.8–23.0	CP32_scaffold000013_890110-CP32_scaffold000013_959962	1.86	7.84E-06	6.21		
*MFWi_3*	*Mature Fruit width*	3	171.0	7.04–13.4	CP32_scaffold000038_1385297-CP32_scaffold000038_1853138	0.77	8.63E-13	15.14	3.71E-04	
*MFWi_12*	*Mature fruit width*	12	24.9	16.7–19.1	CP32_scaffold000065_760668-CP32_scaffold000074_780275	0.55	9.55E-07	7.42	4.31E-04	1.89E-02
*IFRib_3*	*Immature fruit ribbing*	3	69.4	4.1–10.7	CP32_scaffold000006_3196015-CP32_scaffold000006_3616125	0.35	1.73E-07	8.69	2.31E-04	2.16E-02
*MFRib_12*	*Mature fruit ribbing*	12	110.2	14.1–20.5	CP32_scaffold000031_115435-CP32_scaffold000045_1342921	0.23	3.11E-09	10.66	8.62E-03	
*MFRib_21*	*Matre fruit ribbing*	21	19.2	10.8–19.6	CP32_scaffold000007_4220403-CP32_scaffold000110_136233	0.19	6.73E-06	6.19		
*IPeLe_10*	*Immature peduncle length*	10	71.3	10.7–13.1	CP32_scaffold000009_272548-CP32_scaffold000009_704495	0.64	1.32E-06	7.61		
*IPeLe_16*	*Immature peduncle length*	16	14.9	12.9–15.9	CP32_scaffold000030_1135277-CP32_scaffold000030_1337312	0.46	8.94E-04	3.67		
*MPeLe_14*	*Mature peduncle length*	14	29.3	16.7–19.2	CP32_scaffold000039_1662180-CP32_scaffold000011_1038061	0.42	4.67E-04	3.98		
*ILRCo_4*	*Immature rind color, L parameter*	4	14.9	46.5–58.8	CP32_scaffold000066_82542-CP32_scaffold000143_78868	9.86	0.00E + 00	40.64	2.78E-02	
*ILRCo_10*	*Immature rind color, L parameter*	10	99.5	9.02–12.6	CP32_scaffold000029_1388824-CP32_scaffold000029_1802506	3.73	1.65E-05	6.03		
*ILRCo_1*	*Immature rind color, L parameter*	1	75.2	12.8–15.9	CP32_scaffold000046_683368-CP32_scaffold000046_1012066	3.00	8.31E-04	3.67		
*IbRCo_4*	*Immature rind color, b parameter*	4	15.3	20.1–22.9	CP32_scaffold000066_82542-CP32_scaffold000143_130468	2.19	1.59E-12	15.40		
*IbRCo_3*	*Immature rind color, b parameter*	3	62.9	11.5–16.7	CP32_scaffold000006_2344126-CP32_scaffold000006_2759336	1.38	1.77E-05	4.99		
*IbRCo_12*	*Immature rind color, b parameter*	12	27.5	9.3–11.9	CP32_scaffold000074_497940-CP32_scaffold000074_795378	0.91	5.23E-03	2.58		
*IaRCo_10*	*Immature rind color, a parameter*	10	104.9	5.9–11.8	CP32_scaffold000029_1132025-CP32_scaffold000029_1388824	1.05	3.64E-08	9.66		
*IaRCo_3*	*Immature rind color, a parameter*	3	86.1	20.3–27.8	CP32_scaffold000187_88187-CP32_scaffold000187_88366	1.17	5.86E-10	12.06	3.09E-02	7.17E-03
*MLRCo_4*	*Mature rind color, L parameter*	4	14.9	65.1–75.9	CP32_scaffold000066_82542-CP32_scaffold000143_78868	12.70	0.00E + 00	40.30	3.20E-02	
*MLRCo_1*	*Mature rind color, L parameter*	1	67.3	7.1–17.2	CP32_scaffold000010_400289-CP32_scaffold000175_201011	2.47	2.54E-02	1.60	1.50E-02	
*MLRCo_2*	*Mature rind color, L parameter*	2	13.2	13.4–16.2	CP32_scaffold000001_4952915-CP32_scaffold000001_5092215	3.55	1.31E-03	3.27	1.25E-02	4.84E-02
*MbRCo_4*	*Mature rind color, b parameter*	4	19.6	33.3–39.7	CP32_scaffold000180_195391-CP32_scaffold000143_294806	3.17	6.52E-11	12.84	1.18E-02	
*MbRCo_19*	*Mature rind color, b parameter*	19	52.1	6.9–12.4	CP32_scaffold000005_1580923-CP32_scaffold000005_2422557	2.63	1.49E-07	8.51	1.34E-02	
*MaRCo_4*	*Mature rind color, a parameter*	4	50.44	20.02–25.7	CP32_scaffold000022_598182-CP32_scaffold000022_1004798	2.08	1.33E-15	18.59		
*IbFCo_10*	*Immature flesh color, b parameter*	10	102.9	45.1–58.2	CP32_scaffold000029_1132025-CP32_scaffold000029_1802506	1.31	3.45E-12	15.02		
*IaFCo_10*	*Immature flesh color, a parameter*	10	100.4	12.1–15.4	CP32_scaffold000029_1280511-CP32_scaffold000029_1747554	1.05	0.00E + 00	28.31	2.24E-02	9.62E-04
*IaFCo_13*	*Immature flesh color, a parameter*	13	30.4	7.3–9.3	CP32_scaffold000113_399328-CP32_scaffold000049_351119	0.46	4.51E-05	5.42	4.02E-02	
*MbFCo_19*	*Mature flesh color, b parameter*	19	52.1	55.8–68.8	CP32_scaffold000005_1580923-CP32_scaffold000005_2422557	3.86	0.00E + 00	62.93		
*MaFCo_19*	*Mature flesh color, a parameter*	19	38.8	11.5–15.8	CP32_scaffold000005_2532610-CP32_scaffold000005_2907973	1.12	7.77E-16	18.86	5.64E-05	5.45E-03
*MaFCo_10*	*Mature flesh color, a parameter*	10	37.89	8.8–11.7	CP32_scaffold000009_1923620-CP32_scaffold000009_2317405	0.31	3.09E-02	1.49	3.63E-04	
*MaFCo_13*	*Mature flesh color, a parameter*	13	53.1	5.4–9.4	CP32_scaffold000017_2743863-CP32_scaffold000108_169208	0.41	5.71E-03	2.43	3.42E-04	

QTLs detected using data from the RIL population genotyped by GBS and phenotyped in three environments

### Vine-related traits

No significant QTLs were found for vine size and architecture despite RIL parents, Zucchini and Scallop, differed in growth habit (bushy *versus* intermediate), branching intensity (non-branched *versus* branched), and plant length and number of nodes (an average from 78 to 120 *versus* 210 to 277 cm, and from 5 to 70 *versus* 73 to 90 nodes, in the three environments for Zucchini and Scallop respectively).

Major QTLs were identified for leaf traits, such as leaf blade incision and the occurrence of silver leaf (*Li_10* and *Sl_12*) (Table 2 and Additional file 4), both traits related to photosynthesis rate. Zucchini plants developed deeply incised leaves whereas Scallop plants had weak incisions (Li scored as 4 *versus* 1 respectively in all environments). Large genetic correlations between locations where found for these two traits, r x, y ranging from 0.66 to 0.77 (Additional file 4) indicating that the norm of reaction for each genotype was similar in the three environments.

**Fig. 2:**
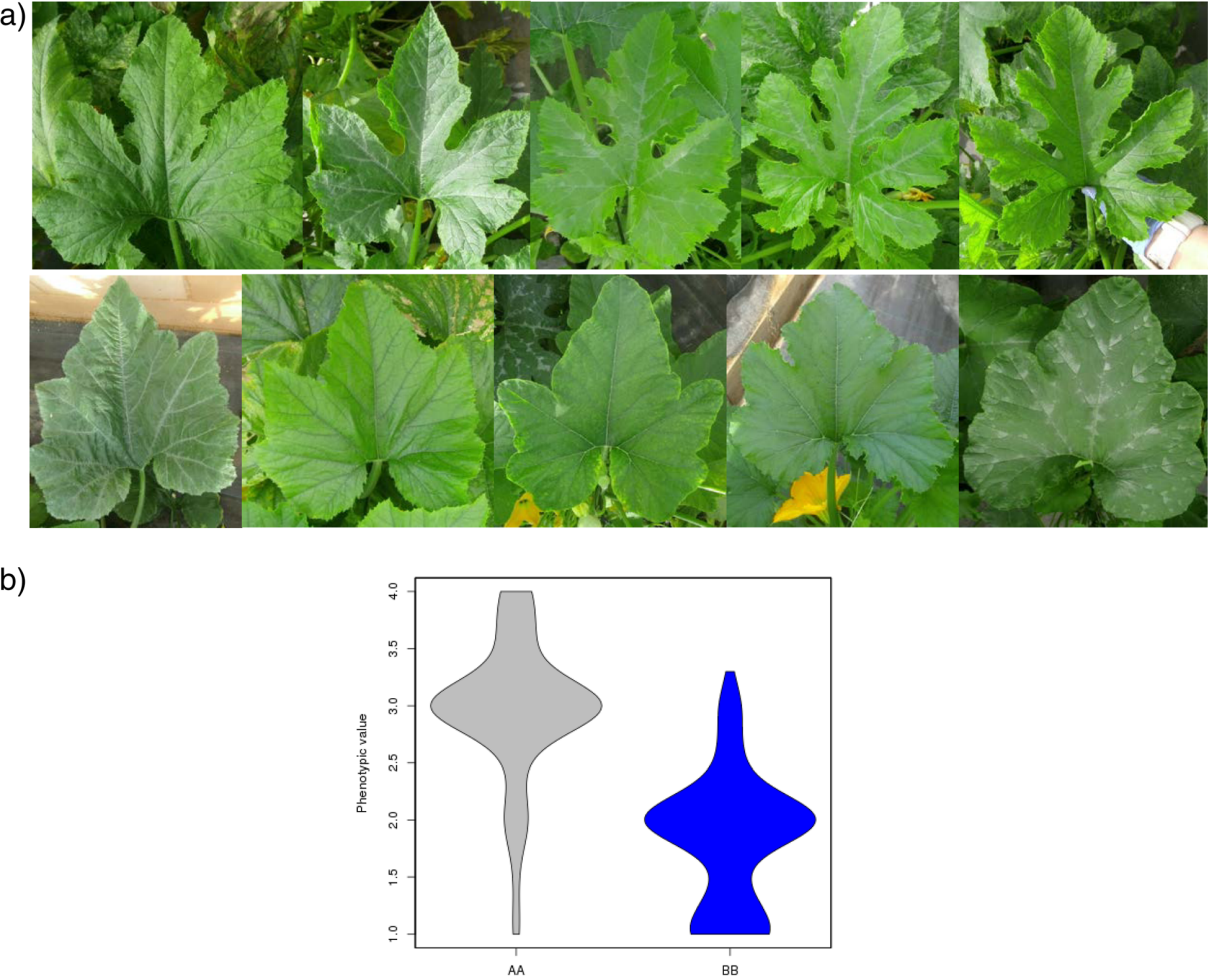
Effect of the QTL *Li_10*, controlling the intensity of leaf insertion. **a** Up: RILs with the Zucchini genotype and down RILs with the Scallop genotype; **b** Violin plot of the leaf insertion phenotypic values in the three assays for the AA (Zucchini) and the BB (Scallop) genotypes

*Li_10* (located at 33.9 cM, CP32_scaffold000009_2374010-CP32_scaffold000009_2556718) explained most of the variation found in this trait (R^2^ = 50.1%). ANOVA results show a lack of environment effect and of G x E interaction (Table 2), with RILs with Zucchini alleles having deeper incisions than RILs with Scallop alleles (Fig. 2) in all environments (average incision 2.9 *versus* 1.8) (Additional file 4 and 5). Two genes belonging to the homeobox-leucine zipper protein family were annotated in the *Li_10* region (CP32_scaffold000009-2401206-2403737 and 2409170-2410649) (Additional file 6). The best hit of these *Cucurbita* genes (against the non-redundant protein sequence database) was with homeobox-leucine zipper protein ATHB-22-like of *Cucumis melo*. This family of homeobox genes has roles in meristem identity and in the regulation of leaf development in several plant species [44, 45]. These could be good candidates to explain differences in leaf morphology found in this population.

The Zucchini and Scallop parents also differed in the occurrence leaf silvering. This is a typical feature of the Zucchini parental, whereas Scallop develops uniformly green leaves (Sl scored as 1 *versus* 0 respectively). Silver mottling of leaves is caused by the development of large air spaces between palisade cells and the epidermis. This trait has been reported to be controlled by a dominant single gene (*m* for non-mottled or non-silvery leaves and *M* for mottled or silvery leaves), with modifiers [46–48]. Also non-genetic factors, such as variation in light and temperature and drought stress, affect the expression of the silvery-leaf trait. Squash leaf silvering can also appear as a systemic response to whitefly feeding [48]. One major QTL, explaining 23.3% of the observed variation, was identified in our population in LG 12, *Sl_12* (59.3 cM, CP32_scaffold000031_1726079-CP32_scaffold000031_2012785) (Table 2 and Additional file 4). RILs with the Zucchini alleles in that region show different degrees of silvering, whereas those with the Scallop genotype develop green leaves in all the environments (Additional file 5, Fig. 3) (mean scores 0.49 *versus* 0.11). In fact no significant environment or G x E effect was found for this trait (Table 2, Additional file 4). Other two QTLs had LOD values above the threshold in the analysis with all the data (*p* = 0.01), *Sl_1* (132.5 cM, CP32_scaffold000084_728121-CP32_scaffold000059_548298) and *Sl_16* (66.1 cM, CP32_scaffold000034_1033951-CP32_scaffold000028_1777958) (Table 2, Additional file 4). However, these explained a low percentage of the observed variation (3.8 and 8.0%, respectively), and were not significant in all the assayed environments, showing a significant G x E interaction (Additional files 4 and 5).

**Fig. 3:**
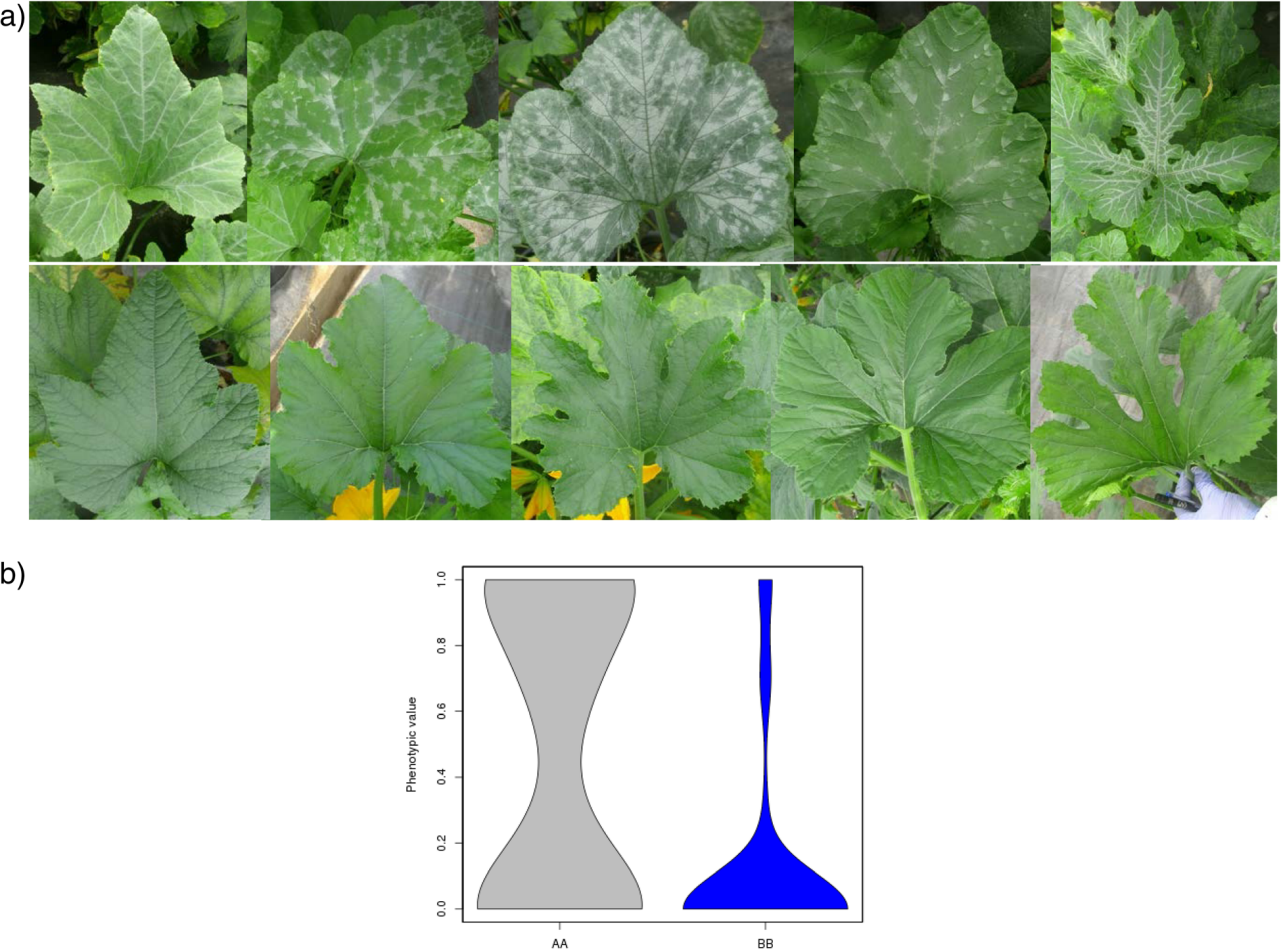
Effect of the QTL *Sl_12*, controlling the occurrence of leaf silvering. **a** Up: RILs with the Zucchini genotype and down RILs with the Scallop genotype. **b** Violin plot of the Silver leaf phenotypic values in the three assays for the AA (Zucchini) and the BB (Scallop) genotypes

Silvered leaves exhibit reduced photosynthetic ability, but this trait also seems to have a favorable effect on protection against aphids or plant desiccation, as silvery leaves reflect more light than non- silvery leaves [49]. A further study of the genes annotated in the corresponding QTL intervals (Additional file 6) is of interest to identify candidates involved in the variation of this trait and to manage it in breeding programs.

### Flowering time related traits

Cucurbits have become model species for the study of plant sex determination and some genes involved in sex expression (dioecy, monoecy, andromonoecy and gynoecy) have been characterized in melon, cucumber and squash [14, 50–55]. Most of them are enzymes involved in ethylene biosynthesis, signaling and regulation. The *Cucurbita* orthologs of some of the cloned genes (CmACS11, CmACS7, CsACS2, CSACS1g and CmWIP1 of melon and cucumber) are located in LG 13, LG 14, LG 18, LG 10, and LG 17 (CP32_ scaffold000017_63557, CP32_scaffold000011_561409; CP32_scaffold00025_1079779; CP32_ scaffold000009_993755; CP32_ scaffold000004_4527055). Other genes, such as CTR1 and CTR2, that confer reduced ethylene sensitivity and have been reported to be involved in the male/female ratio in *C. pepo* [56] are located in LG 3 and LG 6 (CP32_ scaffold000038_689227 and CP32_ scaffold000008_2476160). No significant QTLs involved in the variation of the flowering traits analyzed in this study colocalize with any of these regions (Additional file 4). This result is not unexpected as the Zucchini and Scallop accessions do not differ in sex expression, being both monoecious. In fact, sex expression is less variable in *Cucurbita* than in *Cucumis* crops, cucumber and melon.

Early flowering, however, is a highly variable and economically important agronomic trait related to early yield in *C. pepo*. It is affected by genetic, environmental and hormonal factors [57, 58]. Zucchini and Scallop do differ in flowering time, mainly in the days to the development of the first male and female flower, being the Scallop parental more late-flowering than Zucchini squash (average DMaF 18 to 23.5 *versus* 21 to 24.5 days and DFeF 18 to 30 *versus* 31.5 to 42 for Zucchini and Scallop in the three environments, respectively). Genetic correlations between locations for this trait were positive and significant, although moderate (r x, y > 0.28) (Additional file 4). The analysis of the RIL population suggests the existence of at least two genomic regions controlling flowering time. We found a QTL (R^2^ = 7.9%) involved in the earliness of female flowers in LG 12 (*DFeF_12*, 33.1 cM, CP32_scaffold000074_621895-CP32_scaffold000096_181501) (Table 2, Additional file 4). ANOVA results also indicate a significant effect of the environment and a lack of G X E interaction in this *locus* (Table 2). The RILs homozygous for the Zucchini alleles developed the first female flower in all environments between 3 and 8 days before than those homozygous for the Scallop allele (average DFeF 34.2 *versus* 39.7 days) (Additional file 5). One additional minor QTL involved in the variation of the same trait was found in LG 9 (R^2^ < 5%), *DFeF_9* (116 cM, CP32_scaffold000013_960688-CP32_scaffold000013_1308585) (Table 2 and Additional file 4), with significant E and G x E effects. Significant differences between the RILs with the Zucchini *versus* Scallop genotypes in this region were found in two environments, with an advance of the female flowering from 2 to 6 days (average DFeF 34.9 *versus* 37.4 days) (Additional file 5).

Some candidate genes are found in these regions (Additional file 6), opening new possibilities for the study of the genetics of this poorly studied trait. For example, two MADS-box genes are annotated in the *DFeF_12* region (CP32_ scaffold000074_189252-192320 and 193342-199503) that are most similar to the *Momordica charantia* AGAMOUS LIKE6-like protein (AG6) and to the MADS-box protein SOC1-like from *C. sativus*. The AGL6 gene acts as a floral promoter in *Arabidopsis* through the control of the transcription of key regulators of flowering time (the FLOWERING LOCUS C (FLC), the main flowering switch gene in *Arabidopsis*, and the FLOWERING LOCUS T (FT)). The overexpression of this gene results in a late-flowering phenotype [59]. Also the overexpression of SOC1-like stimulate flowering in different species [60]. In this region is also located a TCP transcription factor (CP32_ scaffold000074_159157-160182, best nr hit transcription factor TCP9 of *C. melo*). The TCP gene family plays important roles in regulating diverse processes, including phytohormone biosynthesis and signal transduction, branching and flower development [61]. A gene of this family was also annotated in the distorted region of LG 1 described above (Additional file 3c).

Variation in flowering time has been less studied than sex expression in cucurbits and little is known about the underlying genetic mechanism. In a recent study conducted in cucumber, a candidate gene for early flowering was identified (Csa1G651710), which is a homolog of the *Arabidopsis* LOCUS T [62]. Our results suggest that the regulation of the FT gene is also a main mechanism underlying the variation of the flowering time in *C. pepo*. The *C. pepo* ortholog of the Csa1G651710 is located in the scaffold150 (CP32_scaffold000150_132214-134774) that could not be mapped to any of the *C. pepo* linkage groups. This scaffold showed a high degree of distorted segregation towards the Zucchini alleles (Additional file 3c), which is consistent with the idea that Scallop alleles in this *locus* have resulted in late flowering affecting the reproduction of the carrier RILs during the selfing process. It remains to be studied if the presence of Zucchini regions in *DFeF_12* results in a change of FT expression associated to the early flowering genotypes.

Additionally, three genes are located in the region of the *DFeF_9*, that can be associated to the flowering process, one annotated as WUSCHEL-related homeobox (WOX) (CP32_ scaffold000013_1016547-1018778, Best hit WUSCHEL-related homeobox of *Cucumis melo*) and a second gene as an auxin response factor 4 (CP32_ scaffold000013_1041004-1045906). WOX genes are a large group of transcription factors essential in maintaining shoot apical meristem, some of which play important roles in the regulation of floral patterning. Some of these processes are conducted through the regulation of auxin transport [63, 64]. The third candidate in this region is an ethylene-responsive transcription factor (ERF4) (CP32_scaffold000013_1092413-1092817) as flowering is also associated to ethylene metabolism. Further research is necessary to determine whether flowering time traits co-segregate with variation in these genes.

None of these QTLs colocalize with the major QTL in LG 3 controlling several flowering traits (days to flowering, node to the first flower, etc.) detected using the F_2_ population of the same cross and the previous map by Esteras et al. [23]. Former LG 3 corresponds to LG 18 and LG 20 in the current map (Fig. 1). The SNPs flanking the flowering QTL in the F_2_ map (C006328, C001057 and C003831) were located in CP32_scaffold000027 (507871 and 2031514) and CP32_scaffold000025 (1811879), the first and second ones anchored in one end of the LG 20 and the last in another end of the LG 18. The CP32_scaffold000027 presents a high percentage of distorted markers (Additional file 3c), thus as described before we were not able to merge this two LG into one. Therefore, likely the lack of markers in this region is the reason why this main QTL detected in the F_2_ was not detected in the RILs population. In this region are two ETHYLENE INSENSITIVE3 (EIN3) genes and one ethylene-responsive transcription factor (ERF) annotated (CP32_scaffold000025-1811503 and 2037348; CP32_scaffold000027-1697493-1702248) encoding transcription factors that represent downstream components of ethylene signaling, also reported to be involved in flowering [65].

### Fruit morphology

Squash fruit morphology is related to different traits: fruit weight, fruit shape, fruit length and width, number of locules, and ribbing intensity. Zucchini and Scallop immature and mature fruits did not significantly differ in fruit weight, but they did differ in fruit shape (IFSh and MFSh scored as 6, elongated, *versus* 1, discoidal, respectively). Fruit shape differences were mainly due to differences in fruit length (IFLe from 16.7 to 23.7 cm *versus* 4.7 to 7.5 cm, and MFLe from 37.8 to 38.7 cm *versus* 6.9 to 9.8 cm, respectively for Zucchini and Scallop in the three environments).

Genetic correlations between locations were very high for some traits related to immature and mature fruit shape (r x,y ranging from 0.65 to 0.89 for IFSh, IFLe, MFSh, MFLe), and moderate for some others (r x, y ranging from 0.33 to 0.65 for IFWi, IFRib, MFWi, and MFRib) (Additional file 4).

A major QTL affecting immature fruit shape, *IFSh_3* (R^2^ = 17.8%, 173.2 cM, CP32_scaffold000038_1385297-CP32_scaffold000038_1853138) colocalized in LG 3 with major QTLs affecting immature fruit length and width (*IFLe_3 and IFWi_3*) (R^2^ = 31.8 and 18.7%, 173.2 and 171.1 cM, respectively) (Table 2, Additional file 4). Zucchini alleles in this region resulted in more elongated immature fruits, significantly longer and narrower than fruits with Scallop alleles (average IFLe 14.5 *versus* 10.3 cm, IFWi 5.8 *versus* 7.1 cm for homozygous Zucchini and Scallop respectively) (Additional file 5, Fig. 4a). Differences were significant in the three environments. The effect of the environment was significant for IFLe and IFLWi, whereas no significant G x E interaction was found in any of the traits (Table 2, Additional file 4). These shape differences were also appreciated in mature fruits, affecting more to fruit length than to width (MFLe 24.9 *versus* 16.0 cm and MFWi 10.1 *versus* 11.6 cm) (Additional file 5, Fig. 4b). In fact, QTLs involved in the mature fruits shape, length and width colocalized in the same region, *MFSh_3, MFLe_3* and *MFWi_3* (R^2^ = 11.0, 38.7 and 15.1%, 171.0, 169.1, and 171.0 cM) (Table 2, Additional file 4). No effect was observed in this region for fruit weight, number of locules, and other traits associated to fruit morphology, such as ribbing intensity. This region is the same found in the previous map (developed with the F_2_ population of the same cross) located in LG 6 [23], controlling the length of immature and mature fruits, and associated fruit shape traits (mature fruit width and cavity thickness). The comparison between the F_2_ and the RIL genetic map indicates the correspondence between LG 6 (F_2_) and LG 3 (RIL) (Fig. 1). The genetic basis of variation in fruit shape has been studied most extensively in tomato and in other cucurbits such as melon. For example in melon, some QTLs associated to fruit shape colocalize with members of the OVATE family proteins (OFP). Genes of this family are also involved in tomato fruit morphology [66–68]. The anchorage of the RIL map to the genome sequence provide the list of genes annotated in the *IFSh_3, IFLe_3, IFWi_3, MFSh_3, MFLe_3* and *MFWi_3* region (Additional file 6). Interestingly, this list includes an OFP gene (CP32_scaffold000038-1785881-1786918), the ortholog of the OFP2-like gene of *Cucumis sativus*, which likely contributes to the observed variation in *C. pepo* fruit shape.

**Fig. 4:**
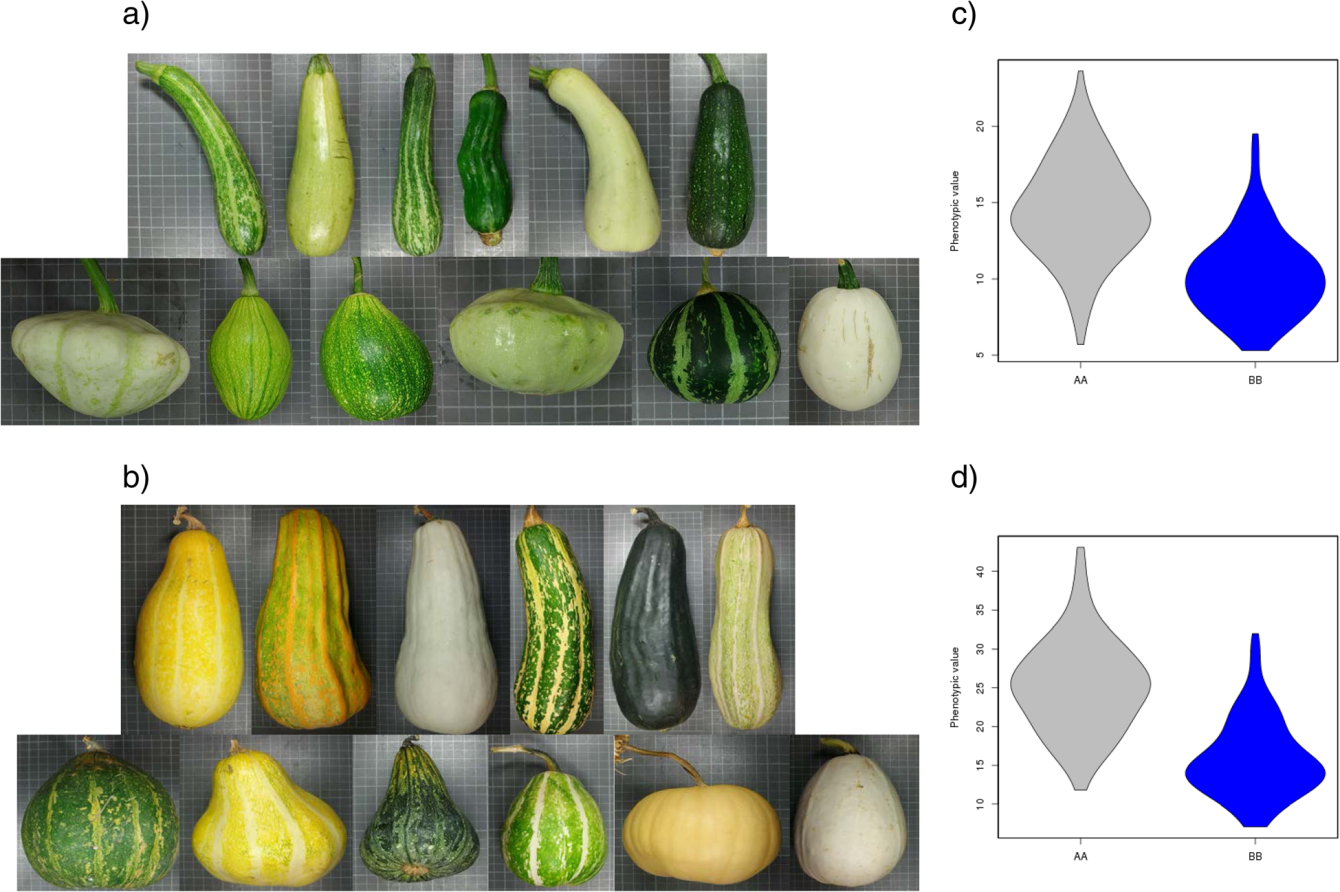
Effect of QTLs controlling fruit shape. **a** Effect of the QTL *IFLe_3* and *IFSh3*, controlling immature fruit shape. Up: RILs with the Zucchini genotype and down RILs with the Scallop genotype. **b** Effect of the QTL *MFLe_3* and *MFSh_3*, controlling mature fruit shape. Up: RILs with the Zucchini genotype and down RILs with the Scallop genotype. **c** and **d** Violin plots of the Immature and Mature Fruit Length phenotypic values in the three assays for the AA (Zucchini) and the BB (Scallop) genotypes

Two additional minor QTLs affecting immature fruit shape and length, *IFSh_12* (R^2^ = 6.7%, 25.0 cM, CP32_scaffold000065_760668-CP32_scaffold000074_729362) that colocalized in LG 12 with minor QTLs affecting immature and mature fruit length and width (*IFLe_12, MFLe_12* and *MFWi_12*) (R^2^ = 7.6, 6.6 and 7.4%, 24.2, 19.3 and 24.9 cM), and *IFLe_15* (R^2^ = 6.7%, 34.7 cM, CP32_scaffold000056_543412-CP32_scaffold000056_920404) were identified (Table 2, Additional file 4). In all cases Zucchini alleles increased fruit length, but the effects of these QTLs were much lower than that of QTLs in LG 3 and significant differences were found only in some environments (Additional file 5).

Also four additional regions in LG 4 (39.4 cM, CP32_scaffold000159_92504-CP32_scaffold000022_375710), LG 5 (27.9 cM, CP32_scaffold000018_362002-CP32_scaffold000018_549964), LG 6 (19.7 cM, CP32_scaffold000003_3022364-CP32_scaffold000003_3087204), and LG 9 (119.3 cM, CP32_scaffold000013_890110-CP32_scaffold000013_959962) affected mature fruit shape and length, *MFSh_4, MFSh_5, MFLe_6 and MFLe_9* (R^2^ = 3.4, 3.0, 3.6 and 6.2%) (Table 2, Additional file 4). *MFLe_9* was the only QTL in which the Scallop alleles increased fruit length (average MFLe 19.1 *versus* 22.7 cm for Zucchini and Scallop respectively) (Additional file 5). In these four QTLs the effect was only observed in mature fruits, suggesting that different regions affect fruit shape in the early and late steps of fruit development. Several genes had been previously reported to be related to fruit shape in *C. pepo* [48]. A dominant gene (*Di*) was reported associated to the discoidal fruit shape of scallop squash. This gene was suggested to be dominant over spherical or pyriform shapes. A digenic epistatic control has also been reported for summer squash fruit shape. Our results are consistent with the existence of a major gene, that could be the ovate underlying the *IFLe_3* QTL, and several minor modifiers.

**Fig. 5:**
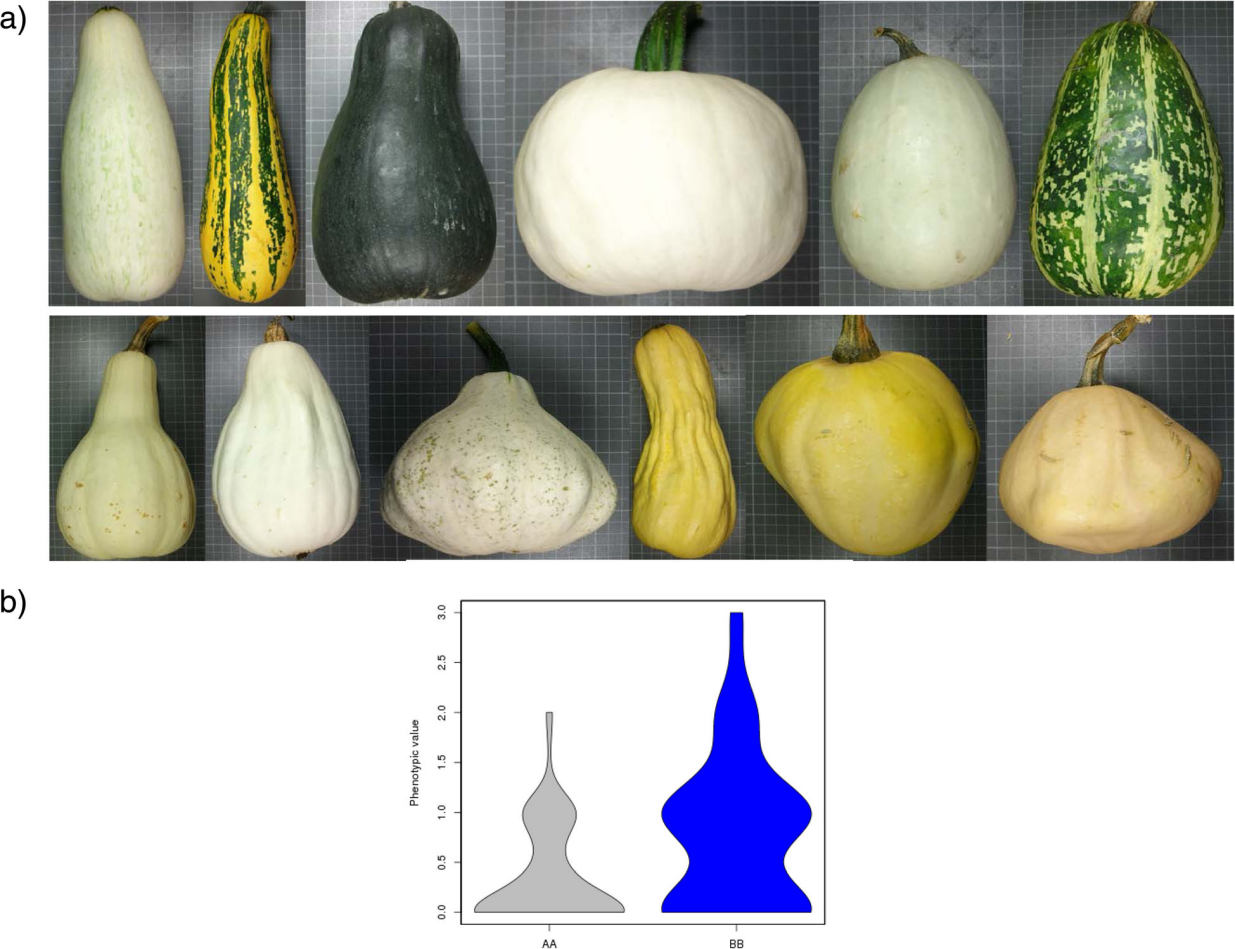
Effect of the *MFRib_12*, controlling the occurrence of ribbing in mature fruits. **a** Up: RILs with the Zucchini genotype and down RILs with the Scallop genotype; **b** Violin plot of the Mature Ribbing phenotypic values in the three assays for the AA (Zucchini) and the BB (Scallop) genotypes

Apart from fruit length and width, there are other traits associated to fruit morphology. The Scallop fruit is strongly scalloped with deeper ribs than Zucchini (IFRib 0 to 1 *versus* 2 to 3 and MFRi 0 to 0.5 *versus* 3, for Zucchini and Scallop respectively). The ribbing intensity was variable in the RIL population. Three QTLs involved in this trait were detected, one controlling ribbing intensity in immature fruits, *IFRib_3* (R^2^ = 8.7%, 69.4 cM, CP32_scaffold000006_3196015-CP32_scaffold000006_3616125) and two in mature fruits, *MFRib_12* (R^2^ = 10.7% 110.2 cM, CP32_scaffold000031_115435-CP32_scaffold000045_1342921), and *MFRib_21* (R^2^ = 6.2% 19.2 cM, CP32_scaffold000007_4220403-CP32_scaffold000110_136233) (Table 2, Additional file 4). Zucchini alleles in the *IFRib_3* region resulted in more ribbed fruits, although not in all environments (1.5 *versus* 0.85 in Zucchini and Scallop genotype, respectively). In fact, both environment and G x E effects were significant for *IFRib_3*, whereas in *MFRib_12* and *MFRib_21* resulted in less ribbed fruits (0.34 *versus* 0.79 and 0.46 *versus* 0.83) in all environments, with no significant G x E effects. *MFRib_12,* that explained the highest percentage of the variation found in this trait (Fig. 5), had been previously detected in the map of the F_2_ population (MRib_11 in LG 11 of the F_2_ map that correspond to LG 12 of the RIL map) (Fig. 1). Transcription factors belonging to the WOX family have been reported to control the carpel number [69]. We did not find genes belonging to this family in the reported regions, so other genes must be underlying these QTLs (Additional file 6).

Peduncle length is also involved in fruit typology. Significant differences were found among parents in the three environments, with shorter peduncles in the Zucchini fruits (IPeLe 2–3 *versus* 4.3–5.3 cm and MPeLe 1.4–3.6 *versus* 5.2–9.5 cm). Genetic correlations between assays were moderate for peduncle traits (Additional file 4). Three QTLs were identified controlling immature and mature fruit peduncle length (Table 2 and 3, Additional file 4): *IPeLe_10* (R^2^ = 7.6%, 71.3 cM, CP32_scaffold000009_272548-CP32_scaffold000009_704495), *IPeLe_16* (R^2^ = 3.7% 14.9 cM, CP32_scaffold000030_1135277-CP32_scaffold000030_1337312) and *MPeLe_14* (R^2^ = 3.9%, 29.3 cM, CP32_scaffold000039_1662180-CP32_scaffold000011_1038061). These QTLs have opposite effects of the Zucchini *versus* Scallop alleles (IPeLe 4.2 *versus* 5.5 and 5.3 *versus* 4.4 for *IPeLe_10 and 16*, respectively and 4.4 *versus* 5.3 for *MFLe_14*) (Additional file 5).

### Fruit color

Apart from fruit shape, other factors involved in fruit quality have been studied: organoleptic (flesh sugar content, Brix, and acidity, pH), nutritional (several measures of rind and flesh color), and physical (flesh firmness). Zucchini and Scallop parents did not differ in flesh firmness, Brix degree and pH, but clearly differed in fruit color, both of rind and flesh. Color was measured with colorimeter (taking the three parameters of the Hunter scale, L, Lightness, from white, L = 100, to black, L = 0; a, from redness for positive values to greenness for negative values; b, from yellowness for positive values to blueness for negative values). The Zucchini parent develops immature dark green fruits (characterized by low ILRCo values, 24.4 to 28.3, negative IaRCo, −2.6 to −6.8, and, positive IbRCo, 4.3 to 4.6, scores) with light green flesh (characterized by high ILFCo values, 52.9 to 81.3, negative IaFCo, −4.3 to −6.8, and high IbFCo values, 11.1 to 23.8) in the three environments. Immature Scallop fruits have light green rinds (with significantly higher values of ILRCo, 64.7 to 77.3 and IbRCo, 17.7 to 19.1, but similar values of the a parameter) and light green flesh, similar to that of the Zucchini fruits (ILFCo 79.5 to 82.4, IaFCo −4.1 –6.6, and IbFCo 13.9–15.5). Color differences were more significant in mature fruits. Zucchini fruits were dark green (MLRCo 21.2 to 25.6, MaRCo −1.6 to −0.48 and MbRCo 1.2 to 6.0), and the flesh varied from light green to light yellow or orange at physiological maturity (MLFCo 49.8 to 76.6, MaFCo −1.6 to 0.8 and MbFCo 12.9 to 24.4). However, the Scallop fruits remained white, both rind and flesh, at full maturity (with significantly higher rind and flesh lightness, MLRCo 78.8 to 81.9 and MFLCo 78.7 to 80.4, and rind yellowness values, MbRCo 11.4 to 12.5, and significantly lower flesh redness and yellowness, MaFCo −1.3 to −0.62 and MbFCo 9.7 to 13.3). Genetic correlations between environment were very high for the L trait (r x, y >0.75) and from moderate to high for the a and b color parameters (r x, y > 0.45) measured both in fruit rinds and flesh (Additional file 4).

**Fig. 6:**
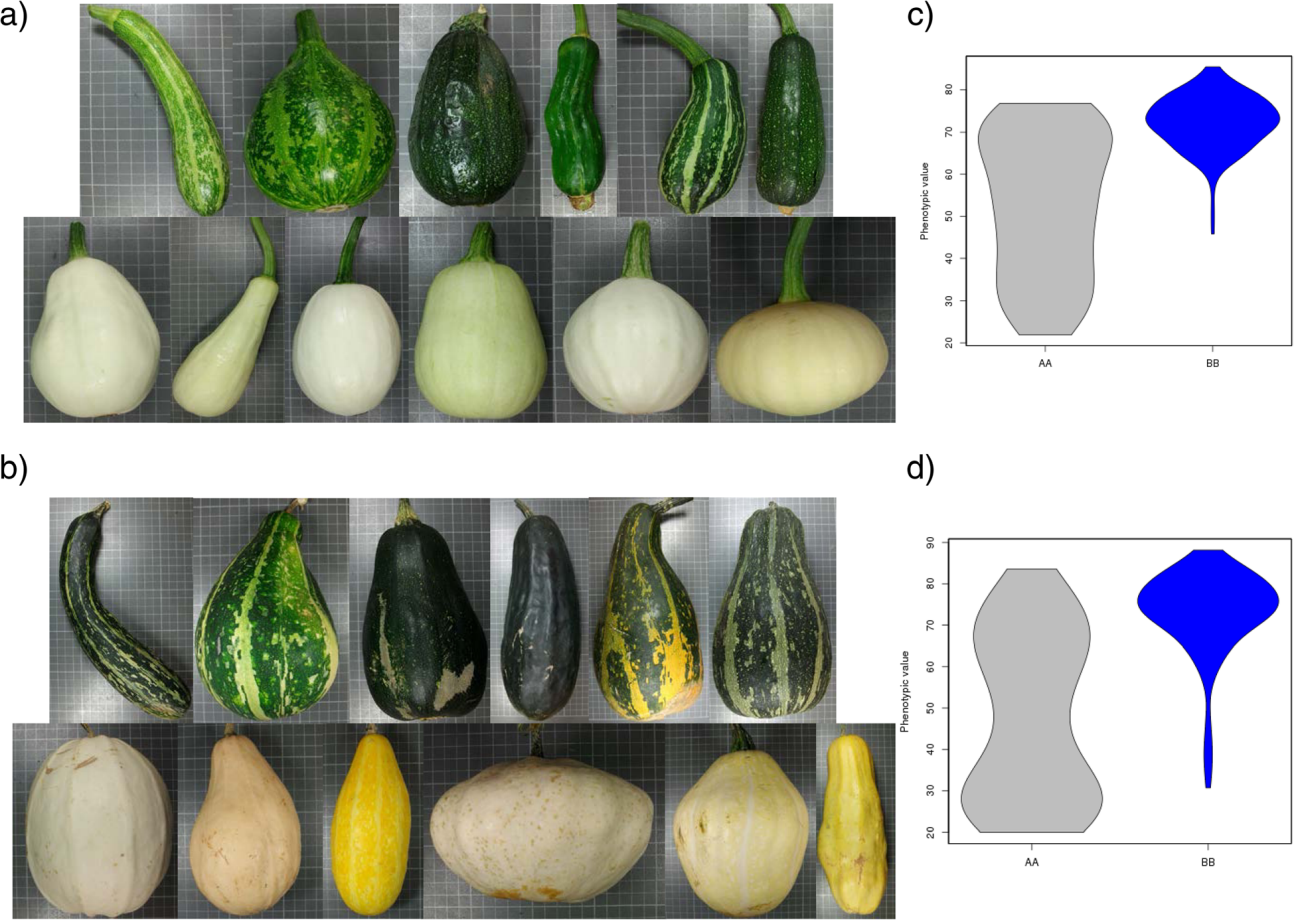
Effect of QTLs controlling fruit rind color. **a** Effect of the QTL *ILRCo_4*, controlling immature fruit rind color. Up: RILs with the Zucchini genotype and down RILs with the Scallop genotype; **b** Effect of the QTL *MLRCo_4*, controlling mature fruit rind color. Up: RILs with the Zucchini genotype and down RILs with the Scallop genotype. **c** and **d** Violin plots of the Immature/Mature Fruit Rind Color (Lightness parameter) in the three assays for the AA (Zucchini) and the BB (Scallop) genotypes

We found a major region controlling the rind color of the immature and mature fruit in LG 4. The major QTLs *ILRCo_4* and *IbRCo_4* (R^2^ = 40.6 and 15.4%, 14.9 and 15.3 cM, CP32_scaffold000066_82542-CP32_scaffold000143_130468), explaining most of the variability found in immature rind color, colocalized with two major QTLs explaining most of the variation found in mature rind color, *MLRCo_4* and *MbRCo_4* (R^2^ = 40.3 and 12.8%, 14.9 and 19.6 cM, CP32_scaffold000066_82542 -CP32_scaffold000143_294806) (Table 2, Additional file 4). These QTLs control the occurrence of rind dark green color. In fact, the fruits from RILs with Zucchini alleles develop immature/mature fruits with darker green primary color, with or without stripped or mottle secondary color pattern, and with low values of L and b parameters (average values in the three environments ILRCo 52.6 *versus* 72.4, IbRCo 16.1 *versus* 20.5 and MLRCo 47.8 *versus* 73.1, MbRCo 14.9 *versus* 21.2 for RILs with Zucchini versus Scallop alleles) (Additional file 5) (Fig. 6a and b). These major QTLs colocalize with the major QTL for the rind color of mature fruits mapped previously in LG 14 with the F_2_ population (*MLRCo_14* and *MaRCo_14*) [23] that correspond to LG 4 in the RIL map (Fig. 1).

In the LOD peak region of these QTLs, two genes annotated as related to an *Arabidopsis* APRR2-Like (ARABIDOPSIS PSEUDO RESPONSE REGULATOR2-LIKE gene) (CP32_ scaffold000180_115300-127146 and 130920-135450) (Additional file 6) were found. Genes of this family have been demonstrated to act as fruit-related regulators of pigment accumulation in tomato and pepper [70]. In fact the presence of a stop codon mutation explain color differences between the wild-type and a white-fruited pepper cultivar associated to differences in chlorophyll content. Our results also suggest a similar function of these genes in the Cucurbitaceae family.

Apart from the main effect of *ILRCo_4-IbRCo_4/MLRCo-4MbRCo_4*, immature rind color seems to be controlled by additional genomic regions. The main ones were in LG 10 and LG 3. The first region affected also flesh color, *ILRCo_10, IaRCo_10, IaFCo_10* and *IbFCo_10* (R^2^ = 6.0, 9.7, 28.3 and 15.0%, 99.5, 104.9, 100.4 and 102.9 cM, CP32_scaffold000029_1132025-CP32_scaffold000029_1802506). RILs with Scallop/Zucchini genotype had immature fruits with different greenish and yellowish tones in rind and flesh (ILRCo 67.9 *versus* 60.5 and IaRCo −8.3 *versus −*10.4, IaFCo −6.2 *versus −*4.1 and IbFCo 16.6 *versus* 13.9 for Zucchini and Scallop, respectively) (Additional file 5). A similar effect was found in LG 3 (*IaRCo_3*, R^2^ = 12.1%, 86.1 cM CP32_scaffold000187_88187-CP32_scaffold000187_88366), but significant environment and G x E effect were found in this region (Table 2, Additional file 4). Other less important regions (R^2^ < 5%) involved in the variation of immature rind color were *ILRCo_1, IbRCo_3* and *IbRCo_12*. These results are consistent with the traditionally proposed genetic control for rind color in squash, with a major gene derived from the Scallop genotype, *W* (weak rind coloration), complemented by modifiers, which confers a white or cream solid color independently of the genetic background. This gene has been reported to be epistatic to *D* (Dark stem) derived from Zucchini squash that result in dark stem and dark intermediate-age fruit [48]. The APRR2-like genes underlying *ILRCo_4-IbRCo_4/MLRCo-4MbRCo_4* are good candidates to be the *W* previously described squash gene.

The rind color of mature fruits was more variable than the immature fruit color, as during the ripening process yellow and orange colors develop in some fruits (Fig. 6b). Two QTLs were found involved in the redness (a parameter) variation (*MaRCo_4* 50.4 cM, CP32_scaffold000022_ 598182-CP32_scaffold000022_1004798) and in the yellowness (b parameter) variation (*MbRCo_19*, 52.1 cM, CP32_scaffold000005_1580923-CP32_scaffold000005_2422557). The major QTL *MaRCo_4* explained most of the variation found in MaRCo (R^2^ = 18.6%), with the Zucchini alleles resulting in fruits with more orange color in rinds in all environments (MaRCo 0,88 *versus −*2,6 for Zucchini and Scallop genotypes) (Additional file 5). The QTL *MbRCo_19* (R^2^ = 8.6%) resulted in fruits with rind color variable for the yellowness trait (MbRCo 22.2 *versus* 16.9 for Zucchini and Scallop genotypes). *MbRCo_19* colocalized with the major QTL explaining variation in mature flesh color discussed below. Two additional minor (R^2^ < 5%) QTLs affecting rind lightness were detected in LG 1 and 2, *MLRCo_1* and *MLRCo_2* with Zucchini genotypes having darker fruits than Scallops, but with a significant environment and G x E interaction (Additional file 5).

The genetic control of external fruit color has been investigated in melons. Recently, Feder et al. [71] identified a Kelch domain-containing F-box protein regulating naringenin chalcone accumulation in melon rind producing the change from white to yellow rind. This gene (MELO3C011980, annotated as similar to F-box/kelch-repeat protein At1g23390) colocalizes with QTLs involved in the variation of external color in melons [72, 73]. In *C. pepo*, we found two genes annotated as F-box/kelch-repeat protein (Additional file 6) in the LOD peak regions of the *MaRCo_4* QTL (CP32_ scaffold000022_600093-603383 and 624476-611084, best nr hit F-box/kelch-repeat protein At1g55270-like and At2g44130-like respectively). Also a Zeaxanthin epoxidase (ZEP) gene (CP32_ scaffold000022_750677-751766), an enzyme known to be involved in carotenoid metabolism [74], was found in that region. Also in the region of the QTL *MbRCo_19* is annotated a Phytoene synthase (PSY) enzyme (CP32_scaffold000005_2373613-2377294), known to be involved in the carotenoids biosynthesis (Additional file 6).

**Fig. 7:**
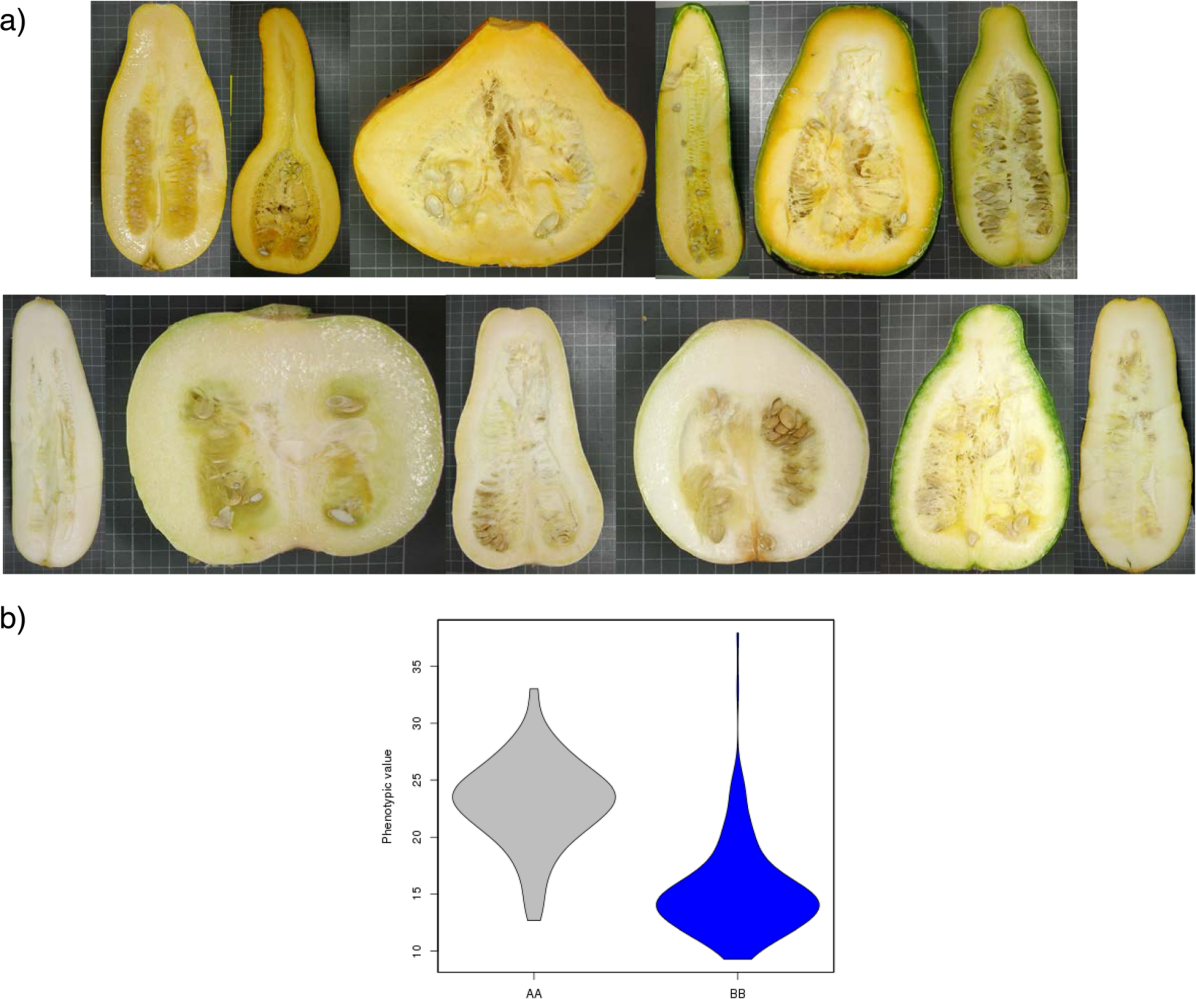
Effect of the QTL *MbFCo_19,* controlling mature fruit flesh color. **a** Up: Mature fruits of RILs with the Zucchini genotype and down RILs with the Scallop genotype; **b** Violin plot of the Mature Fruit Flesh Color (b parameter) in the three assays for the AA (Zucchini) and the BB (Scallop) genotypes

Our data suggest that flesh color in mature fruits is controlled by two major regions in LG 19. *MbFCo_19* (R^2^ = 62.9%) (52.1 cM, CP32_scaffold000005_1580923-CP32_scaffold000005_2422557) and *MaFCo_19* (R^2^ = 18.9%) (38.8 cM, CP32_scaffold000005_2532610-CP32_scaffold000005_2907973). The major QTL controlling flesh color, *MbFCo_19*, corresponds to the QTL for mature fruit flesh color found previously in the F_2_ map in LG 16 (*MbFCo_16*) (Fig. 1). Zucchini alleles in this regions, *MbFCo_19*, resulted in light orange fleshed fruits (Fig. 7), with higher b values than those found in white-fleshed fruits from RILs with Scallop alleles (MbFCo 24.1 *versus* 14.8). The effect of *MaFCo_19* (MaFCo −0.53 *versus −*2.77) was similar although less pronounced (Additional files 4 and 5), and significant E and G x E effects (Additional file 5). In melon, flesh color is controlled by two major genes: green flesh (*gf*) [75] and white flesh (*wf*) [76]. The gen *wf* has been recently isolated, corresponding to a close homolog of the Cauliflower OR (Orange) protein with the capacity of inducing β-carotene accumulation [77]. The *C. pepo* ortholog of this gene is located in CP32_scaffold00085-628461. The function of OR is to induce the differentiation of plastids into chromoplasts for carotenoid accumulation. This protein contains a Cysteine-rich zinc finger domain that is highly specific to DnaJ-like molecular chaperons. It is possible that OR works in association with a DnaJ-like protein to bind to proteins specific for the plastid differentiation/division. Underlying the QTLs for flesh color *MbFCo_19* and *MaFCo_19* there are several DnaJ-like proteins, along with several enzymes of the carotenoid biosynthesis pathway, such as one Phytoene synthase (PSY) (CP32_scaffold000005-2373613) and one Carotenoid cleavage dioxygenase (CCD) (CP32_scaffold000005-2201145) (Additional file 6). These genes are good candidates to be the previously described major gen *Wf* (white flesh), from Scallop, which is dominant over colored flesh [48].

Other two minor QTLs (R^2^ < 5%) involved in the variation of flesh color were also detected, although with significant E and G x E effect (Additional file 5) (*MaFCo_10*, 37.9 cM, CP32_scaffold000009_1923620-CP32_scaffold000009_2317405, and *MaFCo_13*, 53.1 cM, CP32_scaffold000017_2743863-CP32_scaffold000108_169208) that can act modulating the effect of the major gene, as it has been also reported in melons [72, 73, 78–80].

## Conclusions

A high-quality SNP marker collection has been developed for mapping and construction of the first saturated map in the species, with more than 7,000 markers and anchored to the current version of the *C. pepo* genome by 145 scaffolds. The improvement in the number of markers per LG and the extensive phenotyping of the RIL population, have enabled the detection of 48 QTLs, most of them stable across three environments.

The availability of the *C. pepo* genome annotation https://cucurbigene.upv.es [13] has facilitated the identification of candidate genes underlying most of these QTLs, which will allow the knowledge of the underlying processes that give rise to these phenotypic traits. We can highlight the identification of candidate genes underlying the variation of QTLs that explain more than 30% of the variation found in leaf incision, fruit shape, rind and flesh color, traits of evident economic importance, which can be exploited for searching new attractive market products and may also imply and increase of nutritional value.

## Additional files

Additional file 1: Phenotyped traits in the RIL population. (DOCX 13 kb)
Additional file 2: Average percentage of genome covered with the GBS reads with a read depth from > 1 to > 20. (PPTX 713 kb)
Additional file 3:
**a)** Genetic map developed using the GBS approach with the RIL population derived from the cross Zucchini x Scallop. The genetic and physical position of each of the the 7,718 SNP markers (in the *C. pepo* genome version 3.2) is indicated and the flanking sequenced of each SNPs is also included. **b)** Physical position and annotation (in the *C. pepo* genome version 3.2) of the SNPs that showed statistically significant distorted segregation. Chi-square *p*-values were corrected for multiple testing using Benjamini and Yekutieli correction [36]. Distorted SNPs are integrated with the 7,718 non-distorted SNPs used for the map construction for which the genetic position is also indicated. **c)** Main regions with distorted segregation (main genomic blocks with most of the SNPs with distorted segregation). (XLS 4656 kb)
Additional file 4:
**a)** LOD peaks of the QTLs identified in the RIL population for vine, flowering and fruit traits. Lines represent the thresholds p 0.01 and 0.05. Results of CIM analysis with a 20 cM windows are shown for each trait. First box show QTL results with data from all the environments and the other three boxes show the QTL results for single environments Paip2014, Paip2015 and UPV2015. **b)** Means and errors of the phenotype of alternative allelic classes, homozygous Zucchini (AA) and homozygous Scallop (BB), are shown for each QTL, calculated with the full set of data and with the data form each environment separately. **c)** Genetic correlations (r x,y) between locations for each trait (**p* < 0.05, ***p* < 0.01, ****p* < 0.001, ns non-significant). (PPTX 4972 kb)
Additional file 5: Phenotypic values of RILs carrying Zucchini and Scallop alleles in the detected QTL regions. Means and standard errors of the phenotypic value of RILs belonging to the alternative allelic classes (Zuchini *versus* Scallop) for the markers in the LOD peak regions of each significant QTL are shown. Data of the three environments, combined and separate, are shown to validate the effects of the different QTLs (Paip2014, Paip2015, and UPV2015). (DOCX 39 kb)
Additional file 6: List of genes underlying the significant QTLs involved in vine, flowering and fruit traits variation annotated in the last version of the *C. pepo* genome (v 3.2) available [13]. (XLSX 146 kb)

## Abbreviations

AFLP: Amplified fragment length polymorphisms
ANOVA: Analysis of variance
CCD: Carotenoid cleavage dioxygenase
CIM: Composite interval mapping
*D*: Dark stem (gene)
*Di*: Discoidal fruit (gene)
E: Environment
ERF: Ethylene-responsive transcription factor
FLC: Flowering locus C protein
FT: Flowering locus T protein
G: Genotype
GBS: Genotyping-by-sequencing
*gf*: Green flesh (gene)
LG: Linkage group
LOD: logarithm of odds
*M*: Mottled or silvery leaves (gene)
MAF: Minimum allele frequency
OFP: OVATE family protein
OR: Orange protein
PSY: Phytoene synthase
QTL: Quantitative trait loci
RAPD: Random amplified polymorphic DNA
RIL: Recombinant inbreed line
SNP: Singl nucleotide polymorphism
SSR: Simple sequence repeat
*W*: Weak rind coloration (gene)
*wf*: White flesh (gene)
ZEP: Zeaxanthin epoxidase
