# Supplementary material for: An SNP-based saturated genetic map and QTL analysis of fruit-related traits in Zucchini using Genotyping-by-sequencing
Source: BMC Genomics. 2017 Jan 18;18:94. doi: 10.1186/s12864-016-3439-y (PMC5241963; doi:10.1186/s12864-016-3439-y)
Supplement: Additional file 1: — Phenotyped traits in the RIL population. (DOCX 13 kb) [file 12864_2016_3439_MOESM1_ESM.docx]

Additional file 1. Phenotyped traits in the RIL population.

| **Trait code** | **Description/categories** |
| --- | --- |
| ***Vine traits*** | |
| **GH** | **Growth habit, 1 bushy, 2 intermediate, 3 prostrate** |
| **PLe** | **Plant length measured at the end of the assay (cm)** |
| **NoNº** | **Number of Nodes measured at the end of the assay** |
| **BI** | **Branching Intensity, 0 no branching, 1 branching with determinate lateral shoots, 2 intense branching with viny lateral shoots** |
| **SpPe** | **Spines in petioles, 0 absence, 0.5 intermediate, 1 abundant** |
| **Li** | **Leaf blade incision, 0 absent,1 week, 2 medium, 3 deep, 4 very deep** |
| **Sl** | **Silver mottling of foliage, 0 absent, 0.5 intermediate, 1 intense** |
| ***Flowering traits*** | |
| **NoMaF** | **First node with a male flower** |
| **NoFeF** | **First node with a female flower** |
| **DMaF** | **Days from transplanting to the development of the first male flower** |
| **DFeF** | **Days from transplanting to the development of the first female flower** |
| ***Immature/Mature fruit*** | |
| **IPeLe/MPeLe** | **Peduncle length (mm)** |
| **IFLe/MFLe** | **Fruit length (cm)** |
| **IFWi/MFWi** | **Fruit width (cm)** |
| **IFSh/MFSh** | **Fruit Shape, scored as 1 discoidal, 2 flattened, 3 globular, 4 oval, 5 oblong, 6 elongated** |
| **IFWe/MFWe** | **Fruit weight (g)** |
| **ILoN°/MLoNº** | **Number of locules** |
| **IFRib/MFRib** | **Intensity of fruit ribbing, scored visually based on presence and depth of the ribs as 0 absent, 1 surface ribbing, 2 intermediate ribbing and 3 strong ribbing** |
| **IBrix/MBrix** | **Soluble solids content I fruit flesh (Brix degree measured with refractometer)** |
| **IpH/MpH** | **pH of fruit flesh (measured with pH paper)** |
| **IFFi/MFFi** | **Flesh firmness measured with penetrometer (kg)** |
| **ILRCo/MLRCo** | **Rind color measured with colorimeter, Hunter parameter L (Lightness: from white, L = 100, to black, L = 0)** |
| **IaRCo/MaRCo** | **Rind color measured with colorimeter, Hunter parameter a (from redness for positive values to greenness for negative values)** |
| **IbRCo/MbRCo** | **Rind color measured with colorimeter, Hunter parameter b (from yellowness for positive values to blueness for negative values)** |
| **ILFCo/MLFCo** | **Flesh color measured with colorimeter, Hunter parameter L** |
| **IaFCo/MaFCo** | **Flesh color measured with colorimeter, Hunter parameter a** |
| **IbFCo/MbFCo** | **Flesh color measured with colorimeter, Hunter parameter b** |
