# Supplementary material for: An SNP-based saturated genetic map and QTL analysis of fruit-related traits in Zucchini using Genotyping-by-sequencing
Source: BMC Genomics. 2017 Jan 18;18:94. doi: 10.1186/s12864-016-3439-y (PMC5241963; doi:10.1186/s12864-016-3439-y)
Supplement: Additional file 2: — Average percentage of genome covered with the GBS reads with a read depth from > 1 to > 20. (PPTX 713 kb) [file 12864_2016_3439_MOESM2_ESM.pptx]

## Slide 1
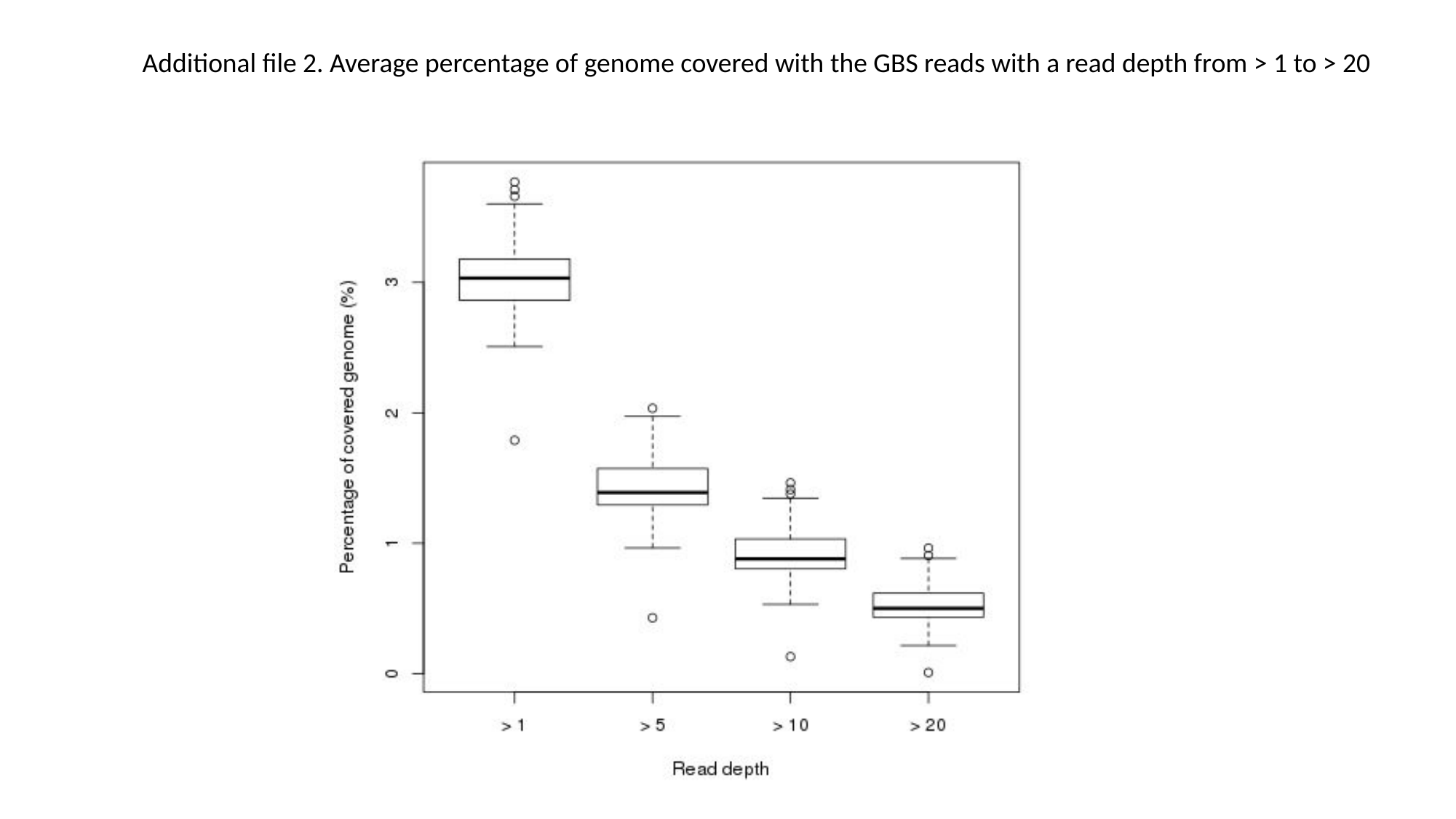

Additional file 2. Average percentage of genome covered with the GBS reads with a read depth from > 1 to > 20
