# Supplementary material for: An SNP-based saturated genetic map and QTL analysis of fruit-related traits in Zucchini using Genotyping-by-sequencing
Source: BMC Genomics. 2017 Jan 18;18:94. doi: 10.1186/s12864-016-3439-y (PMC5241963; doi:10.1186/s12864-016-3439-y)
Supplement: Additional file 4: — a) LOD peaks of the QTLs identified in the RIL population for vine, flowering and fruit traits. Lines represent the thresholds p 0.01 and 0.05. Results of CIM analysis with a 20 cM windows are shown for each trait. First box show QTL results with data from all the environments and the other three boxes show the QTL results for single environments Paip2014, Paip2015 and UPV2015. b) Means and errors of the phenotype of alternative allelic classes, homozygous Zucchini (AA) and homozygous Scallop (BB), are shown for each QTL, calculated with the full set of data and with the data form each environment separately. c) Genetic correlations (r x,y) between locations for each trait (*p < 0.05, **p < 0.01, ***p < 0.001, ns non-significant). (PPTX 4972 kb) [file 12864_2016_3439_MOESM4_ESM.pptx]

## Slide 1
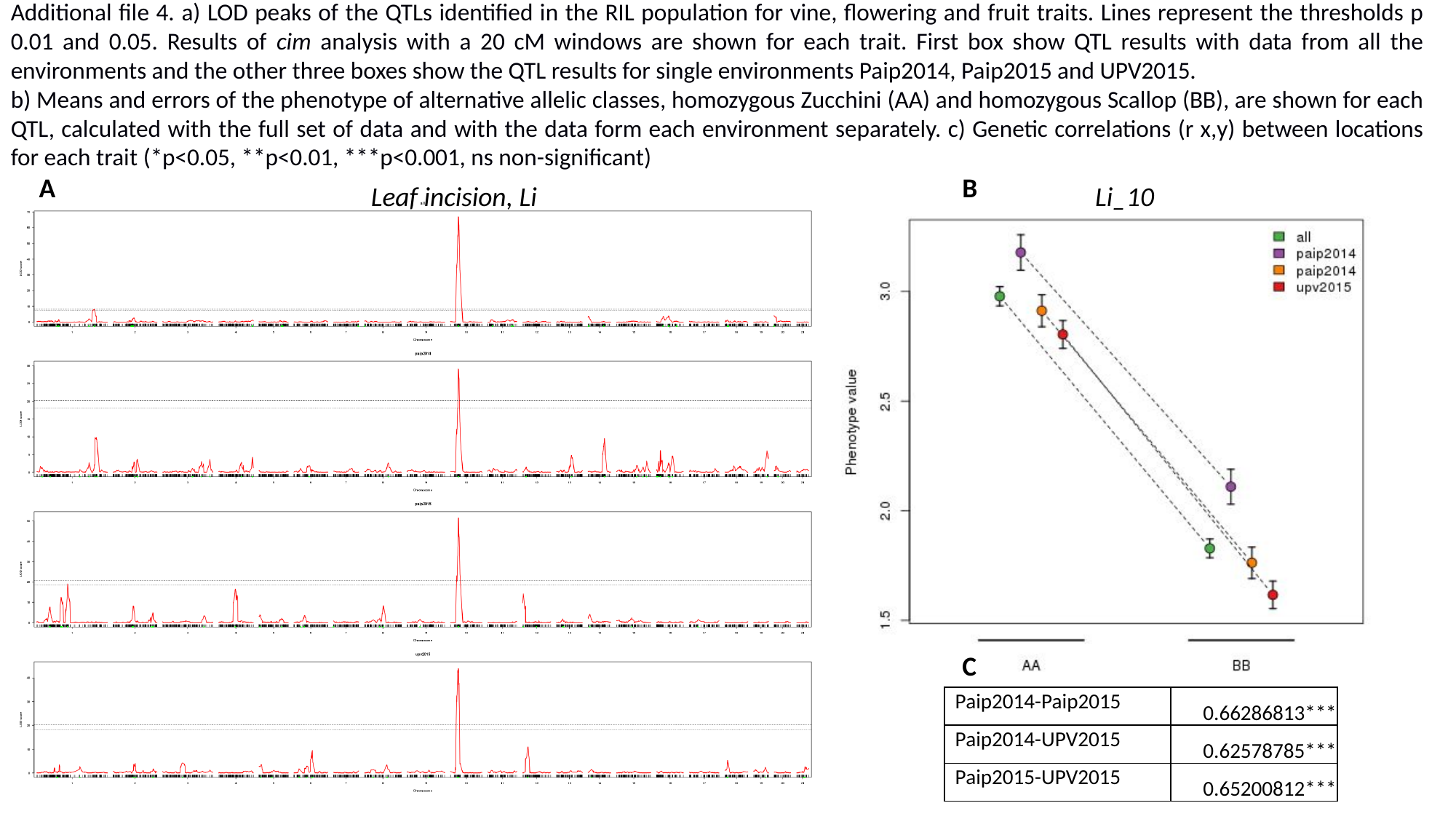

Additional file 4. a) LOD peaks of the QTLs identified in the RIL population for vine, flowering and fruit traits. Lines represent the thresholds p 0.01 and 0.05. Results of cim analysis with a 20 cM windows are shown for each trait. First box show QTL results with data from all the environments and the other three boxes show the QTL results for single environments Paip2014, Paip2015 and UPV2015.
b) Means and errors of the phenotype of alternative allelic classes, homozygous Zucchini (AA) and homozygous Scallop (BB), are shown for each QTL, calculated with the full set of data and with the data form each environment separately. c) Genetic correlations (r x,y) between locations for each trait (*p<0.05, **p<0.01, ***p<0.001, ns non-significant)
A
B
Leaf incision, Li
Li_10
C
| Paip2014-Paip2015 | 0.66286813\*\*\* |
| --- | --- |
| Paip2014-UPV2015 | 0.62578785\*\*\* |
| Paip2015-UPV2015 | 0.65200812\*\*\* |

## Slide 2
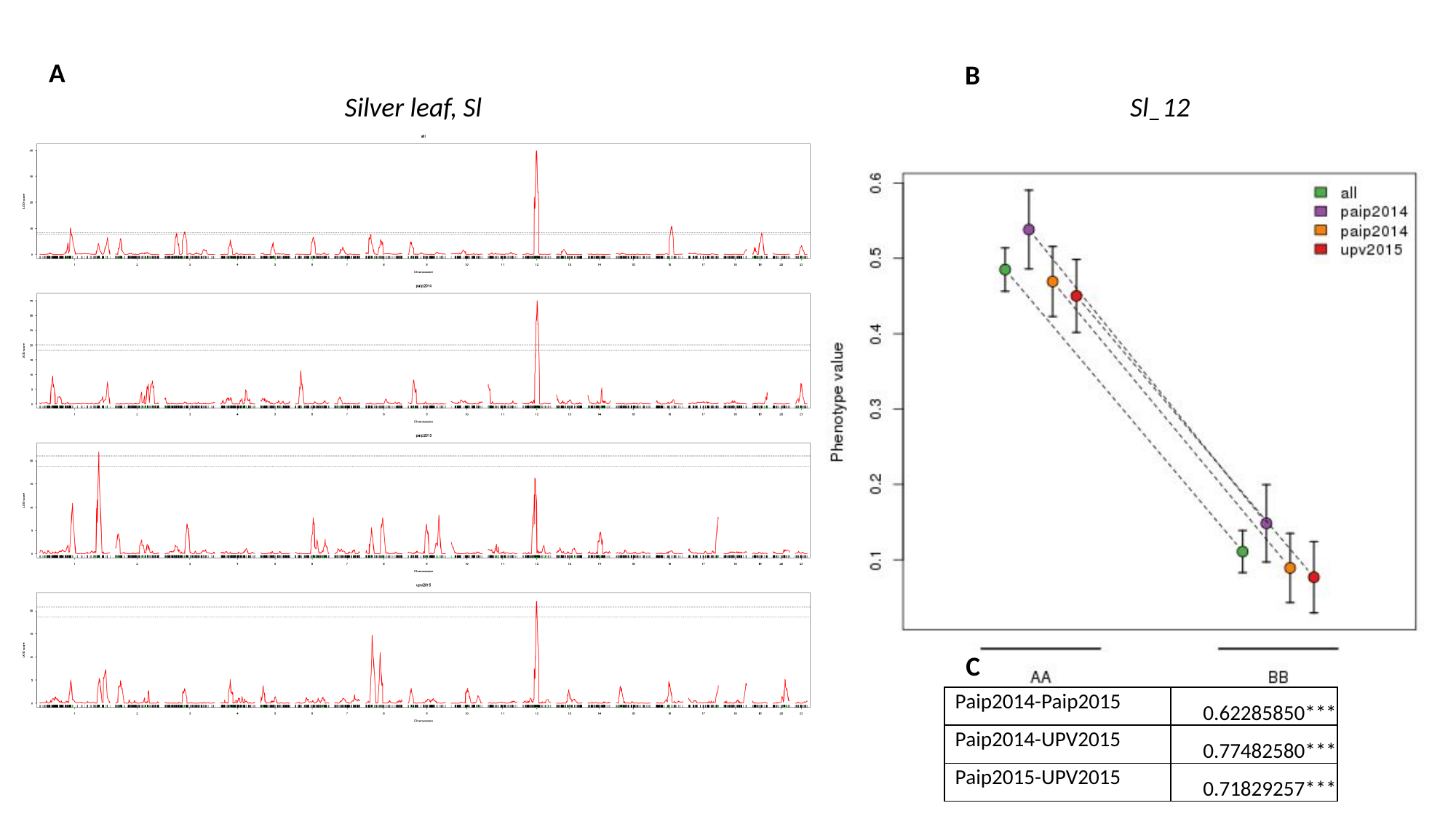

A
B
Silver leaf, Sl
Sl_12
C
| Paip2014-Paip2015 | 0.62285850\*\*\* |
| --- | --- |
| Paip2014-UPV2015 | 0.77482580\*\*\* |
| Paip2015-UPV2015 | 0.71829257\*\*\* |

## Slide 3
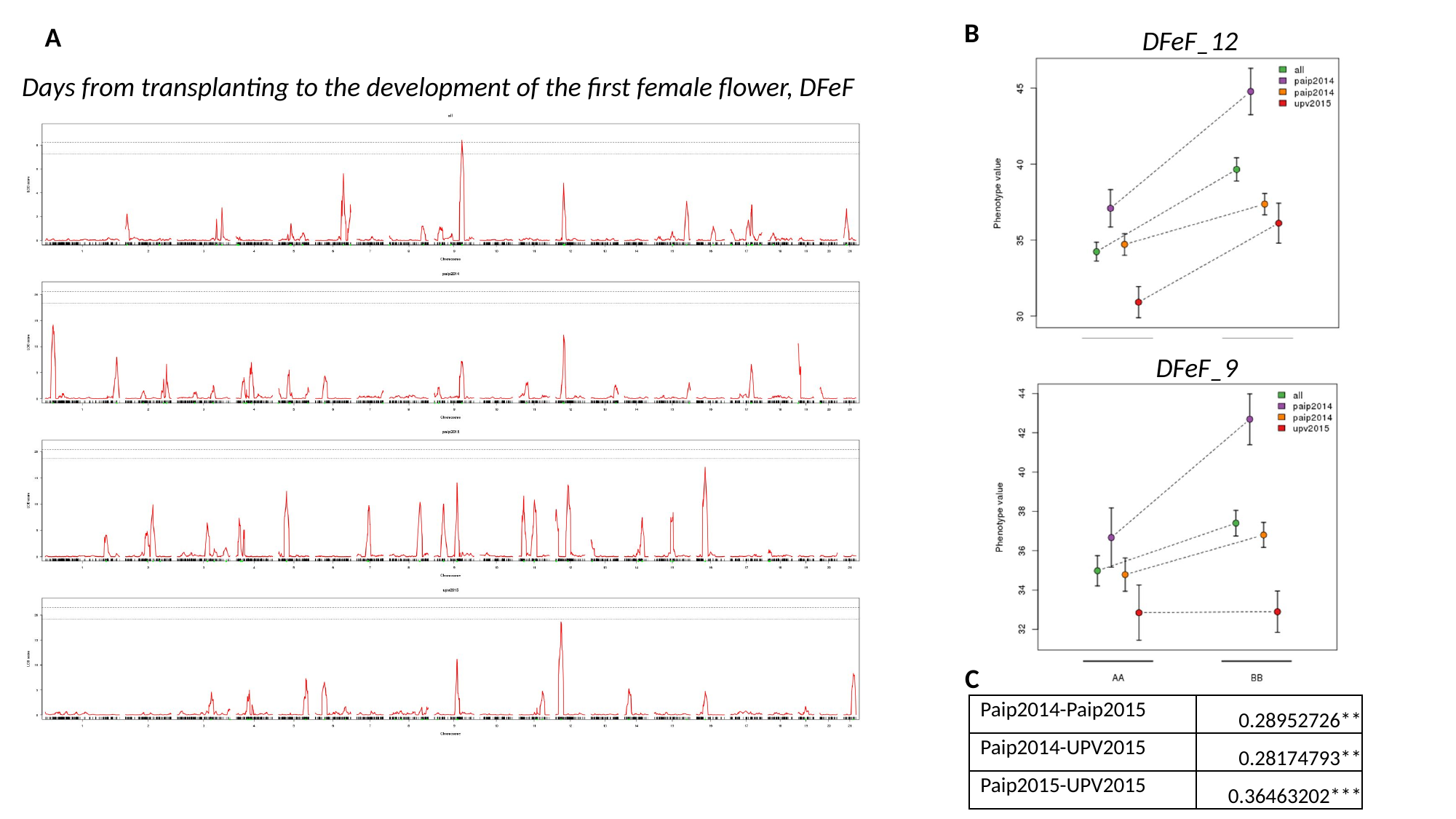

B
A
DFeF_12
Days from transplanting to the development of the first female flower, DFeF
DFeF_9
C
| Paip2014-Paip2015 | 0.28952726\*\* |
| --- | --- |
| Paip2014-UPV2015 | 0.28174793\*\* |
| Paip2015-UPV2015 | 0.36463202\*\*\* |

## Slide 4
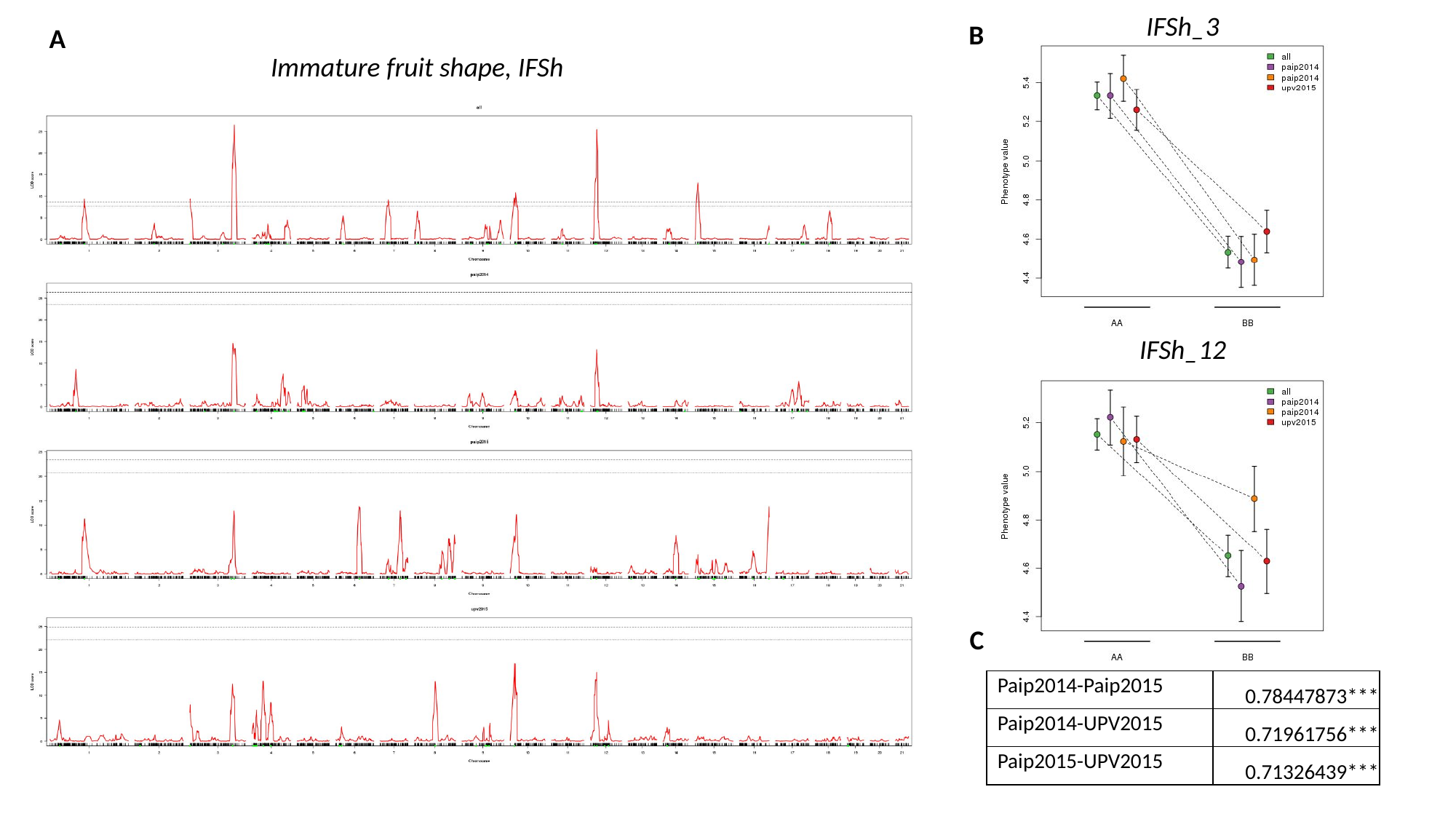

IFSh_3
B
A
Immature fruit shape, IFSh
IFSh_12
C
| Paip2014-Paip2015 | 0.78447873\*\*\* |
| --- | --- |
| Paip2014-UPV2015 | 0.71961756\*\*\* |
| Paip2015-UPV2015 | 0.71326439\*\*\* |

## Slide 5
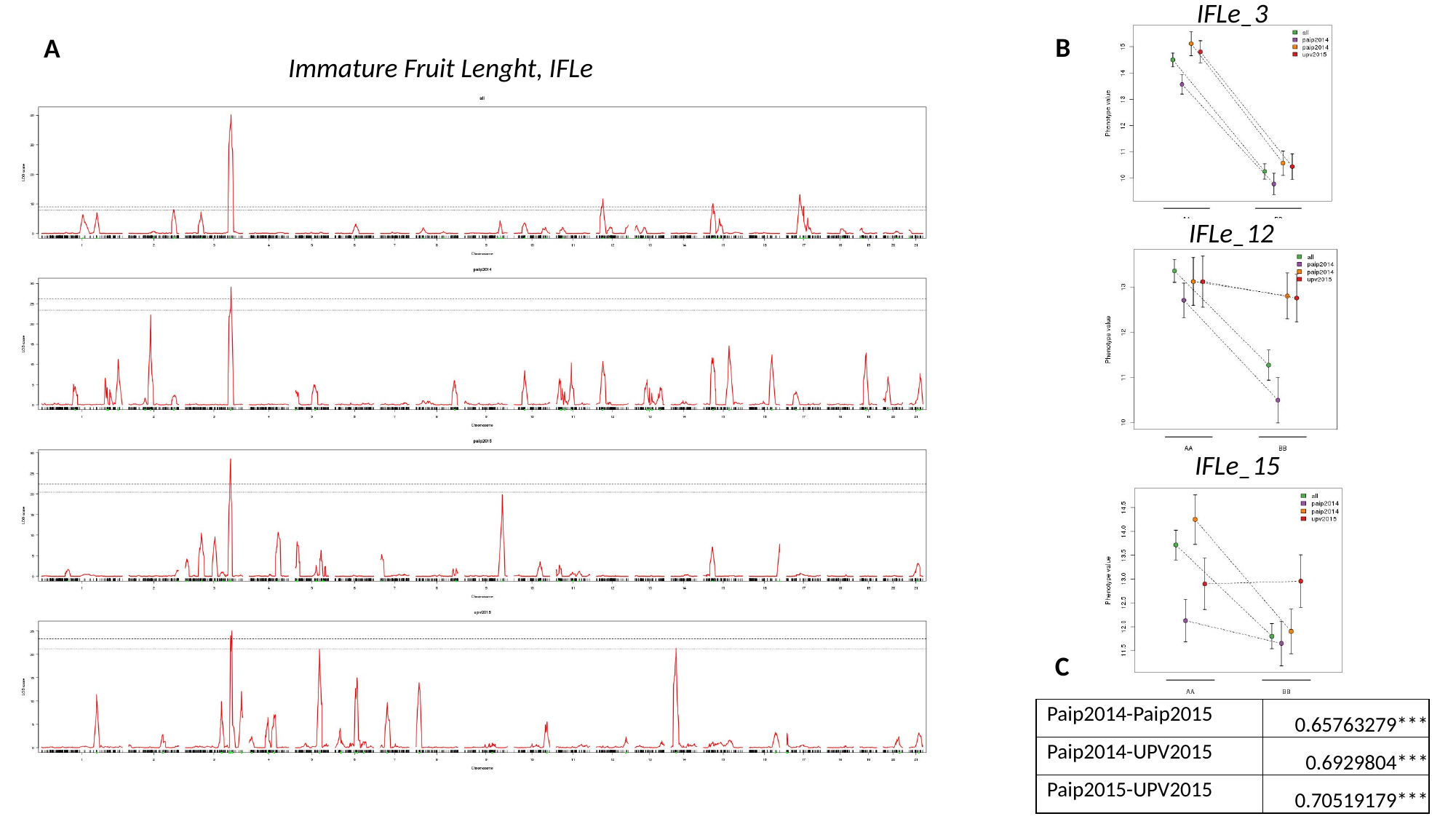

IFLe_3
B
A
Immature Fruit Lenght, IFLe
IFLe_12
IFLe_15
C
| Paip2014-Paip2015 | 0.65763279\*\*\* |
| --- | --- |
| Paip2014-UPV2015 | 0.6929804\*\*\* |
| Paip2015-UPV2015 | 0.70519179\*\*\* |

## Slide 6
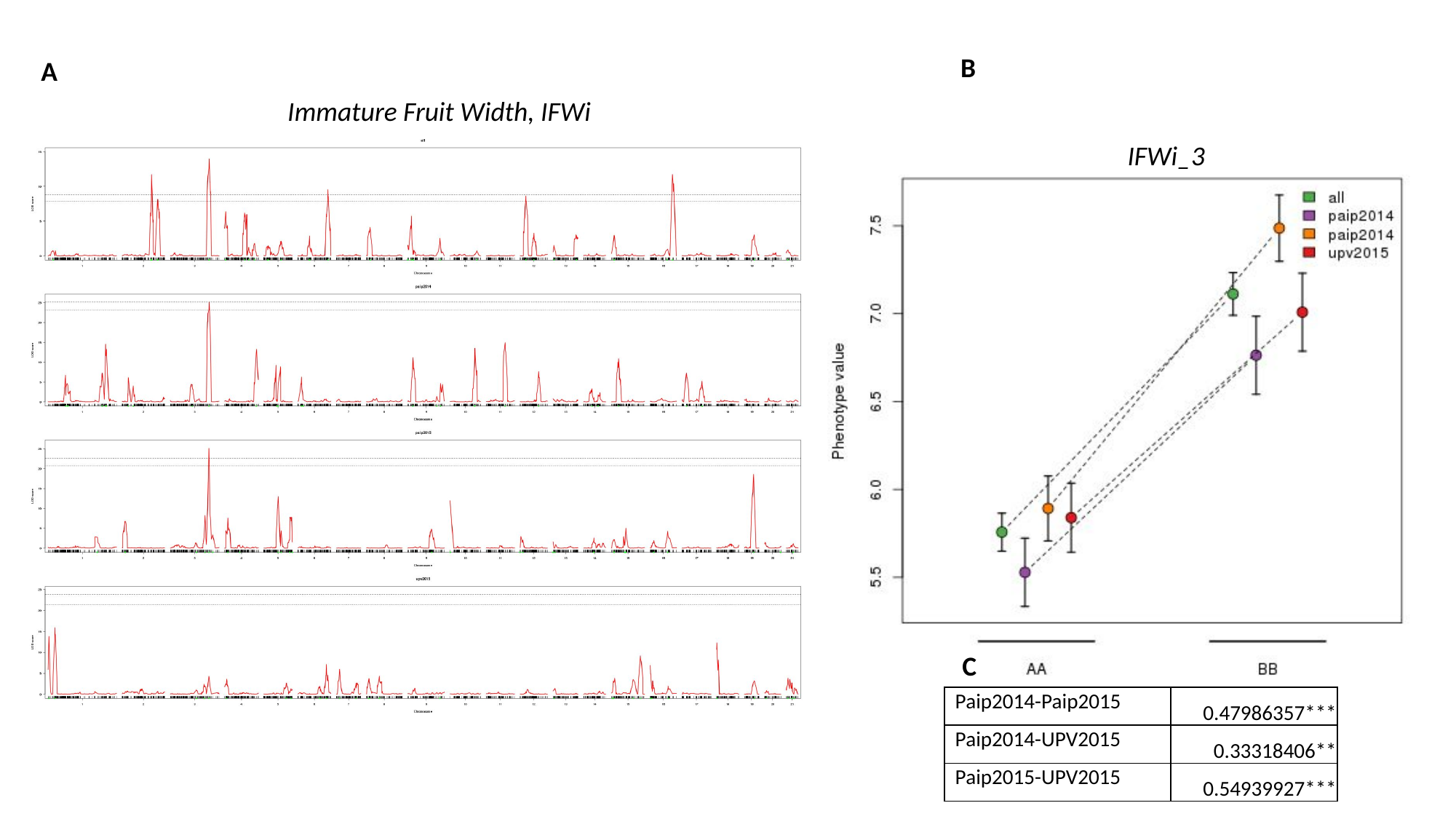

B
A
Immature Fruit Width, IFWi
IFWi_3
C
| Paip2014-Paip2015 | 0.47986357\*\*\* |
| --- | --- |
| Paip2014-UPV2015 | 0.33318406\*\* |
| Paip2015-UPV2015 | 0.54939927\*\*\* |

## Slide 7
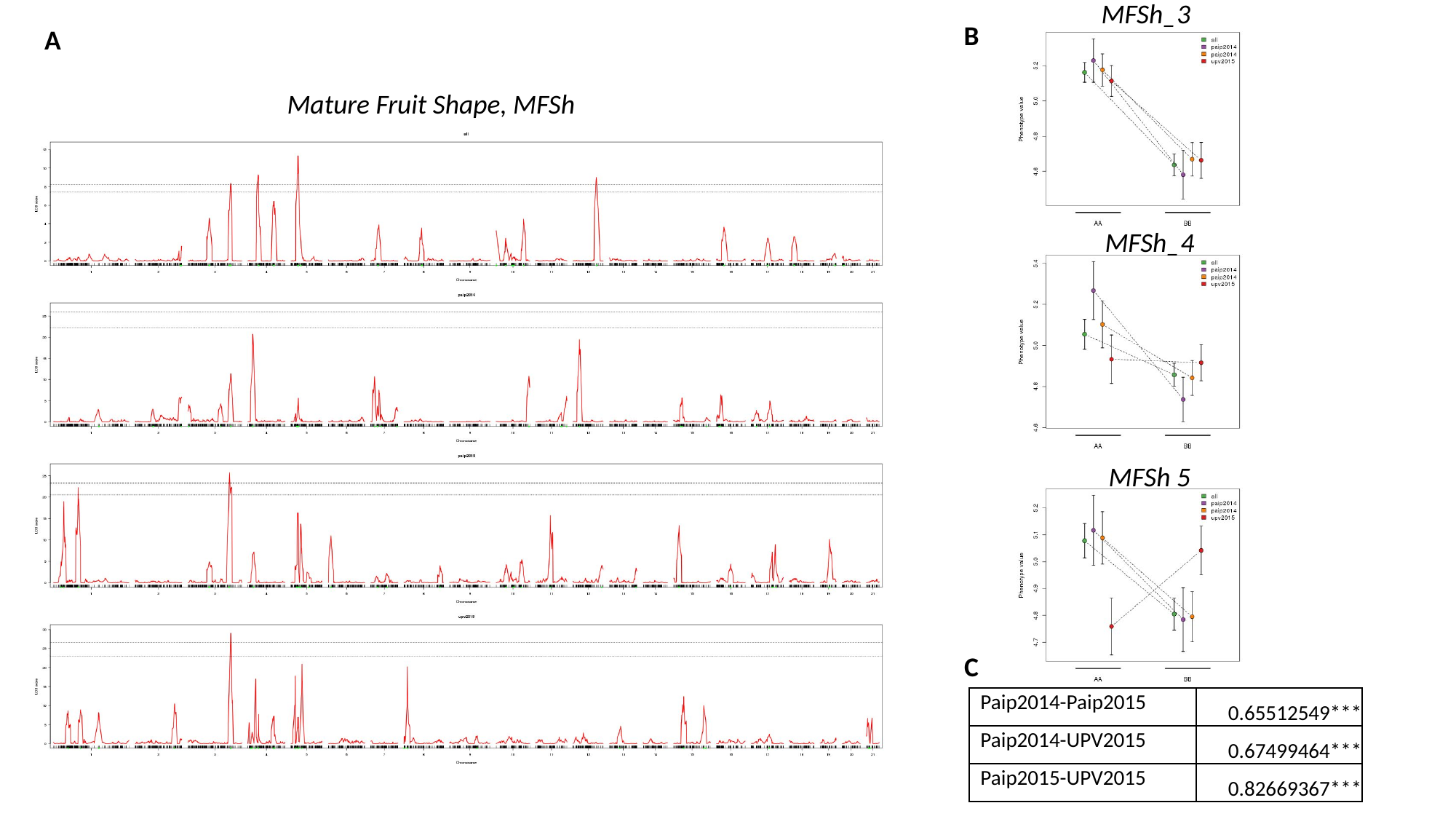

MFSh_3
B
A
Mature Fruit Shape, MFSh
MFSh_4
MFSh 5
C
| Paip2014-Paip2015 | 0.65512549\*\*\* |
| --- | --- |
| Paip2014-UPV2015 | 0.67499464\*\*\* |
| Paip2015-UPV2015 | 0.82669367\*\*\* |

## Slide 8
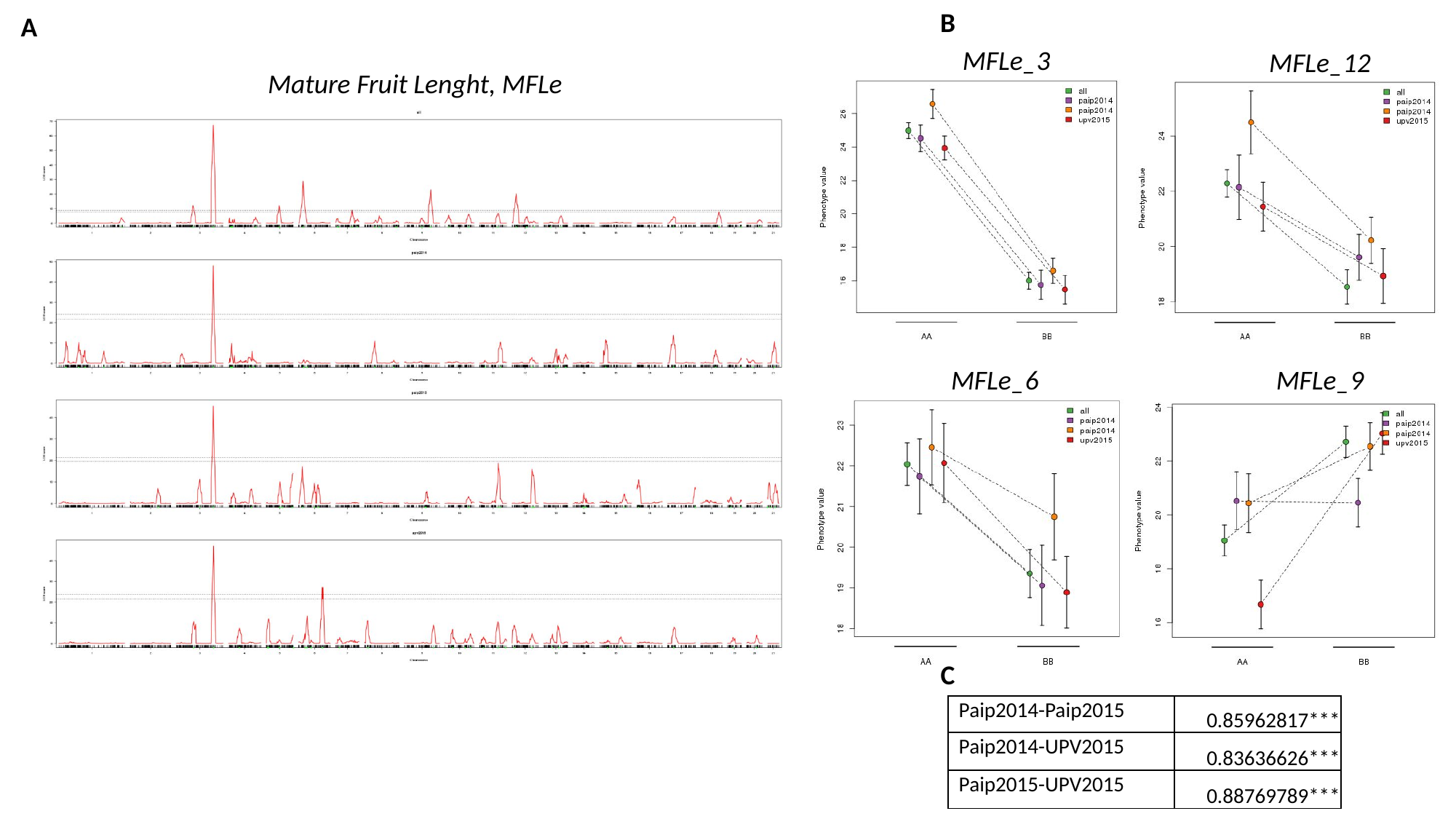

B
A
MFLe_3
MFLe_12
Mature Fruit Lenght, MFLe
MFLe_6
MFLe_9
C
| Paip2014-Paip2015 | 0.85962817\*\*\* |
| --- | --- |
| Paip2014-UPV2015 | 0.83636626\*\*\* |
| Paip2015-UPV2015 | 0.88769789\*\*\* |

## Slide 9
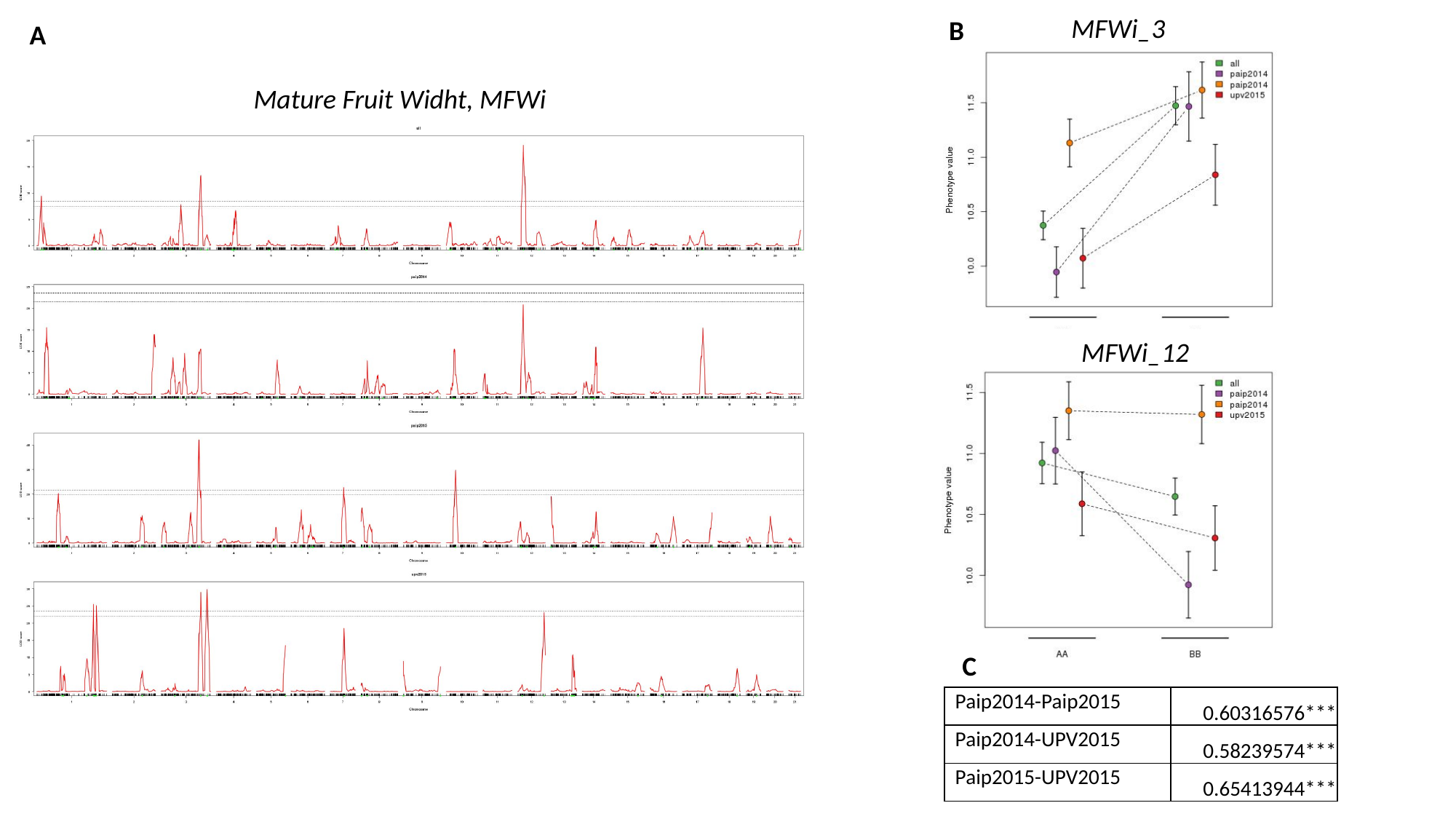

MFWi_3
B
A
Mature Fruit Widht, MFWi
MFWi_12
C
| Paip2014-Paip2015 | 0.60316576\*\*\* |
| --- | --- |
| Paip2014-UPV2015 | 0.58239574\*\*\* |
| Paip2015-UPV2015 | 0.65413944\*\*\* |

## Slide 10
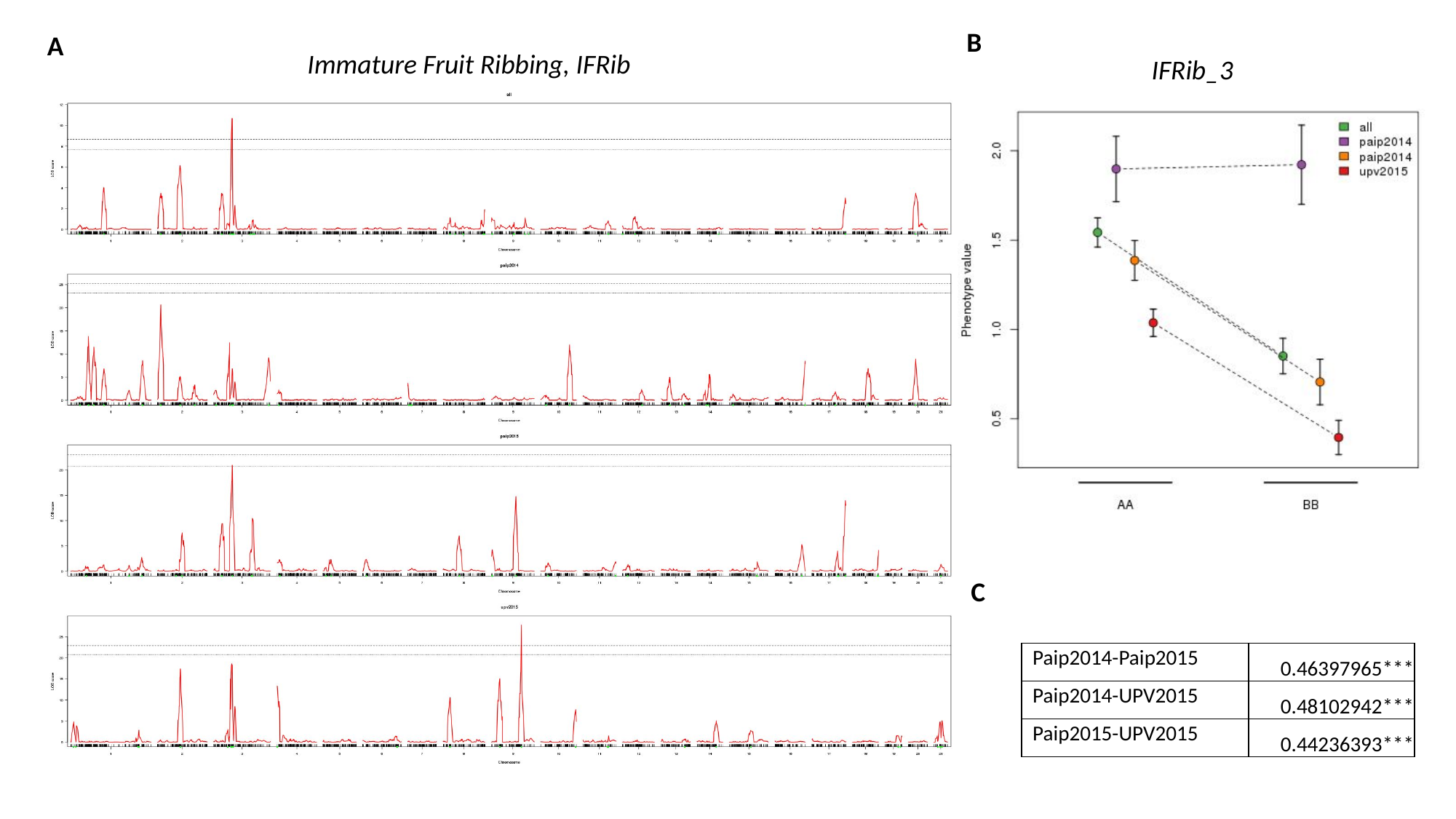

B
A
Immature Fruit Ribbing, IFRib
IFRib_3
C
| Paip2014-Paip2015 | 0.46397965\*\*\* |
| --- | --- |
| Paip2014-UPV2015 | 0.48102942\*\*\* |
| Paip2015-UPV2015 | 0.44236393\*\*\* |

## Slide 11
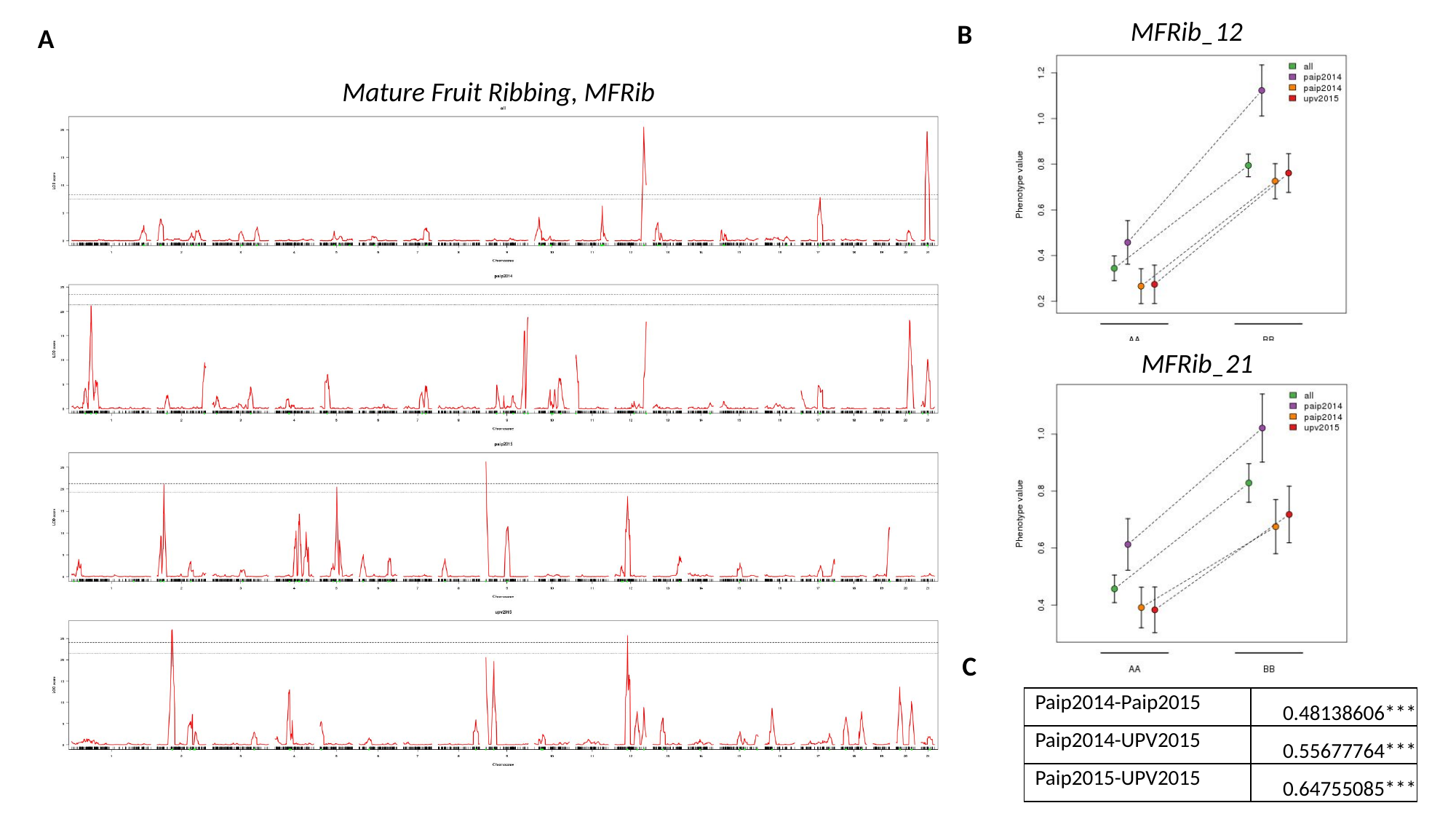

MFRib_12
B
A
Mature Fruit Ribbing, MFRib
MFRib_21
C
| Paip2014-Paip2015 | 0.48138606\*\*\* |
| --- | --- |
| Paip2014-UPV2015 | 0.55677764\*\*\* |
| Paip2015-UPV2015 | 0.64755085\*\*\* |

## Slide 12
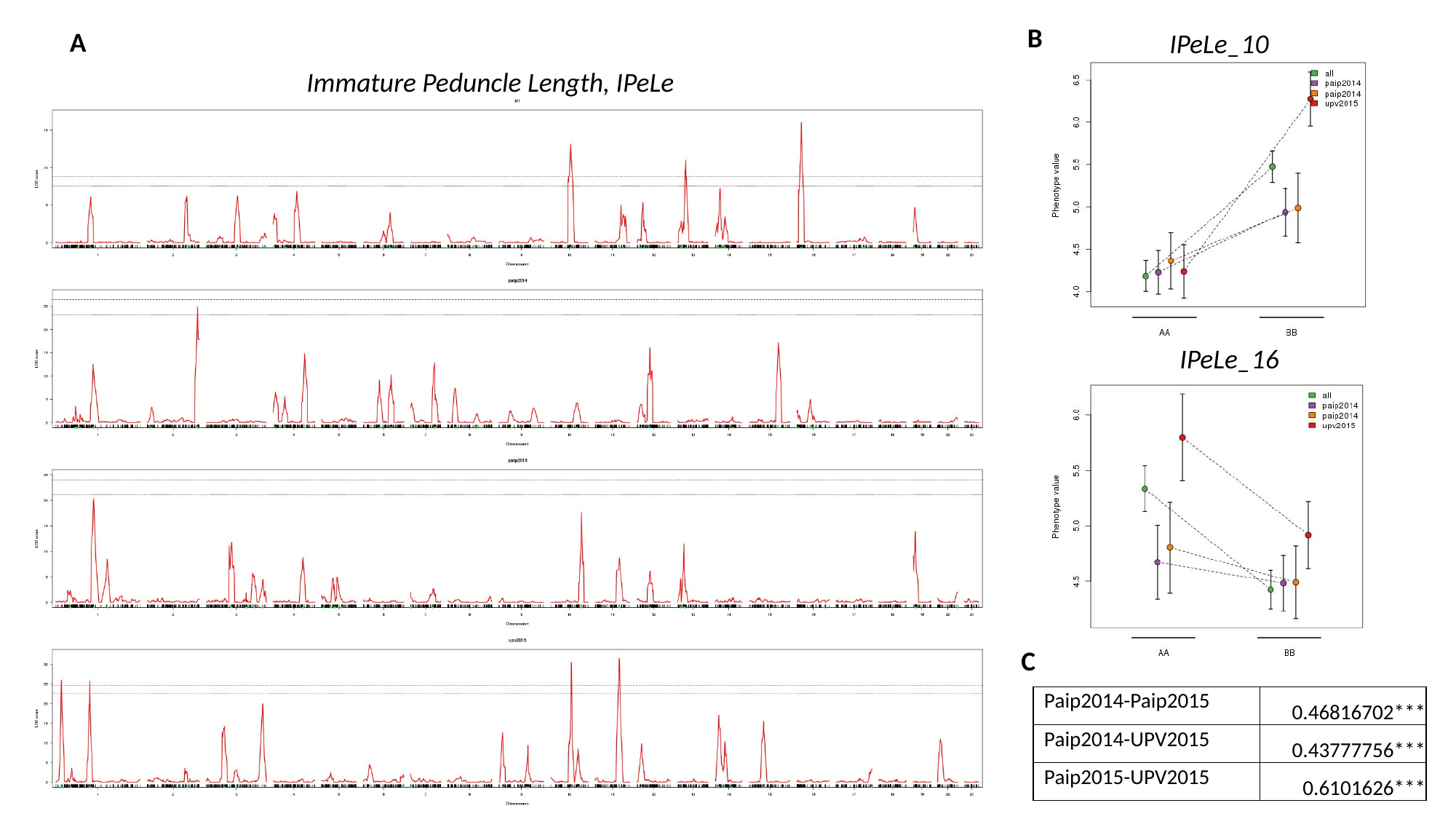

B
A
IPeLe_10
Immature Peduncle Length, IPeLe
IPeLe_16
C
| Paip2014-Paip2015 | 0.46816702\*\*\* |
| --- | --- |
| Paip2014-UPV2015 | 0.43777756\*\*\* |
| Paip2015-UPV2015 | 0.6101626\*\*\* |

## Slide 13
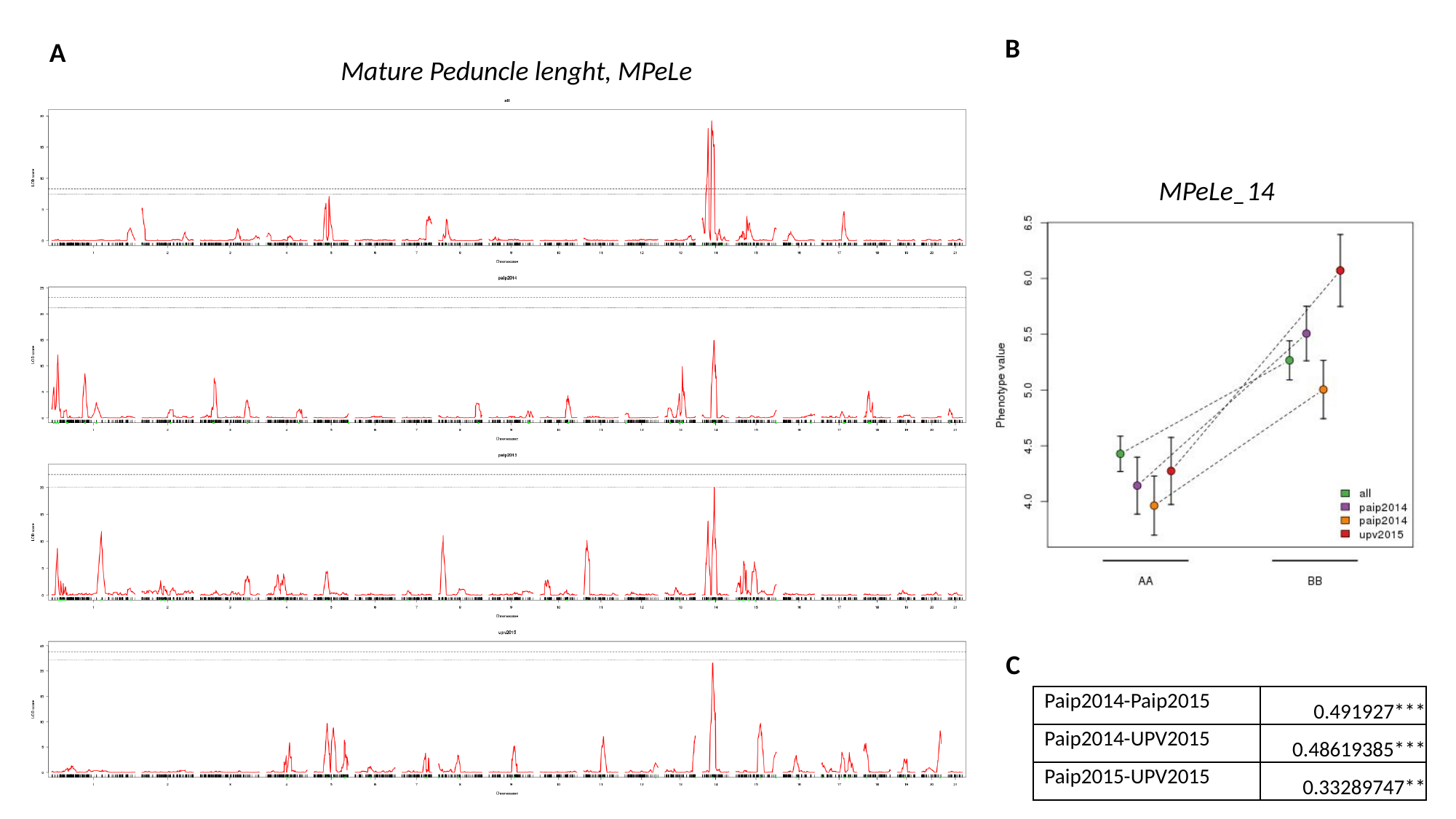

B
A
Mature Peduncle lenght, MPeLe
MPeLe_14
C
| Paip2014-Paip2015 | 0.491927\*\*\* |
| --- | --- |
| Paip2014-UPV2015 | 0.48619385\*\*\* |
| Paip2015-UPV2015 | 0.33289747\*\* |

## Slide 14
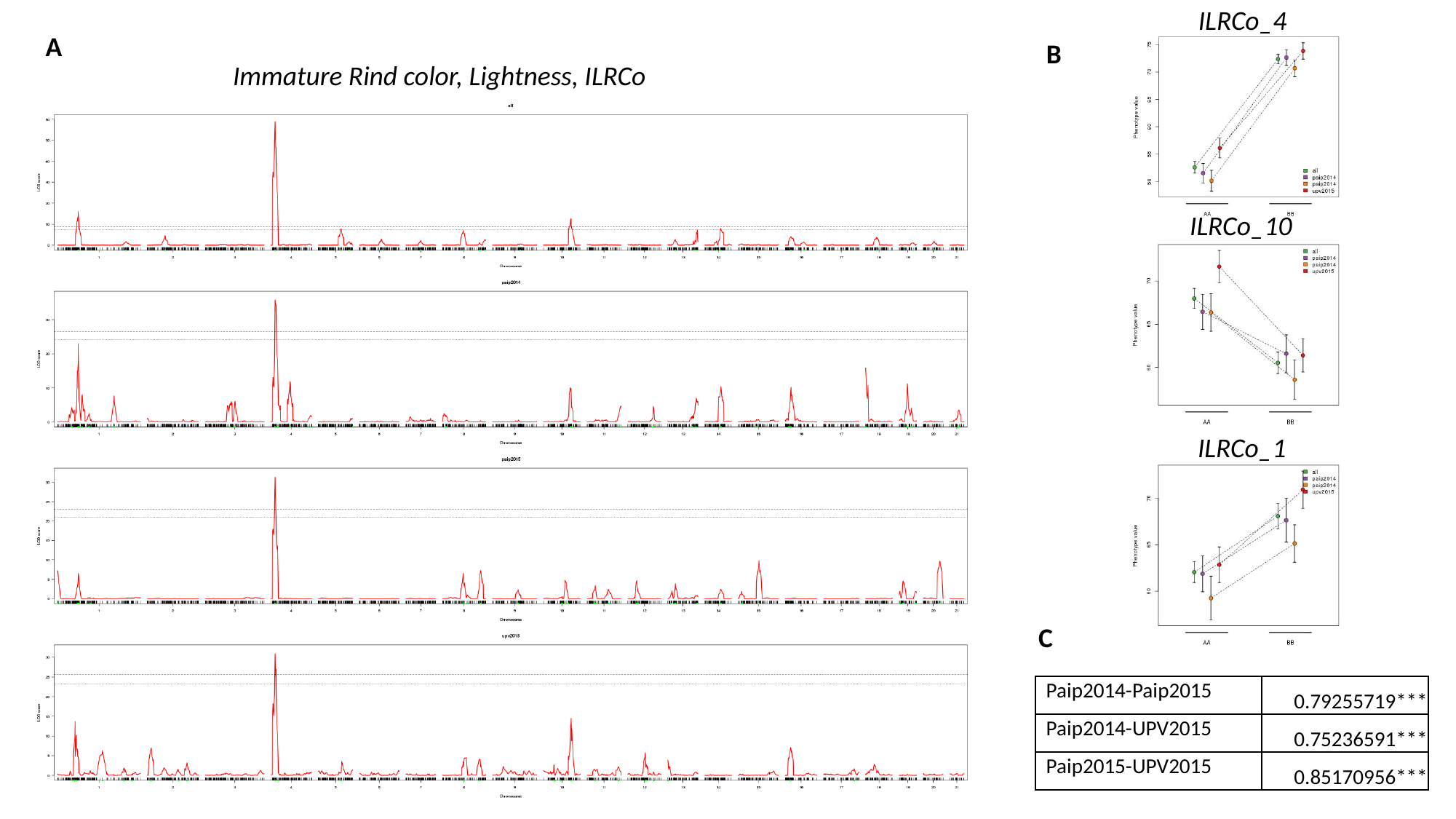

ILRCo_4
A
B
Immature Rind color, Lightness, ILRCo
ILRCo_10
ILRCo_1
C
| Paip2014-Paip2015 | 0.79255719\*\*\* |
| --- | --- |
| Paip2014-UPV2015 | 0.75236591\*\*\* |
| Paip2015-UPV2015 | 0.85170956\*\*\* |

## Slide 15
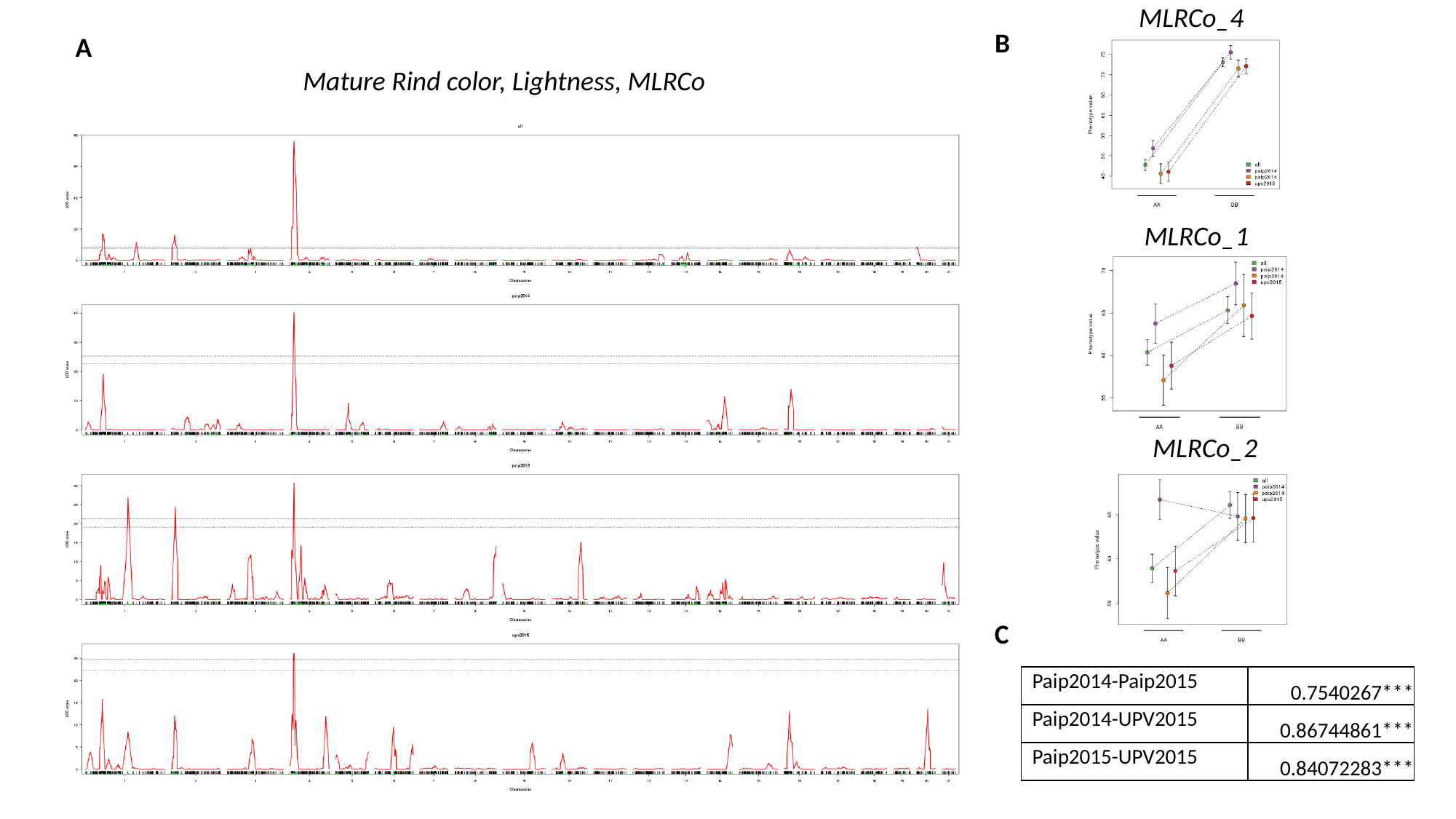

MLRCo_4
B
A
Mature Rind color, Lightness, MLRCo
MLRCo_1
MLRCo_2
C
| Paip2014-Paip2015 | 0.7540267\*\*\* |
| --- | --- |
| Paip2014-UPV2015 | 0.86744861\*\*\* |
| Paip2015-UPV2015 | 0.84072283\*\*\* |

## Slide 16
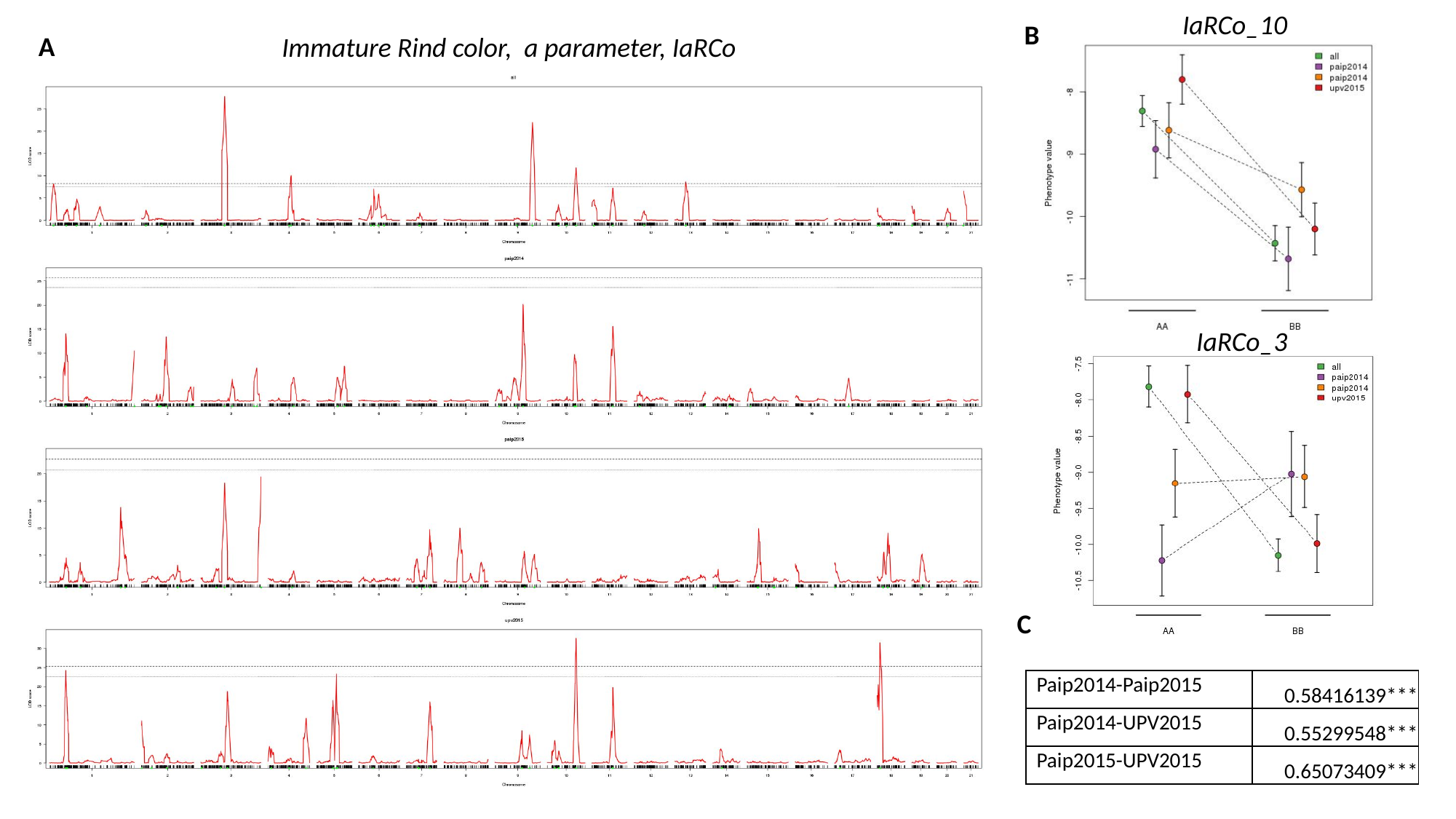

IaRCo_10
B
A
Immature Rind color, a parameter, IaRCo
IaRCo_3
C
| Paip2014-Paip2015 | 0.58416139\*\*\* |
| --- | --- |
| Paip2014-UPV2015 | 0.55299548\*\*\* |
| Paip2015-UPV2015 | 0.65073409\*\*\* |

## Slide 17
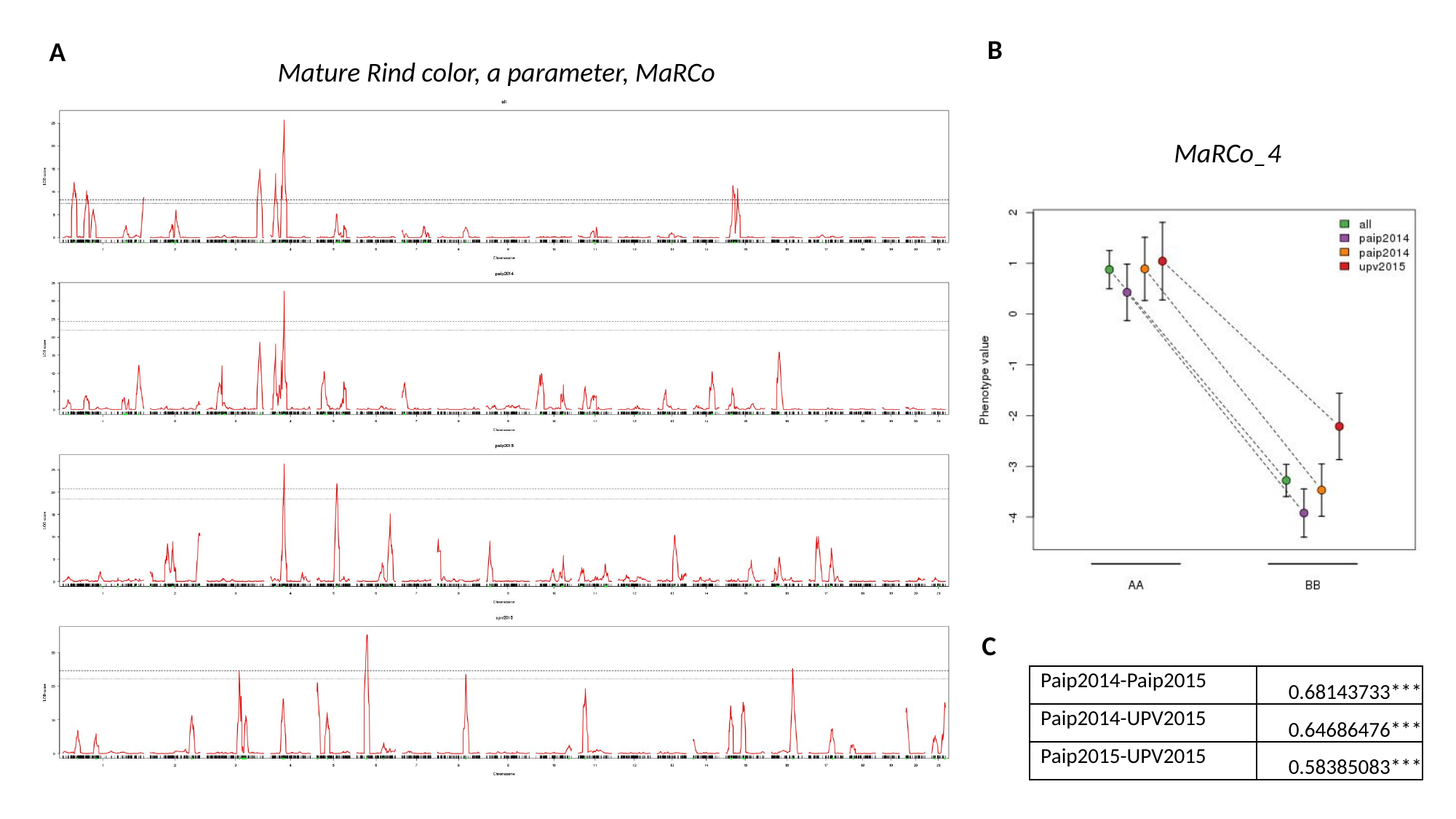

B
A
Mature Rind color, a parameter, MaRCo
MaRCo_4
C
| Paip2014-Paip2015 | 0.68143733\*\*\* |
| --- | --- |
| Paip2014-UPV2015 | 0.64686476\*\*\* |
| Paip2015-UPV2015 | 0.58385083\*\*\* |

## Slide 18
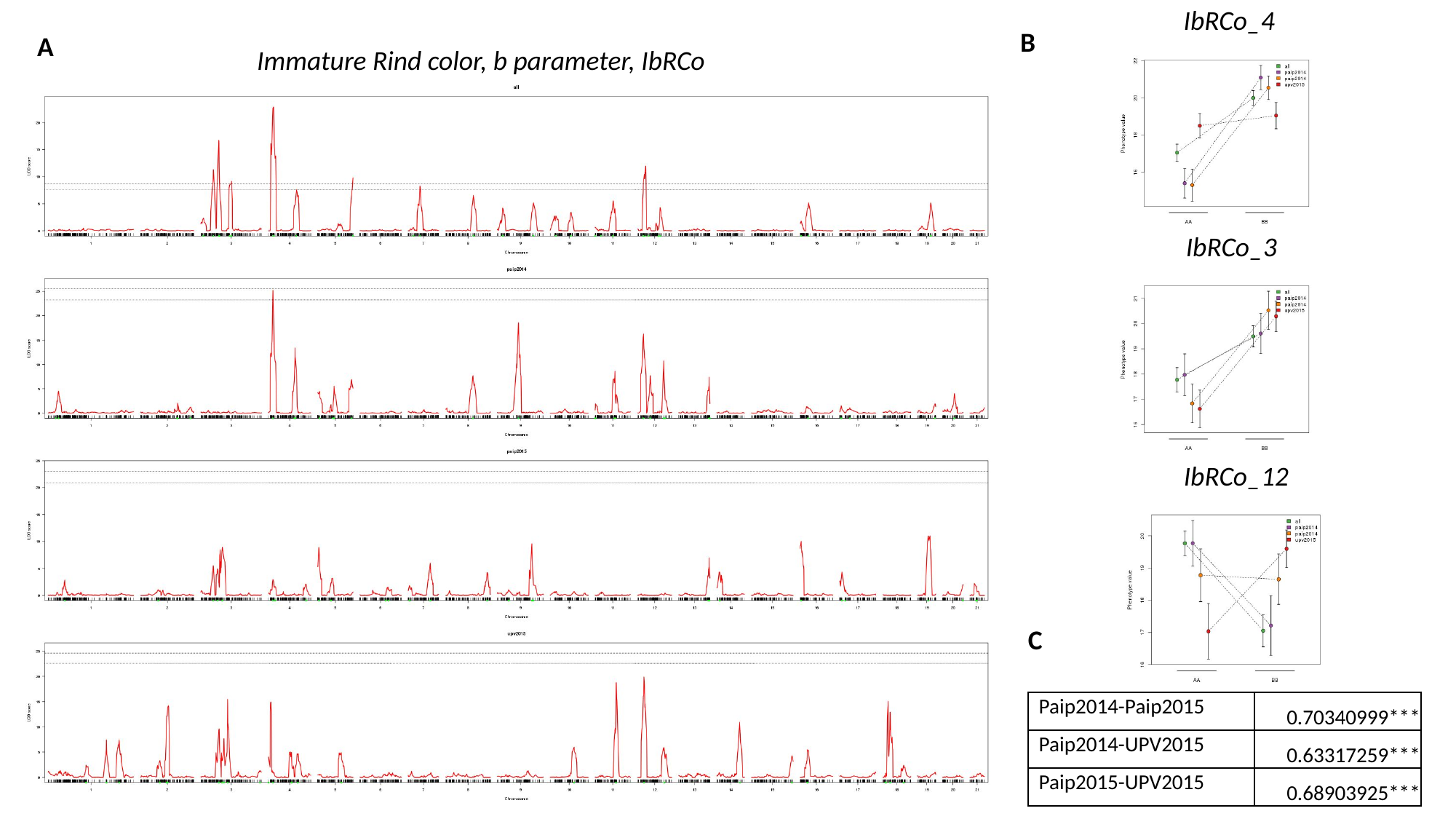

IbRCo_4
B
A
Immature Rind color, b parameter, IbRCo
IbRCo_3
IbRCo_12
C
| Paip2014-Paip2015 | 0.70340999\*\*\* |
| --- | --- |
| Paip2014-UPV2015 | 0.63317259\*\*\* |
| Paip2015-UPV2015 | 0.68903925\*\*\* |

## Slide 19
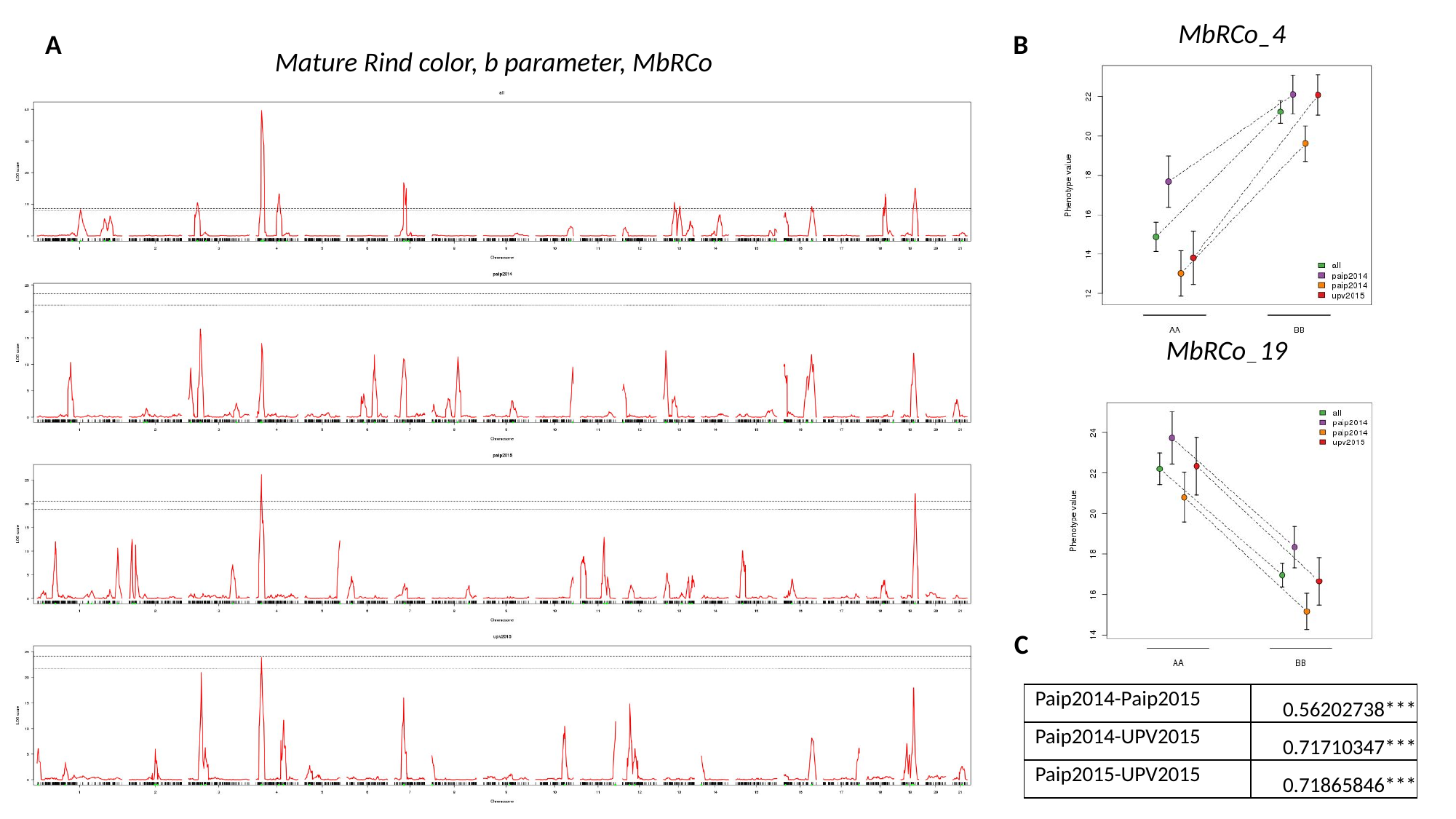

MbRCo_4
A
B
Mature Rind color, b parameter, MbRCo
MbRCo_19
C
| Paip2014-Paip2015 | 0.56202738\*\*\* |
| --- | --- |
| Paip2014-UPV2015 | 0.71710347\*\*\* |
| Paip2015-UPV2015 | 0.71865846\*\*\* |

## Slide 20
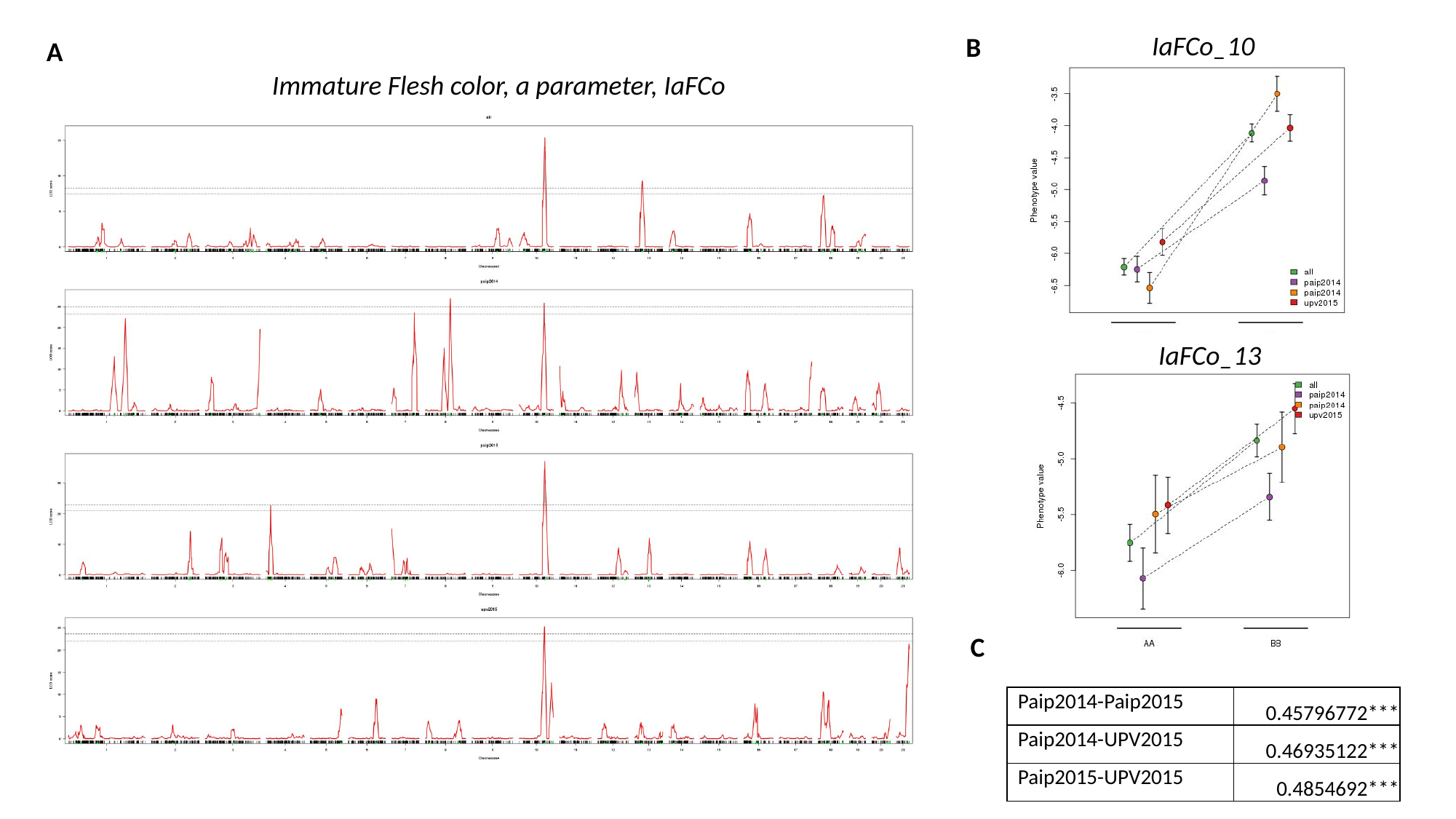

IaFCo_10
B
A
Immature Flesh color, a parameter, IaFCo
IaFCo_13
C
| Paip2014-Paip2015 | 0.45796772\*\*\* |
| --- | --- |
| Paip2014-UPV2015 | 0.46935122\*\*\* |
| Paip2015-UPV2015 | 0.4854692\*\*\* |

## Slide 21
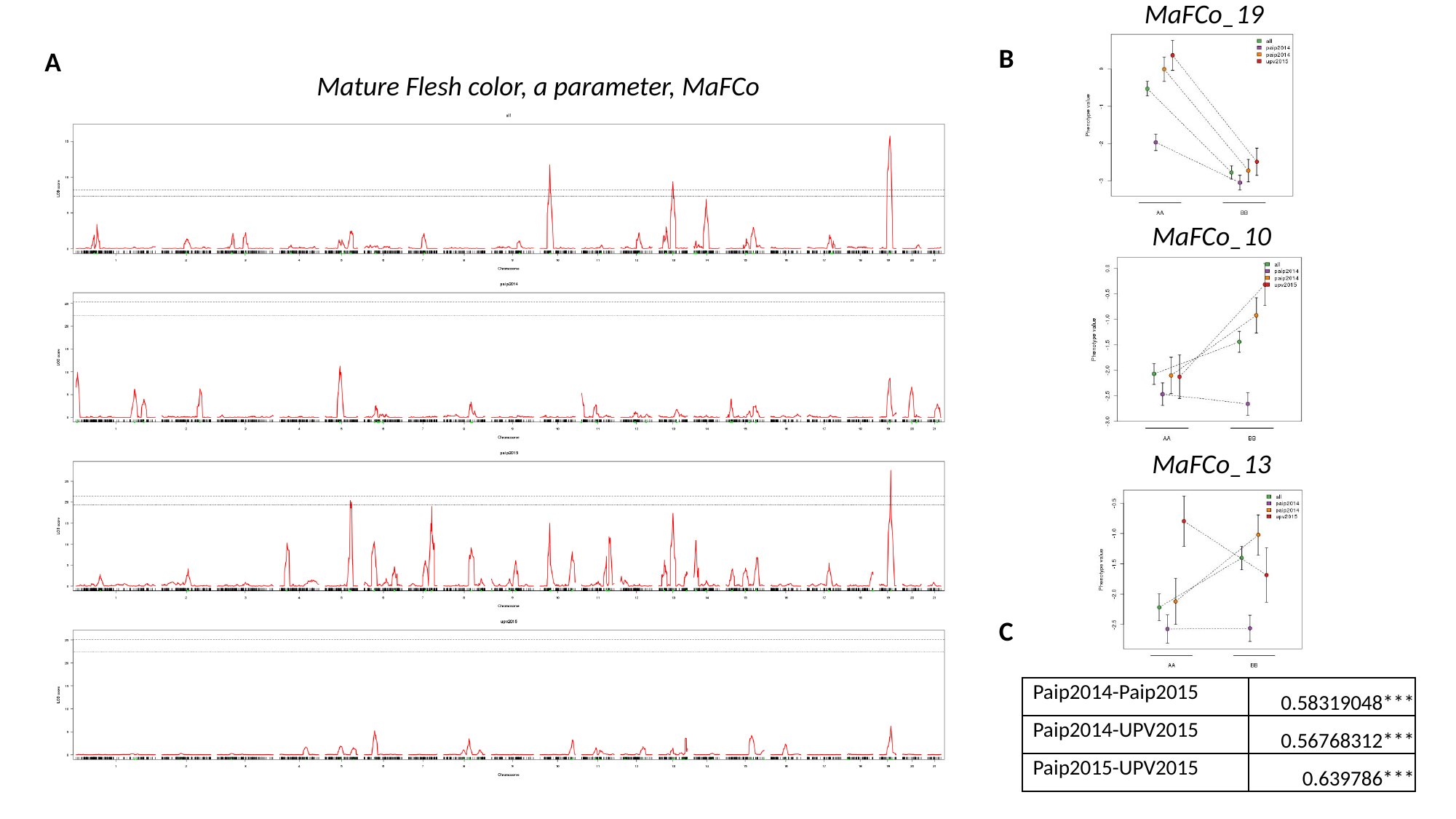

MaFCo_19
B
A
Mature Flesh color, a parameter, MaFCo
MaFCo_10
MaFCo_13
C
| Paip2014-Paip2015 | 0.58319048\*\*\* |
| --- | --- |
| Paip2014-UPV2015 | 0.56768312\*\*\* |
| Paip2015-UPV2015 | 0.639786\*\*\* |

## Slide 22
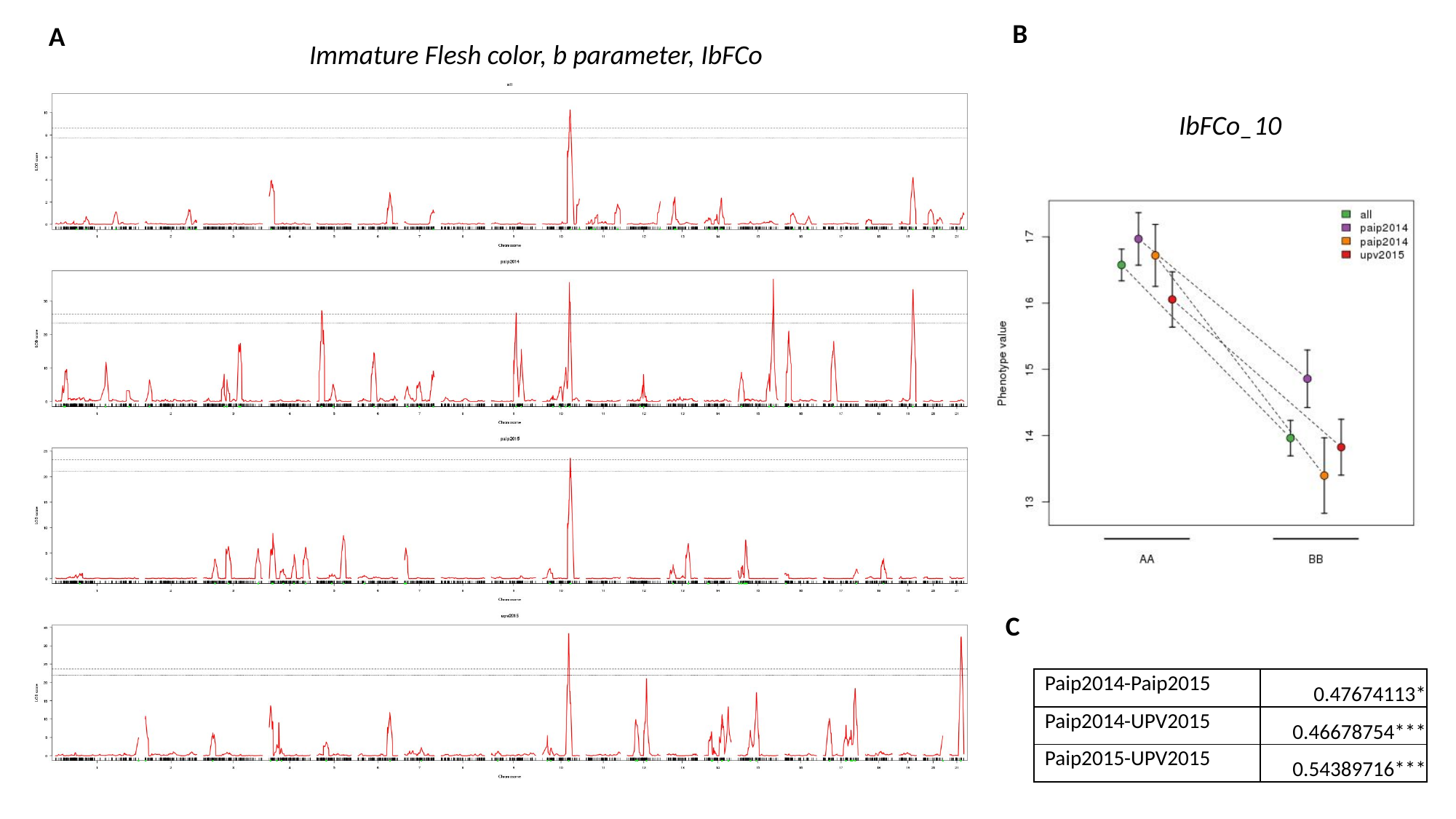

B
A
Immature Flesh color, b parameter, IbFCo
IbFCo_10
C
| Paip2014-Paip2015 | 0.47674113\* |
| --- | --- |
| Paip2014-UPV2015 | 0.46678754\*\*\* |
| Paip2015-UPV2015 | 0.54389716\*\*\* |

## Slide 23
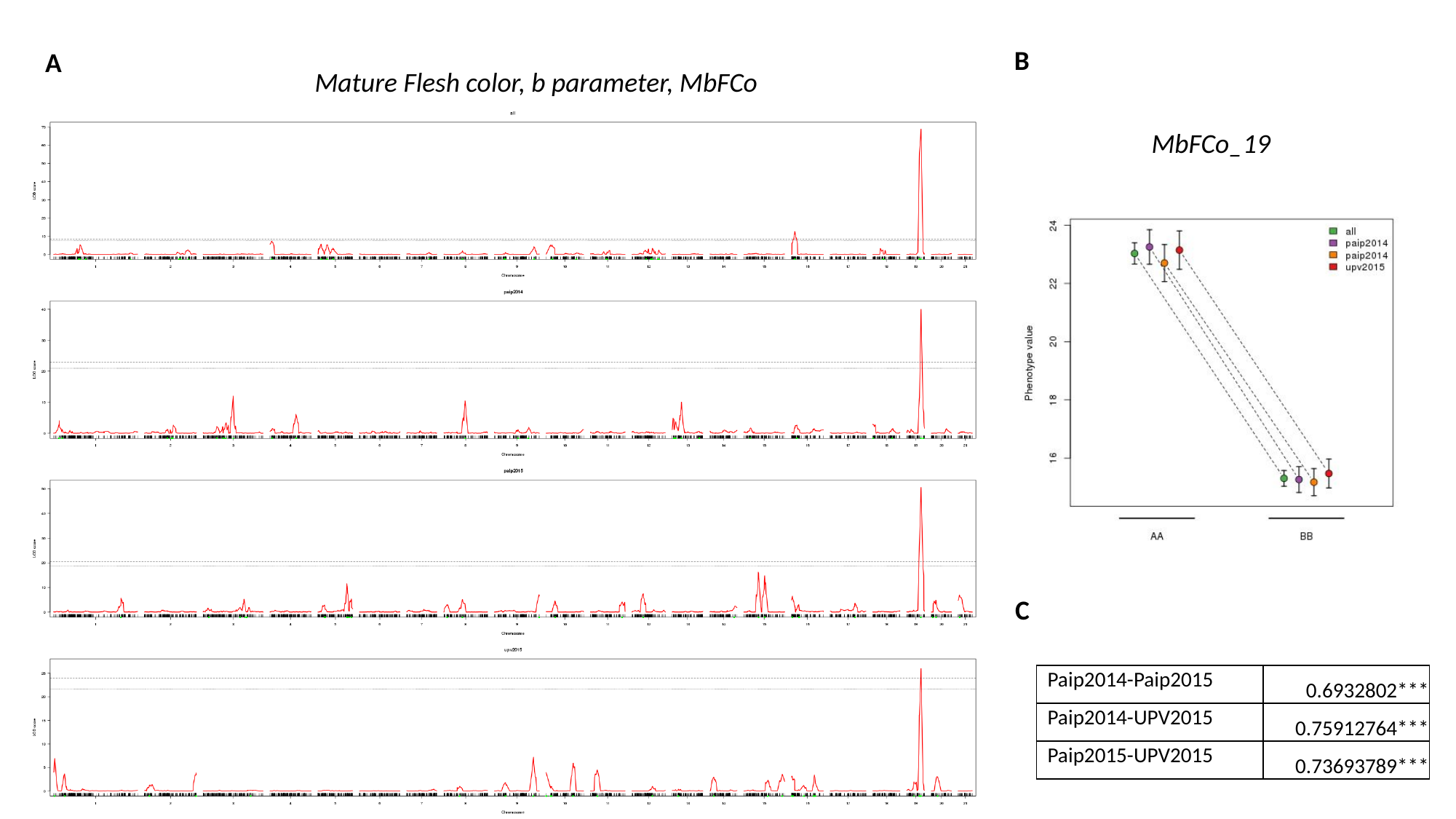

B
A
Mature Flesh color, b parameter, MbFCo
MbFCo_19
C
| Paip2014-Paip2015 | 0.6932802\*\*\* |
| --- | --- |
| Paip2014-UPV2015 | 0.75912764\*\*\* |
| Paip2015-UPV2015 | 0.73693789\*\*\* |
