# Supplementary material for: An SNP-based saturated genetic map and QTL analysis of fruit-related traits in Zucchini using Genotyping-by-sequencing
Source: BMC Genomics. 2017 Jan 18;18:94. doi: 10.1186/s12864-016-3439-y (PMC5241963; doi:10.1186/s12864-016-3439-y)
Supplement: Additional file 5: — Phenotypic values of RILs carrying Zucchini and Scallop alleles in the detected QTL regions. Means and standard errors of the phenotypic value of RILs belonging to the alternative allelic classes (Zuchini versus Scallop) for the markers in the LOD peak regions of each significant QTL are shown. Data of the three environments, combined and separate, are shown to validate the effects of the different QTLs (Paip2014, Paip2015, and UPV2015). (DOCX 39 kb) [file 12864_2016_3439_MOESM5_ESM.docx]

**Additional file 5**. **Phenotypic values of RILs carrying Zucchini and Scallop alleles in the detected QTL regions.**

| **QTL** | **assay** | **AA_means** | **BB_means** | **AA_SE** | **BB_SE** |  |  |  |  |  |  |
| --- | --- | --- | --- | --- | --- | --- | --- | --- | --- | --- | --- |
| *Li_10* | all | 2.97803449 | 1.82833341 | 0.04380094 | 0.04294079 |  |  |  |  |  |  |
| *Li_10* | Paip2014 | 3.17794986 | 2.10975481 | 0.08090885 | 0.07958582 |  |  |  |  |  |  |
| *Li_10* | Paip2015 | 2.9122807 | 1.76271186 | 0.07300417 | 0.07175614 |  |  |  |  |  |  |
| *Li_10* | UPV2015 | 2.80408613 | 1.61637644 | 0.06337091 | 0.06246136 |  |  |  |  |  |  |
| *Sl_12* | all | 0.48503447 | 0.11085275 | 0.02880332 | 0.02792192 |  |  |  |  |  |  |
| *Sl_12* | Paip2014 | 0.53813559 | 0.14836066 | 0.05216571 | 0.05130341 |  |  |  |  |  |  |
| *Sl_12* | Paip2015 | 0.46918663 | 0.08914377 | 0.0465262 | 0.04586445 |  |  |  |  |  |  |
| *Sl_12* | UPV2015 | 0.45011821 | 0.07679354 | 0.0484587 | 0.04725518 |  |  |  |  |  |  |
| *Sl_1* | all | 0.37361442 | 0.24664298 | 0.03707082 | 0.02770733 |  |  |  |  |  |  |
| *Sl_1* | Paip2014 | 0.24058798 | 0.42217687 | 0.05931691 | 0.05400846 |  |  |  |  |  |  |
| *Sl_1* | Paip2015 | 0.4793277 | 0.07988876 | 0.04568064 | 0.04490881 |  |  |  |  |  |  |
| *Sl_1* | UPV2015 | 0.41510483 | 0.0439447 | 0.04942536 | 0.0575959 |  |  |  |  |  |  |
| *Sl_16* | all | 0.1593973 | 0.37332681 | 0.03663981 | 0.02809586 |  |  |  |  |  |  |
| *Sl_16* | Paip2014 | 0.30768382 | 0.35924269 | 0.06667043 | 0.05140453 |  |  |  |  |  |  |
| *Sl_16* | Paip2015 | 0.26808511 | 0.2826087 | 0.05806437 | 0.04792191 |  |  |  |  |  |  |
| *Sl_16* | UPV2015 | 0.12922646 | 0.34756676 | 0.06437188 | 0.05058423 |  |  |  |  |  |  |
| *DFeF_12* | all | 34.2450664 | 39.6541687 | 0.62051289 | 0.76587071 |  |  |  |  |  |  |
| *DFeF_12* | Paip2014 | 37.0981522 | 44.7798654 | 1.23376341 | 1.5253576 |  |  |  |  |  |  |
| *DFeF_12* | Paip2015 | 34.7192982 | 37.3728814 | 0.71172292 | 0.69955582 |  |  |  |  |  |  |
| *DFeF_12* | UPV2015 | 30.9218753 | 36.1185528 | 1.02213306 | 1.31523612 |  |  |  |  |  |  |
| *DFeF_9* | all | 34.9765362 | 37.4005274 | 0.77208691 | 0.64982236 |  |  |  |  |  |  |
| *DFeF_9* | Paip2014 | 36.6676727 | 42.685974 | 1.50905152 | 1.29345246 |  |  |  |  |  |  |
| *DFeF_9* | Paip2015 | 34.782694 | 36.8018626 | 0.84691244 | 0.63648597 |  |  |  |  |  |  |
| *DFeF_9* | UPV2015 | 32.8484716 | 32.8965486 | 1.40347577 | 1.05101305 |  |  |  |  |  |  |
| *IFSh_3* | all | 5.33088192 | 4.53354695 | 0.07196476 | 0.08198492 |  |  |  |  |  |  |
| *IFSh_3* | Paip2014 | 5.33076664 | 4.48366007 | 0.11495206 | 0.13046198 |  |  |  |  |  |  |
| *IFSh_3* | Paip2015 | 5.4201625 | 4.49453256 | 0.11823974 | 0.13092663 |  |  |  |  |  |  |
| *IFSh_3* | UPV2015 | 5.25880528 | 4.63905166 | 0.10336478 | 0.10762089 |  |  |  |  |  |  |
| *IFLe_3* | all | 14.499829 | 10.2563179 | 0.26456005 | 0.2952427 |  |  |  |  |  |  |
| *IFLe_3* | Paip2014 | 13.5667066 | 9.7794472 | 0.3658198 | 0.40379213 |  |  |  |  |  |  |
| *IFLe_3* | Paip2015 | 15.118195 | 10.5718462 | 0.45941766 | 0.46445578 |  |  |  |  |  |  |
| *IFLe_3* | UPV2015 | 14.8071206 | 10.4397543 | 0.42263154 | 0.486846 |  |  |  |  |  |  |
| *IFWi_3* | all | 5.75679278 | 7.11221955 | 0.1079962 | 0.12150555 |  |  |  |  |  |  |
| *IFWi_3* | Paip2014 | 5.52820018 | 6.76343292 | 0.19413611 | 0.22194622 |  |  |  |  |  |  |
| *IFWi_3* | Paip2015 | 5.89167244 | 7.48658872 | 0.1846108 | 0.1885332 |  |  |  |  |  |  |
| *IFWi_3* | UPV2015 | 5.839003 | 7.00842505 | 0.19630603 | 0.22191682 |  |  |  |  |  |  |
| *MFSh_3* | all | 5.16240679 | 4.63708961 | 0.05582398 | 0.06306141 |  |  |  |  |  |  |
| *MFSh_3* | Paip2014 | 5.2298147 | 4.58101647 | 0.12312995 | 0.13799608 |  |  |  |  |  |  |
| *MFSh_3* | Paip2015 | 5.17665577 | 4.66906451 | 0.09175196 | 0.09570219 |  |  |  |  |  |  |
| *MFSh_3* | UPV2015 | 5.11433805 | 4.66298891 | 0.08778381 | 0.10270411 |  |  |  |  |  |  |
| *MFLe_3* | all | 24.9840264 | 16.005231 | 0.47485478 | 0.50108429 |  |  |  |  |  |  |
| *MFLe_3* | Paip2014 | 24.5222907 | 15.7502056 | 0.8056164 | 0.8746846 |  |  |  |  |  |  |
| *MFLe_3* | Paip2015 | 26.5816575 | 16.593584 | 0.85299715 | 0.76287263 |  |  |  |  |  |  |
| *MFLe_3* | UPV2015 | 23.9326891 | 15.470343 | 0.71275386 | 0.86020373 |  |  |  |  |  |  |
| *MFWi_3* | all | 10.0956076 | 11.6328209 | 0.13499249 | 0.15511682 |  |  |  |  |  |  |
| *MFWi_3* | Paip2014 | 10.2990088 | 10.6351693 | 0.28945522 | 0.28081685 |  |  |  |  |  |  |
| *MFWi_3* | Paip2015 | 10.6433994 | 11.9924918 | 0.22804359 | 0.22380509 |  |  |  |  |  |  |
| *MFWi_3* | UPV2015 | 10.0088694 | 11.0491211 | 0.24152398 | 0.28080973 |  |  |  |  |  |  |
| *IFSh_12* | all | 5.15230769 | 4.65185185 | 0.06391584 | 0.08588433 |  |  |  |  |  |  |
| *IFSh_12* | Paip2014 | 5.22220533 | 4.52633443 | 0.11364978 | 0.14633566 |  |  |  |  |  |  |
| *IFSh_12* | Paip2015 | 5.12341202 | 4.88731754 | 0.14093593 | 0.13466868 |  |  |  |  |  |  |
| *IFSh_12* | UPV2015 | 5.13181818 | 4.62941176 | 0.09483141 | 0.13212491 |  |  |  |  |  |  |
| *IFLe_12* | all | 13.3590625 | 11.2738739 | 0.25258006 | 0.33219125 |  |  |  |  |  |  |
| *IFLe_12* | Paip2014 | 12.709375 | 10.4972973 | 0.38225735 | 0.50274177 |  |  |  |  |  |  |
| *IFLe_12* | Paip2015 | 13.1261011 | 12.8075638 | 0.53098095 | 0.50752044 |  |  |  |  |  |  |
| *IFLe_12* | UPV2015 | 13.123335 | 12.75771 | 0.56785315 | 0.52510519 |  |  |  |  |  |  |
| *MFLe_12* | all | 22.2853232 | 18.5342463 | 0.48737576 | 0.62642743 |  |  |  |  |  |  |
| *MFLe_12* | Paip2014 | 22.1458972 | 19.6091784 | 1.15865367 | 0.83807819 |  |  |  |  |  |  |
| *MFLe_12* | Paip2015 | 24.4964448 | 20.226885 | 1.13975475 | 0.8330763 |  |  |  |  |  |  |
| *MFLe_12* | UPV2015 | 21.4454434 | 18.9299337 | 0.89005387 | 0.99845608 |  |  |  |  |  |  |
| *MFWi_12* | all | 10.3741294 | 11.4734513 | 0.13145678 | 0.17532418 |  |  |  |  |  |  |
| *MFWi_12* | Paip2014 | 9.94562211 | 11.4666578 | 0.23149602 | 0.31816758 |  |  |  |  |  |  |
| *MFWi_12* | Paip2015 | 11.1311692 | 11.6171764 | 0.21890139 | 0.25740012 |  |  |  |  |  |  |
| *MFWi_12* | UPV2015 | 10.0727213 | 10.8387843 | 0.27409787 | 0.27940728 |  |  |  |  |  |  |
| *IFLe_15* | all | 13.7142857 | 11.7985311 | 0.31328711 | 0.26432678 |  |  |  |  |  |  |
| *IFLe_15* | Paip2014 | 12.1265411 | 11.6483569 | 0.44557692 | 0.4682747 |  |  |  |  |  |  |
| *IFLe_15* | Paip2015 | 14.25 | 11.9 | 0.51833703 | 0.46978283 |  |  |  |  |  |  |
| *IFLe_15* | UPV2015 | 12.8976349 | 12.9565075 | 0.54007236 | 0.55050127 |  |  |  |  |  |  |
| *MFSh_4* | all | 5.05482167 | 4.85726429 | 0.07294939 | 0.05602369 |  |  |  |  |  |  |
| *MFSh_4* | Paip2014 | 5.26747407 | 4.73697288 | 0.14073793 | 0.10856147 |  |  |  |  |  |  |
| *MFSh_4* | Paip2015 | 5.10262748 | 4.84242315 | 0.11376487 | 0.08464613 |  |  |  |  |  |  |
| *MFSh_4* | UPV2015 | 4.93305965 | 4.91596054 | 0.11827674 | 0.08789561 |  |  |  |  |  |  |
| *MFSh_5* | all | 5.07793103 | 4.80473373 | 0.06405055 | 0.05932852 |  |  |  |  |  |  |
| *MFSh_5* | Paip2014 | 5.11702128 | 4.78421053 | 0.13045164 | 0.11845708 |  |  |  |  |  |  |
| *MFSh_5* | Paip2015 | 5.08867925 | 4.79482759 | 0.09756656 | 0.09326634 |  |  |  |  |  |  |
| *MFSh_5* | UPV2015 | 4.75859696 | 5.04199055 | 0.10566914 | 0.09039032 |  |  |  |  |  |  |
| *MFLe_6* | all | 22.0390842 | 19.3529425 | 0.52193886 | 0.59338127 |  |  |  |  |  |  |
| *MFLe_6* | Paip2014 | 21.7407721 | 19.0595885 | 0.92732235 | 0.9853085 |  |  |  |  |  |  |
| *MFLe_6* | Paip2015 | 22.4570437 | 20.7478501 | 0.92873092 | 1.0570533 |  |  |  |  |  |  |
| *MFLe_6* | UPV2015 | 22.0696316 | 18.8907904 | 0.97031358 | 0.87947392 |  |  |  |  |  |  |
| *MFLe_9* | all | 19.0487498 | 22.7192891 | 0.57672257 | 0.57431251 |  |  |  |  |  |  |
| *MFLe_9* | Paip2014 | 20.5212138 | 20.4554758 | 1.07832802 | 0.89483058 |  |  |  |  |  |  |
| *MFLe_9* | Paip2015 | 20.4373126 | 22.5476981 | 1.09894966 | 0.89005569 |  |  |  |  |  |  |
| *MFLe_9* | UPV2015 | 16.6735916 | 23.0311719 | 0.91038462 | 0.7752891 |  |  |  |  |  |  |
| *IFRib_3* | all | 1.54292148 | 0.8514198 | 0.08186495 | 0.09938293 |  |  |  |  |  |  |
| *IFRib_3* | Paip2014 | 1.89831963 | 1.92196933 | 0.18301992 | 0.22139324 |  |  |  |  |  |  |
| *IFRib_3* | Paip2015 | 1.38625659 | 0.70627091 | 0.11157577 | 0.12742194 |  |  |  |  |  |  |
| *IFRib_3* | UPV2015 | 1.03741638 | 0.39576746 | 0.07637768 | 0.09569642 |  |  |  |  |  |  |
| *MFRib_12* | all | 0.34426052 | 0.79466634 | 0.05424994 | 0.04964468 |  |  |  |  |  |  |
| *MFRib_12* | Paip2014 | 0.45768256 | 1.12276227 | 0.09544112 | 0.11164789 |  |  |  |  |  |  |
| *MFRib_12* | Paip2015 | 0.26607143 | 0.72545455 | 0.07636562 | 0.07705673 |  |  |  |  |  |  |
| *MFRib_12* | UPV2015 | 0.274 | 0.76122449 | 0.08398235 | 0.08483499 |  |  |  |  |  |  |
| *MFRib_21* | all | 0.45701572 | 0.82847826 | 0.04830566 | 0.06772574 |  |  |  |  |  |  |
| *MFRib_21* | Paip2014 | 0.61278578 | 1.02126174 | 0.09040895 | 0.11943922 |  |  |  |  |  |  |
| *MFRib_21* | Paip2015 | 0.39148205 | 0.675214 | 0.07127996 | 0.09500461 |  |  |  |  |  |  |
| *MFRib_21* | UPV2015 | 0.38332109 | 0.71794769 | 0.08013288 | 0.09938736 |  |  |  |  |  |  |
| *IPeLe_10* | all | 4.18451613 | 5.47342657 | 0.18018518 | 0.18759312 |  |  |  |  |  |  |
| *IPeLe_10* | Paip2014 | 4.22921586 | 4.93724008 | 0.25700191 | 0.27996851 |  |  |  |  |  |  |
| *IPeLe_10* | Paip2015 | 4.36443818 | 4.98654197 | 0.33290586 | 0.41290478 |  |  |  |  |  |  |
| *IPeLe_10* | UPV2015 | 4.23843027 | 6.27373057 | 0.31305073 | 0.32006737 |  |  |  |  |  |  |
| *IPeLe_16* | all | 5.33452125 | 4.42516038 | 0.2066743 | 0.17407908 |  |  |  |  |  |  |
| *IPeLe_16* | Paip2014 | 4.67177536 | 4.48175253 | 0.33531417 | 0.2516984 |  |  |  |  |  |  |
| *IPeLe_16* | Paip2015 | 4.80384615 | 4.48934426 | 0.40877788 | 0.32685473 |  |  |  |  |  |  |
| *IPeLe_16* | UPV2015 | 5.7972973 | 4.91612903 | 0.39171519 | 0.30260453 |  |  |  |  |  |  |
| *MPeLe_14* | all | 4.42820684 | 5.26621901 | 0.15852626 | 0.17490494 |  |  |  |  |  |  |
| *MPeLe_14* | Paip2014 | 4.14305353 | 5.50653813 | 0.25528716 | 0.24469744 |  |  |  |  |  |  |
| *MPeLe_14* | Paip2015 | 3.9642491 | 5.00474821 | 0.26490027 | 0.2615102 |  |  |  |  |  |  |
| *MPeLe_14* | UPV2015 | 4.27452662 | 6.07171043 | 0.30071744 | 0.32256513 |  |  |  |  |  |  |
| *ILRCo_4* | all | 52.6111264 | 72.3503944 | 1.07462287 | 0.86769132 |  |  |  |  |  |  |
| *ILRCo_4* | Paip2014 | 51.5354144 | 72.6281492 | 1.7642994 | 1.43009118 |  |  |  |  |  |  |
| *ILRCo_4* | Paip2015 | 50.1624923 | 70.6681479 | 1.91614928 | 1.52958541 |  |  |  |  |  |  |
| *ILRCo_4* | UPV2015 | 56.1220869 | 73.8362397 | 1.81255752 | 1.48218151 |  |  |  |  |  |  |
| *IbRCo_4* | all | 16.133178 | 20.5143872 | 0.45803343 | 0.37346308 |  |  |  |  |  |  |
| *IbRCo_4* | Paip2014 | 15.4077347 | 21.1058526 | 0.79582168 | 0.65019373 |  |  |  |  |  |  |
| *IbRCo_4* | Paip2015 | 18.8102679 | 18.588587 | 0.76440136 | 0.84340574 |  |  |  |  |  |  |
| *IbRCo_4* | UPV2015 | 17.6517244 | 19.7087709 | 0.69713542 | 0.64561439 |  |  |  |  |  |  |
| *MLRCo_4* | all | 47.7604482 | 73.0588887 | 1.3520517 | 1.10881628 |  |  |  |  |  |  |
| *MLRCo_4* | Paip2014 | 51.8924233 | 75.5399175 | 2.02605837 | 1.66148647 |  |  |  |  |  |  |
| *MLRCo_4* | Paip2015 | 45.5864261 | 71.5450805 | 2.46615069 | 2.07656858 |  |  |  |  |  |  |
| *MLRCo_4* | UPV2015 | 46.0786993 | 72.1292077 | 2.34564132 | 1.90540423 |  |  |  |  |  |  |
| *MbRCo_4* | all | 14.8850067 | 21.2198789 | 0.73570996 | 0.57453842 |  |  |  |  |  |  |
| *MbRCo_4* | Paip2014 | 17.6721788 | 22.0975737 | 1.30177027 | 0.98739112 |  |  |  |  |  |  |
| *MbRCo_4* | Paip2015 | 13.0271116 | 19.6084764 | 1.15692435 | 0.89970775 |  |  |  |  |  |  |
| *MbRCo_4* | UPV2015 | 13.8109784 | 22.0715994 | 1.34785998 | 1.03383566 |  |  |  |  |  |  |
| *ILRCo_10* | all | 67.984125 | 60.5287943 | 1.16126067 | 1.23702977 |  |  |  |  |  |  |
| *ILRCo_10* | Paip2014 | 66.4459078 | 61.5767544 | 2.0358158 | 2.20500941 |  |  |  |  |  |  |
| *ILRCo_10* | Paip2015 | 66.3529706 | 58.5703313 | 2.17979655 | 2.27317246 |  |  |  |  |  |  |
| *ILRCo_10* | UPV2015 | 71.6805882 | 61.3852083 | 1.87185095 | 1.92945979 |  |  |  |  |  |  |
| *IaRCo_10* | all | -8.30871721 | -10.428782 | 0.24780319 | 0.28325555 |  |  |  |  |  |  |
| *IaRCo_10* | Paip2014 | -8.92254545 | -10.6795556 | 0.45987608 | 0.50841213 |  |  |  |  |  |  |
| *IaRCo_10* | Paip2015 | -8.6178 | -9.57057692 | 0.44238058 | 0.43378985 |  |  |  |  |  |  |
| *IaRCo_10* | UPV2015 | -7.80354856 | -10.200166 | 0.39515017 | 0.41556208 |  |  |  |  |  |  |
| *IaFCo_10* | all | -6.21024691 | -4.115 | 0.1306019 | 0.14099367 |  |  |  |  |  |  |
| *IaFCo_10* | Paip2014 | -6.24527273 | -4.86177778 | 0.19942318 | 0.22047062 |  |  |  |  |  |  |
| *IaFCo_10* | Paip2015 | -6.53660714 | -3.50054348 | 0.24456099 | 0.26983749 |  |  |  |  |  |  |
| *IaFCo_10* | UPV2015 | -5.82001314 | -4.03476724 | 0.20910911 | 0.21122678 |  |  |  |  |  |  |
| *IbFCo_10* | all | 16.5768862 | 13.9640226 | 0.23892461 | 0.2677277 |  |  |  |  |  |  |
| *IbFCo_10* | Paip2014 | 16.97 | 14.8593333 | 0.39596711 | 0.43376024 |  |  |  |  |  |  |
| *IbFCo_10* | Paip2015 | 16.9021053 | 13.2721111 | 0.42645497 | 0.47995923 |  |  |  |  |  |  |
| *IbFCo_10* | UPV2015 | 16.2605294 | 13.6137322 | 0.40187515 | 0.4052741 |  |  |  |  |  |  |
| *IaRCo_3* | all | -7.81672008 | -10.1509453 | 0.28437915 | 0.22498504 |  |  |  |  |  |  |
| *IaRCo_3* | Paip2014 | -10.2196165 | -9.02352857 | 0.49282975 | 0.58908981 |  |  |  |  |  |  |
| *IaRCo_3* | Paip2015 | -9.15324682 | -9.06075928 | 0.46521618 | 0.42758153 |  |  |  |  |  |  |
| *IaRCo_3* | UPV2015 | -7.92083525 | -9.98890299 | 0.39565589 | 0.40414484 |  |  |  |  |  |  |
| *ILRCo_1* | all | 62.0591267 | 68.0768644 | 1.11835164 | 1.36059594 |  |  |  |  |  |  |
| *ILRCo_1* | Paip2014 | 61.8811393 | 67.6395388 | 1.93361837 | 2.35517726 |  |  |  |  |  |  |
| *ILRCo_1* | Paip2015 | 59.2749506 | 65.1372831 | 2.34299608 | 2.01714016 |  |  |  |  |  |  |
| *ILRCo_1* | UPV2015 | 62.8517004 | 70.9283365 | 1.90704291 | 2.00414429 |  |  |  |  |  |  |
| *IbRCo_3* | all | 17.6848213 | 20.439146 | 0.39449663 | 0.49084792 |  |  |  |  |  |  |
| *IbRCo_3* | Paip2014 | 17.9640154 | 19.6205422 | 0.82993126 | 0.80063663 |  |  |  |  |  |  |
| *IbRCo_3* | Paip2015 | 16.9334458 | 20.5385335 | 0.75797301 | 0.76889304 |  |  |  |  |  |  |
| *IbRCo_3* | UPV2015 | 17.1753595 | 20.3971036 | 0.64611967 | 0.65286766 |  |  |  |  |  |  |
| *IbRCo_12* | all | 19.4487277 | 17.624976 | 0.39406565 | 0.51003612 |  |  |  |  |  |  |
| *IbRCo_12* | Paip2014 | 19.7807938 | 17.1982389 | 0.70868046 | 0.9240396 |  |  |  |  |  |  |
| *IbRCo_12* | Paip2015 | 18.7712323 | 18.6548012 | 0.8207784 | 0.78418884 |  |  |  |  |  |  |
| *IbRCo_12* | UPV2015 | 19.7490216 | 16.7859837 | 0.56799288 | 0.80241438 |  |  |  |  |  |  |
| *MaRCo_4* | all | 0.87668954 | -3.28000264 | 0.37625524 | 0.31677612 |  |  |  |  |  |  |
| *MaRCo_4* | Paip2014 | 0.42833798 | -3.92250186 | 0.55624761 | 0.47548134 |  |  |  |  |  |  |
| *MaRCo_4* | Paip2015 | 0.8914752 | -3.47041953 | 0.6237568 | 0.51531452 |  |  |  |  |  |  |
| *MaRCo_4* | UPV2015 | 1.0441178 | -2.21453496 | 0.76462789 | 0.65479677 |  |  |  |  |  |  |
| *MbRCo_19* | all | 22.2068886 | 16.9589337 | 0.78002449 | 0.57767835 |  |  |  |  |  |  |
| *MbRCo_19* | Paip2014 | 23.741821 | 18.3477471 | 1.2986839 | 0.96864578 |  |  |  |  |  |  |
| *MbRCo_19* | Paip2015 | 20.8038686 | 15.1577803 | 1.2326615 | 0.90139374 |  |  |  |  |  |  |
| *MbRCo_19* | UPV2015 | 22.3448916 | 16.6555739 | 1.42553994 | 1.1764202 |  |  |  |  |  |  |
| *MLRCo_1* | all | 60.3779394 | 65.327579 | 1.5179873 | 1.5853791 |  |  |  |  |  |  |
| *MLRCo_1* | Paip2014 | 63.7637478 | 68.4791391 | 2.31452019 | 2.51000867 |  |  |  |  |  |  |
| *MLRCo_1* | Paip2015 | 57.0931215 | 65.9011557 | 2.946847 | 3.67472407 |  |  |  |  |  |  |
| *MLRCo_1* | UPV2015 | 58.804646 | 64.6557087 | 2.76050552 | 2.7310701 |  |  |  |  |  |  |
| *MLRCo_2* | all | 58.9614974 | 66.0779193 | 1.58998324 | 1.49095905 |  |  |  |  |  |  |
| *MLRCo_2* | Paip2014 | 66.7065574 | 64.8030952 | 2.22752984 | 2.68450243 |  |  |  |  |  |  |
| *MLRCo_2* | Paip2015 | 56.1728369 | 64.5617542 | 2.89814631 | 2.69761452 |  |  |  |  |  |  |
| *MLRCo_2* | UPV2015 | 58.6536655 | 64.6168756 | 2.80002484 | 2.68514824 |  |  |  |  |  |  |
| *IaFCo_13* | all | -5.75654135 | -4.8358631 | 0.16555377 | 0.14730254 |  |  |  |  |  |  |
| *IaFCo_13* | Paip2014 | -6.07403744 | -5.34309939 | 0.27308202 | 0.21110343 |  |  |  |  |  |  |
| *IaFCo_13* | Paip2015 | -5.49599874 | -4.89861327 | 0.34763247 | 0.31437923 |  |  |  |  |  |  |
| *IaFCo_13* | UPV2015 | -5.41772727 | -4.55127273 | 0.2521518 | 0.22553142 |  |  |  |  |  |  |
| *MbFCo_19* | all | 23.0268886 | 15.2989337 | 0.36402449 | 0.27167835 |  |  |  |  |  |  |
| *MbFCo_19* | Paip2014 | 23.2541821 | 15.2644775 | 0.59296839 | 0.44490578 |  |  |  |  |  |  |
| *MbFCo_19* | Paip2015 | 23.7038686 | 15.1707803 | 0.63626615 | 0.94659374 |  |  |  |  |  |  |
| *MbFCo_19* | UPV2015 | 23.1519157 | 15.4765739 | 0.65953994 | 0.49564202 |  |  |  |  |  |  |
| *MaFCo_19* | all | -0.52649959 | -2.77633308 | 0.19492317 | 0.17767497 |  |  |  |  |  |  |
| *MaFCo_19* | Paip2014 | -1.96686528 | -3.04955333 | 0.22159234 | 0.19920391 |  |  |  |  |  |  |
| *MaFCo_19* | Paip2015 | -0.00366638 | -2.72559854 | 0.33073722 | 0.30009382 |  |  |  |  |  |  |
| *MaFCo_19* | UPV2015 | 0.36965121 | -2.48671699 | 0.40467338 | 0.36539443 |  |  |  |  |  |  |
| *MaFCo_10* | all | -2.07322581 | -1.44367089 | 0.20557922 | 0.20361816 |  |  |  |  |  |  |
| *MaFCo_10* | Paip2014 | -2.47115385 | -2.66215686 | 0.22101791 | 0.22317423 |  |  |  |  |  |  |
| *MaFCo_10* | Paip2015 | -2.10333333 | -0.92448276 | 0.35696672 | 0.34443764 |  |  |  |  |  |  |
| *MaFCo_10* | UPV2015 | -2.12927083 | -0.31842823 | 0.42706229 | 0.4142407 |  |  |  |  |  |  |
| *MaFCo_13* | all | -2.2155033 | -1.40373812 | 0.21875822 | 0.19128085 |  |  |  |  |  |  |
| *MaFCo_13* | Paip2014 | -2.57145833 | -2.56072727 | 0.23045402 | 0.21528985 |  |  |  |  |  |  |
| *MaFCo_13* | Paip2015 | -2.11854167 | -1.02359375 | 0.38008582 | 0.32916397 |  |  |  |  |  |  |
| *MaFCo_13* | UPV2015 | -0.79679245 | -1.68333333 | 0.41278135 | 0.4479729 |  |  |  |  |  |  |

Means and standard errors of the phenotypic value of RILs belonging to the alternative allelic classes (Zuchini *versus* Scallop) for the markers in the LOD peak regions of each significant QTL. Data of the three environments, combined and separate, are shown to validate the effects of the different QTLs (Paip2014, Paip2015, and UPV2015).
